# Vitamin Metabolism and Its Dependency on Genetic Variations Among Healthy Adults: A Systematic Review for Precision Nutrition Strategies

**DOI:** 10.3390/nu17020242

**Published:** 2025-01-10

**Authors:** Elana Sophie Bösch, Jörg Spörri, Johannes Scherr

**Affiliations:** 1 University Centre for Prevention and Sports Medicine, Department of Orthopaedics, Balgrist University Hospital, University of Zurich, 8008 Zurich, Switzerland; joerg.spoerri@balgrist.ch; 2 Sports Medical Research Group, Department of Orthopaedics, Balgrist University Hospital, University of Zurich, 8008 Zurich, Switzerland

**Keywords:** genetic variations, vitamin metabolism, personalized nutrition, SNPs, homocysteine, vitamin D, B vitamins, antioxidants

## Abstract

Background/Objectives: In recent years, there has been a growing interest in precision nutrition and its potential for disease prevention. Differences in individual responses to diet, especially among populations of different ancestry, have underlined the importance of understanding the effects of genetic variations on nutrient intake (nutrigenomics). Since humans generally cannot synthesize essential vitamins, the maintenance of healthy bodily functions depends on dietary vitamin intake. Understanding the differences in vitamin uptake and metabolism across diverse populations may allow for targeted treatment plans and improved overall health. We assessed the current scientific evidence on genetic variations (such as single-nucleotide polymorphisms (SNPs)) affecting vitamin metabolism in humans. Methods: A systematic literature review of primary studies on genetic variations associated with (personalized) nutrition was conducted. Using key terms related to personalized nutrition, nutrigenomics, SNPs, and genetic variations, three online databases were searched for studies published between 2007 and 2023 that included healthy adult subjects. Only results that were confirmed at least once were included. Study quality was assessed with the Joanna Briggs Institute (JBI) critical appraisal tool. Results: Eighty-six articles were included in this review. Our analysis revealed associations with homocysteine metabolism and B Vitamins, Vitamin D, and components of Vitamin E. Genetic associations with Vitamin D, particularly with the GC gene, were extensively researched and linked to lower 25(OH)D concentrations, with sunlight exposure as a contributing factor. Most variants had a negative effect on homocysteine levels. Additionally, we observed general increases in carotenoid levels in the presence of SNPs, although more research on Selenium and Selenoprotein P concentrations is warranted. No studies on Vitamin C were obtained, indicating an area for further methodological improvement. Ancestry is believed to be a significant factor influencing SNP associations and significance. Conclusions: The current review emphasizes the importance of genetics in targeted disease prevention and health care. Our comprehensive findings may provide healthcare practitioners with reliable information to make recommendations in precision nutrition, specifically vitamin supplementation.

## 1. Introduction

Although the role of nutrition in disease prevention has been neglected in the past, it is of fundamental importance to the mechanisms of non-communicable diseases. As a result, governments worldwide have increasingly adopted general nutritional guidelines, such as the Swiss Food Pyramid, to promote public health and mitigate chronic diseases [1]. Nevertheless, the effectiveness of such a one-size-fits-all approach has recently been called into question [2,3]. Genetic variations have different effects on individuals of different ancestry and oftentimes occur with largely different frequencies, determining the importance of individual SNPs in specific populations. For example, SNPs in the GC gene, which are known to influence Vitamin D levels, vary among populations and can affect Vitamin D metabolism differently. In other words, identical SNPs can affect bioavailability and metabolic responses differently depending on ancestry, even when identical diets are consumed [2,3]. Current dietary guidelines do not consider such inter-individual variances, although they are essential for effective disease prevention.

Precision nutrition (PN) attempts to close this gap by combining patient data, including genetic information, to devise the best-suited diet for populations [4]. While genes may be set, PN argues that nutrition is not, and that based on people’s genotypes, dietary recommendations can serve to prevent the onset of chronic diseases [5]. Given the critical role of dietary vitamins in metabolism and their direct impact on health outcomes, our study focuses on vitamins as key components of personalized nutrition strategies. By investigating how genetic variations influence the metabolism of dietary vitamins, we aim to provide practical and actionable insights for precision nutrition. The combination of nutritional science and genomics—also known as nutrigenomics—has been defined as the field of study “examining how foods affect genes and how individual genetic differences can influence the response to particular nutrients, or other naturally occurring compounds in foods” [6]. Nutrigenomics is a relatively young field; however, scientific research has made considerable progress in recent years, revealing many new associations between genetic variations and human metabolism. To the best of our knowledge, there has been no comprehensive review of recent publications, highlighting the need for a reliable overview of the current state of knowledge in this domain.

## 2. Materials and Methods

This study was conducted according to a protocol previously registered at INPLASY (INPLASY202270068, DOI: https://doi.org/10.37766/inplasy2022.7.0068) and performed according to the principles of the Preferred Reporting Items for Systematic Reviews and Meta-Analyses: the PRISMA Statement [7].

### 2.1. Eligibility Criteria

Eligibility criteria were designed with the Population, Intervention, Comparison, Outcome, Context (PICOC) method (Supplementary Table S2). Briefly, this review included peer-reviewed studies in English or German and focused on the relationship between genetic variations and personalized nutrition (PN). Studies were eligible for inclusion if the cohorts consisted of healthy adult subjects of any physical fitness, ethnicity, or socio-economic status. Furthermore, we considered population-based studies that included, but were not limited to, children, infertile, pregnant, obese, or possibly ill subjects. To provide reliable data, only successfully validated results were included. Studies that dated back more than 15 years, did not observe significant associations, or used large genetic risk scores were excluded. Studies that focused on genetic effects related to nutrient intake and preferences were not considered because of the potential influence of various confounding factors. No restrictions were made regarding outcome measure or follow-up duration (full criteria list in Supplementary Table S1).

### 2.2. Search Strategy

The search strategy for this review was part of a larger analysis and aimed to retrieve any studies reporting associations between genetic variations and nutrients. The selection of studies examining only vitamins was made during title and abstract screening. The search was applied to three electronic databases: Embase, MEDLINE, and the Cochrane Library (Supplementary Table S3). A combination of key words and MeSH terms (Medical Subject Headings) such as genetic variations, SNPs, personalized nutrition, nutrigenomics, and genetic association studies were used. Final searches were concluded on 13 September 2023.

### 2.3. Study Selection and Data Extraction

**Figure 1:**
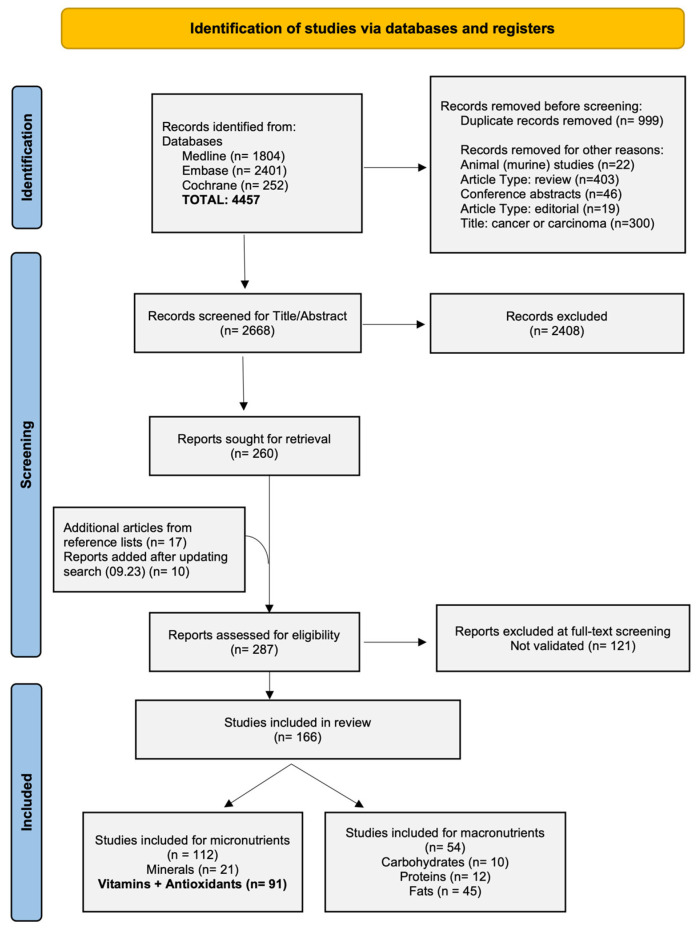
Preferred Reporting Items for Systematic reviews and Meta-analyses (PRISMA) 2020 flow diagram for study selection.

All studies were collected and deduplicated in Endnote and screened for title and abstract before the texts were read in full. The screening process was independently conducted by two authors (EB, JSc) in Rayyan, and uncertainties were discussed after completion [8]. After selecting potential papers from reference lists, 279 were screened for validation, including only those studies whose results were confirmed by at least one other paper. This selection process was conducted by the first author (EB) and peer-reviewed. The study selection and screening process are presented in Figure 1 using a PRISMA flow chart, ensuring transparency in documenting inclusions and exclusions.

Data extraction was performed by means of a customized table that included study characteristics, subject characteristics, data collection methods, outcome measures, and methods of analysis. One author (EB) was responsible for collecting the data, while another author (JSc) reviewed the process.

### 2.4. Risk of Bias Assessment

Study quality was assessed using the JBI critical appraisal tool. The checklists assess quality aspects ranging from inclusion criteria and cohort and setting descriptions to exposure and outcome measurements, as well as validity, confounding factors, and statistical analysis. All studies were assessed by the first author (EB) using the appropriate JBI checklist: these included cross-sectional studies (n = 67), case–control studies (n = 6), RCTs (n = 8), case series (n = 1), quasi-experimental studies (n = 2), and cohort studies (n = 2). The process was reviewed by the last author (JSc).

Quality scores were not used to adjust the inclusion of studies in this review. Instead, the quality scores serve to provide transparency, allowing the reader to assess the potential reliability and biases of the included studies.

## 3. Results

### 3.1. Study Selection and Characteristics

Our systematic search yielded 4457 articles. Duplicates, animal studies, reviews, conference abstracts, editorials, and studies including ‘Cancer’ or ‘Carcinoma’ in their title were removed in Endnote before 2668 papers imported into Rayyan for screening. After initial title and abstract screening, 279 studies underwent further validation and 159 of them yielded results on nutrients that were confirmed by at least one other paper in our review. Of those included studies, 86 examined associations between genetic variations and homocysteine metabolism (n = 32), Vitamin D (n = 48), and antioxidants (n = 6). Findings on SNPs associated with iron were published in a separate review [9]. A PRISMA flow chart (Figure 1) depicts the entire study selection process. All studies used for this review were published as journal articles.

In total, this review found seven SNPs associated with antioxidants, nine related to homocysteine metabolism and B Vitamins, and 43 correlated with Vitamin D. Notably, Du Plessis and colleagues (2020), as well as Nienaber-Rousseau et al. (2013), independently examined the same three SNPs in an identical cohort and therefore drew the same conclusions. Similarly, both studies by Al-Batayneh and colleagues (2019 & 2020) were conducted in the same cohort, but they examined different associations.

### 3.2. Assessment of Risk of Bias

The methodological quality assessment was carried out by the first author (EB) using the appropriate JBI checklist and was peer-reviewed (JSc) to ensure consistency. The checklists and summary scores for each study are presented in the Supplementary Tables S4–S9. Most studies scored 60% or higher, and only five did not meet this threshold. Zero points were awarded for unclear or non-applicable questions.

### 3.3. SNPs Associated with Homocysteine, Folate, and Vitamin B12

**Figure 2:**
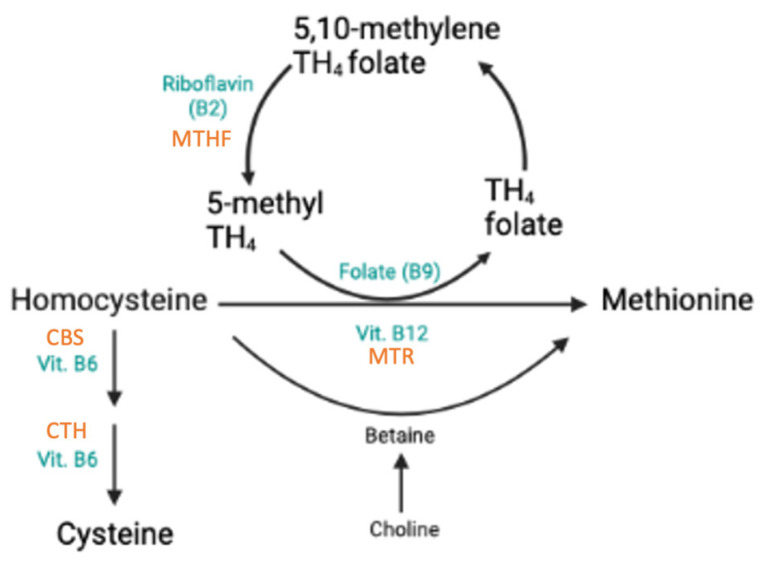
Homocysteine metabolism. Created with BioRender.com.

This review identified ten SNPs significantly associated with components involved in homocysteine metabolism (Figure 2). All associations and outcome measures are compiled in Table S10.

#### 3.3.1. Effects of rs1801133 in MTHFR

The SNP most frequently associated with homocysteine metabolism was rs1801133 (also C677T) in MTHFR. The variant allele T was consistently associated with lower mean red blood cell (RBC) folate values (−4.8%) and explained 3–8% of the variation [10,11]. In contrast, findings in Chinese populations were inconclusive. While one study confirmed previous results (TT: −17.5%), another cohort showed significantly higher RBC folate levels in homozygotes (+12.8%, *p* < 0.05) [12,13]. Compared to wild types, rs1801133 homozygotes experienced 18.2–33.3% lower serum folate levels, with the SNP explaining 3% of the total variation [10,11,14,15,16,17,18]. The interference of sex hormones in this interaction cannot be excluded [10]. Additionally, the SNP appears to have a protective effect, influencing folate metabolism and uptake. This interaction may lead to reduced plasma folate levels, even with sufficient dietary intake [12,19].

When examining its associations with B Vitamins, TT homozygosity reduced Vitamin B6 and B12 levels by 8% and 3.4–6.5%, respectively, and increased the odds of B12 deficiency 1.7–4.2 times [17,18,20,21].

Nineteen studies in this review observed a positive association between rs1801133 and homocysteine (Hcy) [11,12,13,15,16,17,19,22,23,24,25,26,27,28,29,30,31,32,33]. Specifically, the TT genotype increased Hcy levels between 9.5% and 562.7% [17,24,25,26,27,28,29,30,31,32,33]. Consequently, carrying the T allele significantly increased the risk for hyperhomocysteinemia (HHcy), a condition that has been linked to diseases such as atherosclerosis, stroke, and coronary artery disease [30,32,33,34]. Additionally, carrying the T allele increased the odds of HHcy treatment failure by 168% compared to wild types [12,19,23]. The association between rs1801133 and Hcy level was affected by several factors, including dietary folate intake [12,19,23], Vitamin B12 intake [15,33], or diet (vegetarian/non-vegetarian) [27]. Interactions between rs1801133 and other SNPs have frequently been observed to affect associations. A brief overview is presented in Table 1 and comprehensive results are compiled in Tables S10 and S11.

#### 3.3.2. Effects of rs1801131 in MTHFR

Population-specific associations were observed for rs1801131 (A1298C), with differences in genetic effect depending on ethnic background of the studied cohorts. While the variant allele C was associated with 9% lower plasma folate concentrations and 5–13% increased Hcy levels in cohorts of Caucasian and Indian origin, Chinese individuals experienced elevated serum folate (+34.8%) and a reduction in Hcy levels (−32.4%) [14,17,30,32]. Specifically, the CC genotype lowered the odds for HHcy (−80%), as well as the odds for HHcy treatment failure (OR (95% CI): 0.58 (0.41, 0.81)) [23,32]. Rs1801131 showed no significant association with Vitamin B12 deficiency, but more studies are required for validation [20].

#### 3.3.3. Effects of rs2274976 in MTHFR

Two cohorts identified rs2274976 (G1793A) as a significant predictor of RBC folate and plasma folate levels; however, its effect direction seems to depend on ancestry. In Caucasian individuals, carrying the SNP was associated with reduced RBC folate levels, whereas in people of Chinese descent, the A allele increased plasma folate levels by nearly 60% [14,35].

#### 3.3.4. Effects of rs1801394 in MTRR

Rs1801394 (66AG) was the only SNP positively associated with Hcy concentrations in this review. The G allele increased Hcy concentrations by 25–34% and exhibited 1.81 times higher odds of HHcy treatment failure [23,27]. This aligns with results for the rs1801394 x rs162036 haplotype, where the A + G allele carriers showed heightened susceptibility to folic acid therapy compared to the G + A haplotype (OR(95%CI): 0.66 (0.48, 0.91) and OR(95%CI): 1.68 (1.31, 2.15)) [23]. HHcy subjects in the latter group may therefore benefit from an alternative treatment [23].

#### 3.3.5. Effects of rs1805087 (MTR), rs1051266 (RFC1), and 844ins68 (CBS)

Our review revealed three SNPs that significantly reduced Hcy concentrations. The rs1805087 (MTR) G allele was linked with 5–6.6% lower Hcy concentrations in Caucasian and Black African cohorts, although a positive association with Hcy was observed in an Indian population [17,25,27,28]. Homozygosity for the RFC1 variant rs1051266 was associated with 18–26% lower Hcy levels in Indian individuals, with the consumption of a non-vegetarian diet potentially mitigating this effect [27,30]. The CBS variant 844ins68 (LD: rs5742905) reduced Hcy by 2.7–5.1% in Caucasians, while in Black African cohorts, the SNP’s effect depended on factors such as biotin concentration, protein consumption, HDL cholesterol levels, or rs1801133, revealing a complex relationship between nutritional factors and Hcy metabolism [17,25,28].

#### 3.3.6. Effects of rs1801198 in TCN2 and rs34530014 in TCN1

Studies on rs1801198 (C776G) in TCN2 and rs34530014 (TCN1) have highlighted their roles in Vitamin B12 metabolism across different populations. For rs1801198 in TCN2, in Caucasian homozygotes, Vitamin B12 levels rose by 15%, while in Arabs, homozygosity was linked to 5.6 times higher odds of Vitamin B12 deficiency [36,37]. In Hispanic populations, rs1801198 was suggested to reduce Vitamin B12 availability for cellular uptake [38]. The C to G substitution has been suggested to change TCN2’s tertiary structure, potentially affecting the binding capacities of Vitamin B12 and holoTC [37,38]. This SNP was also associated with Hcy, with effects varying based on serum Vitamin B12 levels [36,38].

In contrast, a negative association between rs34530014 (TCN1) and Vitamin B12 levels was specifically observed in individuals of African descent [39,40]. The SNP appears to play a significant role in Vitamin B12 metabolism in this population, although it has not yet been identified in other ethnic groups, suggesting an ethnicity-specific effect [39,40].

#### 3.3.7. Effects of 19 bp Deletion in DHFR

The 19 bp deletion in DHFR is suggested to impair folic acid metabolism, predominantly in interaction with folic acid intake rather than acting independently [35,41,42]. This analysis suggests heterogeneous and context-specific effects, possibly due to population differences and various folic acid fortifications. Nonetheless, the variant is significantly associated with both long- and short-term folate markers, showing a tendency to increase levels with high intake and decrease them with low intake [41]. Hence, this variant is expected to be most relevant to individuals with either very low or very high folic acid consumption. Further research is necessary to better understand this SNP.

### 3.4. SNPs Associated with Vitamin D

The extensive research found on SNPs and Vitamin D can be attributed to the vitamin’s ability to reduce the risk of chronic illnesses, alongside the high global prevalence of Vitamin D deficiency, which underscores its critical clinical relevance [43,44,45]. Given that Vitamin D deficiency affects a significant portion of the population worldwide, understanding the role of genetic variations in Vitamin D status becomes even more essential, as heritable factors are estimated to affect Vitamin D levels by 29% to 80% [46]. Insights gained from these studies are vital for identifying high-risk groups and devising effective precision nutrition interventions.

#### 3.4.1. Polymorphisms in GC

Our analysis identified ten polymorphisms in the GC gene significantly associated with Vitamin D markers, including Calcifediol (25(OH)D), Calcitriol, Vitamin D-binding protein (VDBP), and odds of Vitamin D deficiency (VDD). Variants rs4588, rs7041, rs17467825 and rs3755967, rs2298850, rs2282679, and rs1155563 exhibited negative effects to various degrees, while rs222020 and rs2298849 appeared to increase Vitamin D concentrations [43,44,46,47,48,49,50,51,52,53,54,55,56,57,58,59,60,61,62,63,64,65,66,67,68,69,70,71,72,73,74,75,76,77,78,79,80,81,82,83,84]. The results on SNP rs1212631 were inconclusive [53,60,67]. A summary of the effect sizes on 25(OH)D is given in Table 2; please refer to Table S10 for more detailed values.

Variants rs4588, rs7041, and rs2282679 were the most extensively researched SNPs in GC, all of which exhibited strong negative associations with Vitamin D levels. Homozygosity at rs4588 resulted in 12–27% decreased 25(OH)D levels and a 5–13% reduction in VDBP levels, along with significantly higher odds for VDD [43,46,47,48,49,50,51,52,53,54,56,57,59,60,61,62,63,85,86]. Moreover, carrying the rs4588 T allele increased the odds of non-response to Vitamin D supplementation [87]. The SNP accounted for up to 3.7% of the observed variance in 25(OH)D levels, and its impact appeared to be influenced by factors such as sun exposure [43,49,61,62]. Similarly, variant allele T in rs7041 consistently exhibited negative associations with Vitamin D parameters in twenty-eight diverse cohorts; the variant frequently ranked among the most significant SNPs [43,69,77]. TT homozygotes had 9–32% lower 25(OH)D concentrations and 3.3-fold higher odds of VDD compared to GG genotypes [43,47,49,56,57,62,63,68,72,77,82,83]. The T allele was significantly linked to reduced Calcitriol and VDBP levels and TT homozygotes had 9–32% lower 25(OH)D concentrations and 3.3-times higher odds of VDD compared to GG genotypes [43,47,49,52,56,57,62,63,68,72,77,82,83,84]. Interestingly, observations made for rs7041 in African cohorts differed from those in Caucasians, possibly due to variations in SNP frequencies limiting result generalizability [58,83,85]. These findings largely support a robust negative association between rs7041, rs4588 and Vitamin D; however, a few studies reported no such correlations [47,59,63,83].

In homozygous carriers of the rs2282679 C allele (G on reverse strand), 25(OH)D concentrations were 13–34% lower compared to wild types [46,47,51,53,55,65,68,69,70,71,72,73,75,76,78,80,81]. The variant was further associated with lower VDBP levels, was more prevalent in Vitamin D-deficient people, and increased the odds of VDD by between 50% and 130% [46,47,67,70,72,74,76,81]. Contrasting results were found for rs12512631. The C allele reduced 25(OH)D levels by 4.7% in one study, while two other cohorts observed a positive association, with the variant lowering deficiency risk and increasing 25(OH)D levels by 12% [53,60,67].

#### 3.4.2. Haplotypes in GC

We identified multiple interactions between GC variants that influence Vitamin D markers; however, the interaction between rs4588 and rs7041 was the most extensively studied [60,65]. The haplotypes formed by rs4588 and rs7041 explained 3–7% of the variance in 25(OH)D levels [61,88]. While the Gc2 allele (rs7041 A) was consistently associated with lower 25(OH)D concentrations, the Gc1s-1s haplotype displayed the highest concentrations. Intermediate concentrations were observed in carriers of Gc or Gc1f haplotypes [47,49,55,61]. Moreover, the Gc2 allele was more prevalent among Vitamin D-deficient individuals and was associated with higher odds of deficiency [47,50,52,56,64]. Notably, the ancestral background of the study populations seemed to affect these associations and haplotype frequencies [47,61]. All GC haplotypes and their respective effects can be found in Table S10.

#### 3.4.3. Polymorphisms in CYP2R1

Four of ten identified SNPs in the CYP2R1 gene were negatively associated with Vitamin D; five exerted a positive effect, and one variant presented inconclusive results (Table 3).

Fifteen studies found significant associations between Vitamin D and SNP rs10741657. Homozygous carriers of the A allele presented Vitamin D levels 4.4–53% higher than wild types, as well as 3 times lower odds of VDD [48,49,53,54,56,60,66,68,69,70,77,80,86]. Moreover, the variant was observed to impact Vitamin D levels in interaction with another CYP2R1 variant, rs10766197 [53]. In contrast, two studies linked rs10741657 to lower serum 25(OH)D levels and 4-fold higher odds of being deficient than wild types [55,79]. Five studies conducted in subjects of diverse ancestry found no significant association between rs10741657 and Vitamin D [44,73,74,76,78].

Rs10741657 is in linkage disequilibrium (LD) with rs2060793 in CYP2R1, which has also been positively associated with Vitamin D levels [53,60,62,69,71]. AA homozygotes displayed concentrations 4.5–15% higher compared to wild types [62,71]. Notably, the impact of this association seemed to be influenced by the degree of sun exposure [62]. Furthermore, a haplotype involving CYP2R1 variants rs2060793, rs12794714, and rs10741657 correlated with baseline 25(OH)D levels, where risk alleles in rs10741657 and rs2060793 increased serum levels significantly, confirming previous findings [60].

In contrast, the variant rs12794714 has a negative effect on the Vitamin D marker. Compared to wild types, homozygotes were five times likelier to experience treatment failure and subsequently had 72% higher odds of VDD and 10–20% lower 25(OH)D levels, even after one year of D3 supplementation [49,60,66,68,69,72,73,87]. Similarly, homozygosity for the variant allele A in rs10766197 reduced 25(OH)D levels by 4–10% compared to wild types, also after a year of supplementation [46,53,54,60]. Moreover, the odds ratios for VDD in this variant ranged from 1.215 to 6.533, with homozygous carriers being nearly seven times more likely to experience treatment failure [46,87,89].

The results for rs7116978 were limited and inconclusive. On the one hand, each copy of the T allele was linked to a 7% increase in 25(OH)D; on the other hand, the CC genotype was more responsive to Vitamin D supplementation than carriers of the T allele [53,90]. Notably, when examined in individuals of Arab, South Asian, or Southeast Asian descent, no significant association was observed [68].

**Table 3:** Effects of CYP2R1 variants on 25(OH)D concentrations.

SNP	Effect on 25(OH)D Compared to Wt		Sources
rs10766197	Negative	−4% to −10%	[46,53,54,60]
rs12794714	Negative	−10% to −20%	[49,60,66,68,82]
rs11023374	Negative	−10% to −20%	[62,88]
rs10500804	Negative	−6% to −13%	[62,68,82]
rs2060793	Positive	+4.5% to 15%	[62,71]
rs1562902	Positive	+3 to 9% (per minor allele)	[53,60]
rs1993116	Positive	+6 to 20%	[49,71]
rs731236	Positive	+6% (per minor allele)	[53]
rs10741657 *	Positive	+5 to 53%	[48,49,53,54,56,60,68,70,77]
rs7116978	Inconclusive		[53,90]

Effects refer to homozygous SNP carriers, unless otherwise indicated. * Two studies found a negative association [55,79]. For detailed values, refer to Table S10.

#### 3.4.4. Polymorphisms in CYP24A1

Three variants in CYP24A1 were significantly associated with Vitamin D metabolism in this review. Associations for rs6013897 and Vitamin D were found in three cohorts, and the variant negatively affected 25(OH)D concentrations and reduced the efficacy of year-long Vitamin D3 treatment by 5% [60,70,87]. In contrast, four additional studies found no link between the SNP and Vitamin D, potentially due to their study designs masking the SNP’s small effect size [73,74,76,77]. These results highlight the possibility that CYP24A1 polymorphisms may influence Vitamin D metabolism in a context-specific manner, with interactions between genetic and environmental factors. Contradictory observations were made for both rs2762939 and rs2209314. On the one hand, the variant allele of rs2762939 reduced 25(OH)D concentrations by 10–27%; on the other hand, it exhibited a nearly 3% increase in baseline 25(OH)D [60,63]. A possible explanation for this discrepancy could have been the omission of BMI in one of the analyses [63]. Furthermore, rs2762939 appeared to negatively affect Vitamin D treatment in combination with the CYP24A1 variants rs2244719 and rs4809958 [60].

Rs2209314 demonstrated a comparable pattern. On the one hand, the variant was negatively associated with mean total, free, and bioavailable 25(OH)D concentrations (−16%, −41%, −24%, respectively); on the other hand, it showed positive associations with both VDBP levels (+18%) and serum 25(OH)D levels (+2.7% per C allele) [60,63].

#### 3.4.5. Polymorphisms in VDR

Four SNPs in VDR were significantly associated with Vitamin D in our review. Specifically, variant rs10783219 was linked to lower 25(OH)D levels, resulting in a 3.75% reduction and 40% increased odds for VDD [59,60,67]. Conversely, while one cohort observed a positive association between rs1544410 (BsmI) and 25(OH)D, its examination in two other populations revealed no significant correlation [58,91,92]. Interestingly, in one study, rs1544410 had no direct effect on Vitamin D; however, it did negatively impact the gene expression of VDR and SOD2 while also upregulating CYP24A1, ultimately having an indirect effect on 25(OH)D metabolism [93].

Meanwhile, associations for rs7139166 and rs2228570 appeared to be ancestry-dependent. In Caucasians, the rs7139166 variant allele increased serum 25(OH)D concentrations by 4%, while Amerindians faced 93% higher odds for VDD [60,67]. Similarly, with rs2228570, Cypriot and Arab T allele carriers had 18.7–44.8% lower 25(OH)D levels than wild types, whereas Caucasian individuals responded better to Vitamin D supplementation when carrying the SNP [75,91,92,94]. Notably, this latter association lost significance after Bonferroni correction. Moreover, in a Chinese cohort, the G allele was positively linked to Vitamin D2 and calcium concentrations, while two studies found no significant rs2228570 associations [79,86,95]. Detailed effect sizes are in Table S10.

#### 3.4.6. Polymorphisms in NADSYN1/DHCR7

Both rs3829251 and rs12785878 in NADSYN1/DHCR7 were negatively associated with 25(OH)D in this review. Homozygosity for rs3829251 was significantly associated with reduced 25(OH)D in six cohorts of various ethnicities (view Table S10 for detailed values) [65,71,72]. Similarly, carriers of the rs12785878 SNP exhibited significantly lower 25(OH)D and free−25(OH)D levels compared to wild types, as well as 2.44-times higher odds for VDD [70,72,73,89]. Four cohorts in our review found no significant correlations between rs3829251 and serum 25(OH)D levels, and while four cohorts observed no associations for rs12785878, one study observed a positive association with 25(OH)D [68,74,77,86].

#### 3.4.7. Other Polymorphisms

Engelman et al. (2010) identified a total of 13 SNPs associated with Calciferol (25(OH)D) and Calcitriol (1.25(OH)2D) in their cohort [96]. While the SNPs rs2806508, rs10141935, and rs4778359 were positively associated with Calciferol (+13–24%), variants rs1507023 (in A2BP1) and rs9937918 (in GPR114) reduced Calciferol concentrations by 7% and 9%, respectively [96]. Variants rs6680429 (in DAB1), rs1348864, rs12667374, rs7781309 (MLL3), rs10505337, rs2486443, and rs2154175 were associated with increased Calcitriol levels, effect sizes ranging from 8 to 21.5% [96]. The only SNP negatively correlated with Calcitriol was rs4559029 with an effect of −86.5% in homozygotes [96].

One study observed a negative association between the minor G allele of rs6599638 (in C10orf88) and 25(OH)D levels in three populations [71]. Homozygous carriers exhibited 4.5–7% lower Vitamin D concentrations; however, the authors were not able to replicate these findings in a pooled analysis [71].

### 3.5. SNPs Associated with Antioxidants

This review identified four SNPs within BCO1 that affect Carotenoids. Rs11645428 was linked to reduced β-carotene levels, while increased concentrations were observed in individuals carrying rs6420424, rs8044334, or the G allele in rs6564851 [97,98]. Moreover, the rs6564851 G allele was positively associated with α-carotene levels [97]. In APOA5, a positive association was observed between the variant allele and plasma α-tocopherol. Notably, when adjusting for triacylglyceride, the association strength notably diminished, suggesting that genotype effects might be mediated by elevated circulating lipids [98].

Two polymorphisms in this review were associated with Selenium (Se) and Selenoprotein P (SePP). Ambiguous results were found for rs1050450 (in GPX1), the variant allele increasing Se levels in one cohort, while another study reported a 7% decrease in homozygotes [99,100]. Findings for rs3877899 (in SELENOP) have suggested a negative effect of the T allele on Se and SePP concentrations, with the association possibly being affected by gender [99,100,101,102]. Notably, an interaction between rs3877899 and BMI was observed, possibly impacting the SNP’s effect in certain population groups [102]. For detailed values and effect sizes, refer to Table S11.

## 4. Discussion

This review presents a comprehensive overview of SNPs significantly linked to the metabolism of vitamins in healthy adults. Our analysis yielded studies examining associations among antioxidants, Hcy metabolism, and Vitamin D and contextualized them in relation to precision nutrition. Including studies on diverse populations provided valuable insights into how effects differ across ancestries and emphasized the importance of genetic background in nutritional requirements.

Research on genetic variants affecting antioxidants is limited. Carotenoid levels generally increased in the presence of SNPs; however, effects on Se and SePP were ambivalent and require more extensive research.

Nine SNPs in eight genes significantly affected Hcy metabolism, both independently and in interaction with other variants. Associations were found for Hcy and Vitamins B9 (folate), B12, and B6. The most comprehensively studied SNP was rs1801133, a carrier presenting consistently lower folate, Vitamin B12, and B6 levels, as well as higher Hcy concentrations. The SNP is suggested to render MTHFR more thermolabile, reducing its activity and consequently lowering 5-MTHF concentration [13,33]. As a result, Hcy levels in the blood may increase, posing a greater risk for the development of chronic disease [13]. Moreover, TT homozygotes were less responsive to folic acid supplementation, increasing the risk of folate treatment failure for HHcy [19,23]. Altered intestinal absorption of the vitamin could cause the deficiencies observed in T allele carriers; however, whether the abnormal metabolism is caused by the SNP or not is difficult to determine [20,21]. Lifestyle may also be an influencing factor of B12 deficiency [20]. In conclusion, while diet is a strong determinant of Vitamin B concentrations, variant rs1801133 might help to identify predispositions for impaired Hcy metabolism and vitamin B12 or B6 deficiency. Furthermore, those individuals may benefit from increased B vitamin intake to maintain optimal levels and reduce Hcy concentrations [13,18,103].

In our review, 43 SNPs were identified to be associated with Vitamin D; 17 were positively associated and 19 were negatively associated. Inconclusive results were found for seven polymorphisms. GC was the most extensively researched gene and had the most SNPs associated with Vitamin D. Based on our analysis, most SNPs (8/10) in GC reduce 25(OH)D concentrations, and individuals with those mutations might profit from Vitamin D supplementation, especially during the winter months [48]. Associations between GC and Vitamin D markers were significantly weaker during the winter months compared to summer, suggesting a gene–environment interaction where SNPs interact with UV radiation to impact Vitamin D status [43,51,54,61]. Moreover, GC SNPs lowering 25(OH)D concentrations also correlate with lower VDBP levels; however, it is unclear whether circulating VDBP affects the further metabolism and availability of Vitamin D [70]. Examining the effects of multiple SNPs in GC and CYP2R1, as observed by Barry and colleagues (2014), revealed that while variants affected baseline 25(OH)D levels, they did not affect the response to Vitamin D supplementation. This suggests different regulatory mechanisms for 25(OH)D depending on its source (dietary vs. endogenous) [60].

Differences between study results were observed in our review and may arise from varying methodologies or be due to the relative weight of environmental and lifestyle factors on Vitamin D levels. Additionally, ethnic differences between the study populations and a lack of functionality or low frequency of SNPs in certain groups could be responsible for varying results [58,67]. For example, the GC1s and GC2 isoforms are much more frequent in individuals of European descent compared to Black and Asian subjects, who are more likely to carry the GC1f isoform [47]. This heterogeneity in SNP frequency across diverse populations highlights not only the need for research in diverse cohorts but also the inadequacy of universal nutrition recommendations for individuals of diverse ethnic backgrounds [11,58,68,83]. To ensure the development of more precise and inclusive nutrition guidelines, future studies should prioritize including a broader range of ethnic groups, particularly those under-represented in current research, to better understand the role of genetic factors in vitamin metabolism across populations. Additionally, the protective effect of a non-vegetarian diet in rs1801133 carriers or the influence of sun exposure on Vit D levels emphasizes the importance of considering (cultural) lifestyle factors in personalized nutrition [27,62]. Promising observations were made when using genetic information to design personalized supplementation, resulting in reduced interindividual variability in 25(OH)D levels and smaller impact of genetic variations [48]. These findings highlight the inadequacy of universal Vitamin D recommendations and the potential of genetic analysis to identify both at-risk populations and individuals less responsive to Vitamin D supplementation.

The broad research scope and exclusion of subjects with BMI > 25 kg/^2^ may have introduced some bias to our review. As BMI can affect supplementation responses, it is important to consider overweight individuals in studies on personalized nutrition [102]. Additionally, our decision to exclude studies without observed associations may have introduced bias by limiting the scope of analysis. Moreover, the limited evidence on Vitamin C highlights an opportunity for future search strategies to include broader categories and to potentially expand the scope of this research. Relevant studies, such as Michels et al. (2013) [104] and Niforou et al. (2020) [105], discuss the influence of genetic variations on Vitamin C status and provide additional perspectives on this topic.

While gene–environment interactions such as epigenetics, sun exposure, diet, and physical activity can significantly influence genetic associations, they were not fully explored in this review. Such interactions may play an important role and should be explored in future research, potentially in studies designed to address these complexities more comprehensively.

Lastly, inconsistencies across genetic notations made it difficult to compare study results, highlighting the importance of establishing a standardized notation system for genetic studies. Additionally, methodological heterogeneity among the included studies, such as differences in study design, measurement tools, and outcome definitions, may have contributed to variability in the findings. This underscores the need for more consistent methodologies in future studies to improve comparability and ensure more robust conclusions.

## 5. Conclusions

In conclusion, this review not only emphasizes the significance of SNPs in vitamin metabolism for precision nutrition but also sets the groundwork for personalized approaches, addressing deficiencies and optimizing nutritional interventions regarding vitamins. Our findings may empower health practitioners to identify individuals at risk of therapy failure or vitamin deficiencies and to make well-founded recommendations on vitamin supplementation. In combination with other personal health data, environmental factors, and lifestyle choices, genetic information plays an important role in precision nutrition and has the potential to improve the nutritional status of vulnerable populations [5]. In this regard, the current review may contribute to enhancing the understanding of genetics in nutrition therapy and may serve as a valuable foundation for future research.

## Figures and Tables

**Table 1:** rs1801133 haplotype effects on Hcy.

Haplotype		Effect on Hcy
*rs1801133* *x* *rs1801131*		
T	A *	↑ 72–84% odds of high Hcy
C *	A/C	↓ 44–69% odds of high Hcy
TT	CC	Never observed
*rs1801133* *x* *rs1801394*		
TT	GG	↓ 25.6% compared to TT + AA *
*rs1801133* *x* *T833/844ins68*		
TT	CC	↓ 50% compared to TT + TT *
TT	TC	↓ 39% compared to TT + TT *
*rs1801133* *x* *rs7946*		
TT	GA	↓ 41% compared to TT + AA *
TT	GG	↓ 47% compared to TT + AA *
*rs1801133* *x* *rs9001*		
CC *	CC	↓ 50% compared to TT + AA *

* Wt/major allele. For detailed values, refer to Table S10.

**Table 2:** Effects of GC variants on 25(OH)D concentrations.

SNP	Effect on 25(OH)D Compared to Wt		Sources
rs4588	Negative	−12% to −27%	[43,46,48,49,51,53,54,56,57,60,61]
rs7041	Negative	−9% to −27%	[43,47,49,56,57,60,62,63,68,77,82,83]
rs17467825	Negative	−13.8% to −28.5%	[53,68]
rs3755967	Negative	−17.7% to −27%	[68]
rs2298850	Negative	−16.1% to −28.5%	[68]
rs2282679	Negative	−13% to −34%	[46,47,51,53,68,70,71,75,78,80,81]
rs1155563	Negative	−16% to −28%	[51,60,68]
rs222020	Positive	+3.21% to 8.42% (per minor allele)	[60,66]
rs2298849	Positive	+3.4% to + 13% (per minor allele)	[49,66]
rs12512631	Inconclusive		[53,60,67]

Effects refer to homozygous SNP carriers, unless otherwise indicated. For detailed values, refer to Table S10.

## Data Availability

No new data were created or analyzed in this study.

## Supplementary Materials

The following supporting information can be downloaded at https://www.mdpi.com/article/10.3390/nu17020242/s1, Table S1: Inclusion and exclusion criteria. Table S2: PICOC methodology. Table S3: Search strategies for the systematic literature search. Table S4: JBI Quality Assessment of Cross-Sectional Studies. Table S5: JBI Quality Assessment of Randomized Controlled Trials. Table S6: JBI Quality Assessment of Case-Control Studies. Table S7: JBI Quality Assessment of Quasi-Experimental Studies. Table S8: JBI Quality Assessment of Cohort Studies. Table S9: JBI Quality Assessment of Case Series. Table S10: Vitamins parameter study. Table S11: Antioxidants parameter study.
