# Supplementary material for: Vitamin Metabolism and Its Dependency on Genetic Variations Among Healthy Adults: A Systematic Review for Precision Nutrition Strategies
Source: Nutrients. 2025 Jan 10;17(2):242. doi: 10.3390/nu17020242 (PMC11767394; doi:10.3390/nu17020242)
Supplement: Supplementary file 1 [file nutrients-17-00242-s001.zip › nutrients-3375651-supplementary.pdf]

Supplementary – intended for publication

Abbreviations

Abbreviation	Meaning
BMI	Body Mass Index
Hcy	Homocysteine
HDL-c	HDL-Cholesterol
HHcy	Hyperhomoysteine
LD	Linkage disequilibrium
PICOC	Population, Intervention, Comparison, Outcome, Context, Study method
PN	Personalized nutrition
RBC	Red blood cell
SNP	Single nucleotide polymorphism
VDD	Vitamin D deficiency

Search Strategy

Table S1: Inclusion and exclusion criteria

Inclusion Criteria	*Exclusion Criteria*
• Peer-reviewed primary studies<br>• Languages: English or German<br>• Subjects: healthy adults of any physical fitness, ethnicity, or socioeconomic status<br>• Focused on the aspect of personalized nutrition and genetic variations (e.g. SNPs)<br>• any type of nutrition-based observation and intervention<br>• any outcome measure or follow-up duration	• Animal studies<br>• Children <18 years<br>• Diseased, pregnant, or infertile subjects<br>• Overweight & obese subjects (BMI >25 kg/m^2)<br>• Papers published prior to 2007<br>• Non-validated studies<br>• Studies with nonsignificant correlations<br>• Studies using genetic risk scores<br>• Studies on alcohol intake, food intake and food preferences.<br>• Reviews, meta-analyses, conference abstracts

Table S2: PICOC methodology

PICOC	*Description*
***P**opulation*	adult participants of any age and physical fitness
***I**ntervention*	any types of nutrition-based observation and interventions
***C**omparison*	controlled quantitative studies (any type of controls)
***O**utcome*	any kind of outcome measures and lengths of follow-up
***C**ontext*	clinical and nutrition context

Table S3: Search strategies for the systematic literature search

Database	*Date*	*Concepts*	*Results*
Cochrane	First search: 30.05.2022<br><br>Last search: 05.09.2023	(('single nucleotide' OR gen* OR allel* OR homozygo* OR heterozygo*) NEAR/3 (polymorphism* OR varia* OR pattern* OR diversit* OR mutation* OR differentiat* OR divergenc*)):ti,ab,kw OR ((nutritional* NEAR/3 (genomic* OR genetic*)):ti,ab,kw) OR nutrigenomic*:ti,ab,kw OR<br><br>AND<br><br>((personal* OR precis* OR individuali* OR tailored OR custom*) NEAR/3 (nutri* OR macronutri* OR micronutri* OR alimentary OR metabolism* OR diet* OR feed* OR food* OR intake* OR uptake*)):ti,ab,kw OR ((nutri* OR macronutri* OR micronutri* OR alimentary OR diet* OR feed* OR food*) NEAR/20 (intake* OR uptake* OR advice* OR intervention* OR choice* OR recommend*)):ti,kw OR (daily NEAR/20 (intake* OR uptake*)):ti,kw OR ((deficien* OR defective* OR insufficien*) NEAR/20 (nutri* OR macronutri*<br><br>AND<br><br>((gen* OR allel* OR homozygo* OR heterozygo*) NEAR/4 (interact* OR affect* OR modulat* OR influenc* OR associat* OR correlate* OR determinant* or based OR linkage* OR diathes* OR predisposition* OR prognos* OR propensit* OR proneness* OR anticipation* OR<br><br>Filter:<br>Cochrane Library publication date Between Jan 2007 and Dec 2022	252<br><br>276

EMBASE	First search: 30.05.2022	'single nucleotide polymorphism'/exp OR 'genetic variability'/exp OR 'genetic variation'/de OR 'nutrigenomics'/exp OR 'nutrigenetics'/exp OR ((('single nucleotide' OR gen* OR allel* OR homozygo* OR heterozygo*) NEAR/3 (polymorphism* OR varia* OR pattern* OR diversit* OR mutation* OR differentiat* OR divergenc*)):ti,ab,kw) OR ((nutritional* NEAR/3 (genomic* OR genetic*)):ti,ab,kw) OR nutrigenomic*:ti,ab,kw OR nutrigenetic*:ti,ab,kw<br><br>AND<br><br>'personalized nutrition'/exp OR 'dietary intake'/exp/mj OR 'food intake'/exp/mj OR 'nutritional deficiency'/exp/mj OR 'nutrient uptake'/exp OR (((personal* OR precis* OR individuali* OR tailored OR custom*) NEAR/3 (nutri* OR macronutri* OR micronutri* OR alimentary OR metabolism* OR diet* OR feed* OR food* OR intake* OR uptake*)):ti,ab,kw) OR (((nutri* OR macronutri* OR micronutri* OR alimentary OR diet* OR feed* OR food*)	2401

	Last search: 05.09.2023	NEAR/20 (intake* OR uptake* OR advice* OR intervention* OR choice* OR recommend*)):ti,kw) OR ((daily NEAR/20 (intake* OR uptake*)):ti,kw) OR (((deficien* OR defective* OR insufficien*) NEAR/20 (nutri* OR macronutri* OR micronutri* OR alimentary OR diet* OR feed* OR food*)):ti,kw)<br>AND<br><br>‘gene interaction'/exp OR 'genetic association'/exp OR 'genetic association study'/exp OR 'genetic predisposition'/de OR 'genetic susceptibility'/exp OR (((gen* OR allel* OR homozygo* OR heterozygo*) NEAR/4 (interact* OR affect* OR modulat* OR influenc* OR associat* OR correlate* OR determinant* OR based OR linkage* OR diathes* OR predisposition* OR prognos* OR propensit* OR proneness* OR anticipation* OR susceptibilit*)):ti,ab,kw)<br><br>AND<br><br>([english]/lim OR [german]/lim) AND [2007-2022]/py NOT [conference abstract]/lim NOT ([animals]/lim NOT [humans]/lim) NOT ('juvenile'/exp NOT 'adult'/exp)	2564
MEDLINE (OVID)	First search: 30.05.2022	exp Polymorphism, Single Nucleotide/ or Genetic Variation/ or exp Genomic Structural Variation/ or exp Nutrigenomics/ or (('single nucleotide' or gen* or allel* or homozygo* or heterozygo*) adj3 (polymorphism* or varia* or pattern* or diversit* or mutation* or differentiat* or divergenc*)).ti,ab,kw. or (nutritional* adj3 (genomic* or genetic*)).ti,ab,kw. or nutrigenomic*.ti,ab,kw. or nutrigenetic*.ti,ab,kw.<br><br>AND<br><br>exp *Diet/ or exp *Dietary Supplements/ or exp *Eating/ or exp *Deficiency Diseases/ or ((personal* or precis* or individuali* or tailored or custom*) adj3 (nutri* or macronutri* or micronutri* or alimentary or metabolism* or diet* or feed* or food* or intake* or uptake*)).ti,ab,kw. or ((nutri* or macronutri* or micronutri* or alimentary or diet* or feed* or food*) adj20 (intake* or uptake* or advice* or intervention* or choice* or recommend*)).ti,kw. or (daily adj20 (intake* or uptake*)).ti,kw. or ((deficien* or defective* or insufficien*) adj20 (nutri* or macronutri* or micronutri* or alimentary or diet* or feed* or food*)).ti,kw.<br><br>AND<br><br>exp Genetic Association Studies/ or Genetic Predisposition to Disease/ or ((gen* or allel* or homozygo* or heterozygo*) adj4 (interact* or affect* or modulat* or influenc* or associat* or correlate* or determinant* or based or linkage* or diathes* or predisposition* or prognos* or propensit* or proneness* or anticipation* or susceptibilit*)).ti,ab,kw.	1804

	Last search: 13.09.2023	limit to ((english or german) and yr="2007 - 2022")<br><br>AND<br><br>NOT (animals not humans).sh. NOT ((exp infant/ OR exp child/ OR adolescent/) NOT exp adult/)	1975

JBI Quality Assessment

Red numbers indicate rating scores < 60%

Table S4: Cross-Sectional Studies

	Source / Question	1. Were the criteria for inclusion in the sample clearly defined?	2. Were the study subjects and the setting described in detail?	3. Was the exposure measured in a valid and reliable way?	4. Were objective, standard criteria used for measurement of the condition?	5. Were confounding factors identified?	6. Were strategies to deal with confounding factors stated?	7. Were the outcomes measured in a valid and reliable way?	8. Was appropriate statistical analysis used?	Rating
**Vitamin D**	Zhang et al., 2013	y	y	y	N/A	y	y	y	y	7/8
	Sinotte et al., 2009	y	y	y	N/A	y	y	y	y	7/8
	Santos et al., 2019	y	y	y	N/A	y	y	y	y	7/8
	Robien et al., 2013	y	y	y	N/A	y	y	y	y	7/8
	Rivera-Paredez et al., 2020	y	y	y	y	y	y	y	y	8/8
	Perna et al., 2013	y	y	y	N/A	y	y	y	y	7/8
	Nissen et al., 2014	y	y	y	N/A	y	y	y	y	7/8
	Pooyan et al., 2020	y	y	y	y	y	y	y	y	8/8
	Meshkibaf et al., 2021	n	y	y	y	n	n	y	y	5/8
	Li et al., 2014	y	y	y	y	y	y	y	y	8/8
	Lafi et al., 2015	n	y	y	y	n	n	y	y	5/8
	Fohner et al., 2016	n	y	y	N/A	y	y	y	y	6/8
	Engelman et al., 2008	n	y	y	N/A	y	y	y	y	6/8
	Gozdzik et al., 2011	y	y	y	N/A	y	y	y	y	7/8
	Engelman et al., 2012	y	y	y	N/A	y	y	y	y	7/8
	Lu et al., 2011	y	y	y	y	y	y	y	y	8/8
	Xu et al., 2015	y	y	y	y	y	y	y	y	8/8
	Bu et al., 2010	y	y	y	N/A	y	y	y	y	7/8
	Xu et al., 2014	y	y	y	y	y	y	y	y	8/8
	Szili et al., 2018	y	y	y	N/A	y	y	y	y	7/8
	Batai et al., 2014	n	y	y	y	y	y	y	y	7/8
	Elkum et al., 2014	u	y	y	N/A	y	y	y	y	6/8
	Rivera-Paredez et al., 2018	y	y	y	y	y	y	y	y	8/8
	Engelman et al., 2010	y	y	y	N/A	y	y	y	y	7/8
	Wang et al., 2010	y	y	y	y	y	y	y	y	8/8
	Ahn et al., 2010	u	y	y	N/A	y	y	y	y	6/8
	Lee et al., 2021	y	y	y	N/A	y	y	y	y	7/8
	Kwak et al., 2018	y	y	y	N/A	y	y	y	y	7/8
	Kandemis et al., 2021	y	y	y	y	n	u	y	y	6/8
	Didriksen et al., 2013	y	y	y	N/A	y	y	y	y	7/8
	Trummer et al., 2012	y	y	y	N/A	y	y	y	y	7/8
	Slater et al., 2015	y	y	y	y	y	y	u	y	7/8
	Simon et al., 2011	y	n	y	N/A	y	y	u	y	5/8
	Thongthai et al., 2015	y	y	y	y	y	y	y	y	8/8
	Hansen et al., 2015	y	y	y	N/A	y	y	y	y	7/8
	Tuncel et al., 2019	y	y	y	N/A	n	n	y	y	5/8
	Larcombe et al., 2012	y	y	y	N/A	y	y	y	y	7/8
	Sallinen et al., 2021	y	y	u	N/A	y	y	y	y	6/8
	Man et al., 2022	y	y	n	y	y	y	y	y	7/8
	Grant et al., 2022	y	y	y	N/A	y	y	y	y	7/8
	Parlato et al., 2023	y	y	y	N/A	y	y	y	y	7/8
	Retamoso et al., 2023	y	y	y	y	u	y	y	y	7/8
	Cheung et al., 2013	y	y	y	N/A	y	y	y	y	7/8
**Homocysteine metabolism**	A. Waśkiewicz, 2011	y	y	y	N/A	y	y	y	y	7/8
	E. Zittan, 2007	y	y	y	y	y	y	y	y	8/8
	B. H. Thuesen, 2010	y	y	y	N/A	y	y	y	y	7/8
	Q. H. Yang, 2008	y	y	y	N/A	y	y	y	y	7/8
	J. Ni, 2017	y	y	y	N/A	n	n	y	n	4/8
	M. Lucock, 2012	y	y	y	N/A	y	y	y	n	6/8
	N. Fukuda, 2014	y	y	y	N/A	y	y	y	y	7/8
	A. Fredriksen, 2007	y	y	y	N/A	y	y	u	y	6/8
	J. P. du Plessis, 2019	y	y	y	N/A	y	y	y	y	7/8
	J. Steluti, 2018	y	y	y	N/A	y	y	y	y	7/8
	A. Stanislawska-Sachadyn, 2010	y	y	y	N/A	y	y	y	y	7/8
	Wang et al., 2023	n	y	y	N/A	y	y	y	y	6/8
	M. G. Garrod, 2010	y	y	y	N/A	y	y	y	y	7/8
	K. K. Sukla and R. Raman, 2012	n	y	y	N/A	y	n	y	y	5/8
	F. Wang, 2019	u	y	y	y	y	n	u	y	5/8
	S. M. Naushad, 2017	y	y	y	N/A	y	y	y	y	7/8
	C. Nienaber-Rousseau,2013	y	y	y	N/A	y	y	y	y	7/8
	K. Sun, 2017	y	y	y	N/A	u	u	y	y	5/8
	J. Kumar, 2009	y	y	y	N/A	y	y	y	y	7/8
	M. A. Caudill, 2009	y	y	y	N/A	y	y	y	y	7/8
	H. Arai, A. 2007	n	n	y	N/A	n	n	y	y	3/8
	de Batlle et al., 2016	y	y	y	u	y	y	y	y	7/8
	Stanislawska-Sachadyn et al.,	y	y	y	N/A	u	y	y	y	6/8
	Kalmbach et al., 2008	y	y	y	N/A	y	y	y	y	7/8
	Hu et al., 2018	y	y	y	N/A	y	y	y	y	7/8
**Antioxidants**	Combs et al., 2011	y	y	y	N/A	y	y	y	y	7/8
	Yabuta et al., 2016	y	y	y	N/A	y	y	y	y	7/8
	Ferrucci et al., 2009	y	y	y	N/A	y	y	y	y	7/8

Table S5: Randomized Controlled Trials

Source / Question		1. Was true randomization used for assignment of participants to treatment groups?	2. Was allocation to treatment groups concealed?	3. Were treatment groups similar at the baseline?	4. Were participants blind to treatment assignment?	5. Were those delivering treatment blind to treatment assignment?	6. Were outcomes assessors blind to treatment assignment?	7. Were treatment groups treated identically other than the intervention of interest?	8. Was follow up complete and if not, were differences between groups in terms of their follow up adequately described and analyzed?	9.Were participants analyzed in the groups to which they were randomized?	10. Were outcomes measured in the same way for treatment groups?	11. Were outcomes measured in a reliable way?	12. Was appropriate statistical analysis used?	13. Was the trial design appropriate, and any deviations from the standard RCT design (individual randomization, parallel groups) accounted for in the conduct and analysis of the trial?	Rating
Vitamin D	Nissen, Vogel et al., 2014	u	y	y	y	y	y	y	y	u	y	y	y	y	11/13
	Gaffney-Stomberg et al., 2017	y	y	n	y	y	y	y	y	y	y	y	y	y	12/13
	Barry et al., 2014	y	y	y	y	y	y	y	y	y	y	y	y	y	13/13
	Waterhouse et al., 2014	y	y	y	y	y	y	y	y	y	y	y	y	y	13/13
	Slow et al., 2020	y	y	y	y	y	y	y	y	y	y	y	y	y	13/13
Hcy	Crider et al., 2011	u	y	y	y	y	y	y	n	u	y	y	y	u	9/13
Anti-oxidants	Batai et al., 2021	y	y	y	y	y	y	y	y	y	y	y	y	y	13/13
	Kopp et al., 2018	y	n	y	n	n	n	y	y	y	y	y	y	y	9/13

Table S6: Case-Control Studies

Source / Question		1. Were the groups comparable other than the presence of disease in cases or the absence of disease in controls?	2. Were cases and controls matched appropriately?	3. Were the same criteria used for identification of cases and controls?	4. Was exposure measured in a standard, valid and reliable way?	5. Was exposure measured in the same way for cases and controls?	6. Were confounding factors identified?	7. Were strategies to deal with confounding factors stated?	8. Were outcomes assessed in a standard, valid and reliable way for cases and controls?	9. Was the exposure period of interest long enough to be meaningful?	10. Was appropriate statistical analysis used?	Rating
Vitamin D	Janssens et al., 2010	u	y	y	y	y	y	y	y	N/A	y	8/10
	Mohamed et al., 2022	y	n	n	y	y	n	n	y	N/A	y	5/10
	Liu et al., 2021	n	y	y	y	y	y	y	y	N/A	y	8/10
Hcy	Al-Batayneh et al., 2018 *	y	y	u	y	y	n	n	y	N/A	y	6/10
	Al-Batayneh et al., 2020 *	y	y	y	y	y	y	n	y	N/A	y	8/10
	Rupalika et al., 2018	u	u	y	y	y	n	n	y	N/A	y	5/10

* same cohort

Table S7: Quasi-Experimental Studies

Source / Question		1. Is it clear in the study what is the 'cause' and what is the 'effect' (i.e. there is no confusion about which variable comes first)?	2. Were the participants included in any comparisons similar?	3. Were the participants included in any comparisons receiving similar treatment/care, other than the exposure or intervention of interest?	4. Was there a control group?	5. Were there multiple measurements of the outcome both pre and post the intervention/exposure?	6. Was follow up complete and if not, were differences between groups in terms of their follow up adequately described and analyzed?	7. Were the outcomes of participants included in any comparisons measured in the same way?	8. Were outcomes measured in a reliable way?	9. Was appropriate statistical analysis used?	Rating
Vitamin D	Tomei et al., 2020	y	u	y	n	n	u	u	u	y	3/9
	Ammar et al., 2023	y	y	y	n	n	y	y	y	y	7/9
Hcy	Solis et al., 2008	y	y	y	n	y	y	y	y	y	8/9

Table S8: Cohort Studies

Source / Question		1.Were the two groups similar and recruited from the same population?	2. Were the exposures measured similarly to assign people to both exposed and unexposed groups?	3. Was the exposure measured in a valid and reliable way?	4. Were confounding factors identified?	5. Were confounding factors identified?	6. Were the groups/participants free of the outcome at the start of the study (or at the moment of exposure)?	7. Were the outcomes measured in a valid and reliable way?	8. Was the follow up time reported and sufficient to be long enough for outcomes to occur?	9.Was follow up complete, and if not, were the reasons to loss to follow up described and explored?	10. Were strategies to address incomplete follow up utilized?	11. Was appropriate statistical analysis used?	Rating
Hcy	Du et al., 2018	y	y	y	y	y	y	y	y	y	y	y	11/11
Antioxidants	Méplan et al., 2007	y	y	y	y	y	y	y	y	y	N/A	y	10/11

Table S9: Case Series

Source / Question		1. Were there clear criteria for inclusion in the case series?	2. Was the condition measured in a standard, reliable way for all participants included in the case series?	3. Were valid methods used for identification of the condition for all participants included in the case series?	4. Did the case series have consecutive inclusion of participants?	5. Did the case series have complete inclusion of participants?	6. Was there clear reporting of the demographics of the participants in the study?	7. Was there clear reporting of clinical information of the participants?	8. Were the outcomes or follow up results of cases clearly reported?	9. Was there clear reporting of the presenting site(s)/clinic(s) demographic information?	10. Was statistical analysis appropriate?	Rating
Hcy	Petr et al., 2013	y	y	y	y	n	y	y	y	y	N/A	8/10

Table S10: Vitamins parameter study

	Gene and SNP	Source	Vitamin											Ancestry	Outcome				Risk of bias	Notes
			Vitamin D					Vit. B12	Vit. B9 (Folate)			Vit. B6 (PLP)	Hcy							
			Calcifediol (25(OH)D)	Calcitriol (1,25(OH)2D)	Ergocalciferol (D2)	VDBP	Vit.D deficiency		Red blood cell folate	Folate / Folic acid	Serum / Plasma Folate			Ancestry	Absolute Values	Effect size	Other	P-value	JBI assessment	
Homocysteine metabolism		Solis et al., 2008							x					Hispanic	*Mean ± SEM [nmol/L]*<br><br>CC (Wt): 1704 ± 143<br>TT : 1622 ± 113	CC: 1<br>TT: 0.952		P = 0.015	8/9	
											x				*Mean ± SEM [nmol/L]*<br><br>CC (Wt): 30.4 ± 5.2<br>TT: 23.8 ± 5.2	CC: 1<br>TT: 0.783		P = 0.002		
													x		*Mean ± SEM [µmol/L]*<br><br>CC (Wt): 9.8 ± 0.7<br>TT: 11.5 ± 1.4	CC: 1<br>TT: 1.173		P < 0.0001		
	**MTHFR**<br>rs1801133 (C677T)	Yang et al., 2008 †									x			non-Hispanic white, non-hispanic black, Mexican American	*Adjusted geometric Mean (95%CI) [ng/mL]*<br><br>non-Hispanic White<br>CC (Wt): 6.7 (6.2, 7.2)<br>CT: 6.4 (5.9, 6.8)<br>TT: 5.4 (4.9, 5.8)<br><br>non-Hispanic Black<br>CC (Wt): 4.8 (4.6, 5.1)<br>CT: 4.4 (4.1, 4.7)<br>TT: 3.2 (2.6, 4.0)<br><br>Mexican-American (Hispanic)<br>CC (Wt): 5.5 (5.1, 6.0)<br>CT: 5.0 (4.7, 5.2)<br>TT: 4.4 (4.0, 4.7)	non-Hispanic White<br>CC: 1<br>CT: 0.955<br>TT: 0.806<br><br>non-Hispanic Black<br>CC: 1<br>CT: 0.917<br>TT: 0.667<br><br>Mexican-American (Hispanic)<br>CC: 1<br>CT: 0.909<br>TT: 0.8	Adjusted geometric mean serum folate concentration was ....<br><br>non-Hispanic White<br>19.6% (95% CI: 12.5%,26.2%)<br>non-Hispanic Black<br>33.6% (17.9%, 46.3%)<br>Mexican-American<br>21.3% (15.2%, 27.0%)<br><br>..lower for the TT genotype than the CC wild type	non-Hispanic White<br>P = 0.0002<br><br>non-Hispanic Black<br>P = 0.0052<br><br>Mexican-American<br>P < 0.0001	7/8	
													x		*Adjusted geometric Mean (95%CI) [µmol/L]*<br><br>non-Hispanic White<br>CC (Wt): 8.5 (8.2, 8.7)<br>CT: 9.0 (8.8, 9.2)<br>TT: 10.7 (9.9, 11.4)<br><br>non-Hispanic Black<br>CC: 8.6 (8.4, 8.8)<br>CT: 9.3 (9.0, 9.6)<br>TT: 12.0 (9.1, 15.9)<br><br>Mexican-American (Hispanic)<br>CC: 7.7 (7.4, 8.1)<br>CT: 7.9 (7.7, 8.1)<br>TT: 9.4 (8.9, 10.0)	non-Hispanic White<br>CC: 1<br>CT: 1.059<br>TT: 1.259<br><br>non-Hispanic Black - NS<br><br>Mexican-American (Hispanic)<br>CC: 1<br>CT: 1.026<br>TT: 1.221	Adjusted geometric mean serum folate concentration was ....<br><br>non-Hispanic White<br>26.1% (17.6%, 35.2%)<br>non-Hispanic Black<br>39.7% (5.3%, 85.2%)<br>Mexican-American<br>21.4% (14.6%, 28.6%)<br><br>..higher for the TT genotype than the CC wild type	non-Hispanic White<br>P < 0.0001<br><br>non-Hispanic Black<br>P = 0.0890 (NS)<br><br>Mexican-American<br>P < 0.0001		SNP effect on tHCy signiificantly higher when B12 low.
		Lucock et al., 2013							x					Caucasian		Slope estimate (SE)<br><br>All: -217.5(54.4)<br><br>Subjects for which RBC folate ≥ Median: -216.8 (63.2)		P = 0.0004<br><br>P = 0.0111	6/8	median RBC folate: 848 nmol/L.
		Fukuda et al., 2014 †											x	Asian (Japanese)		MLR<br>*tHcy against folate intake by genotype, ß (95%CI)*<br><br>CC (Wt): - 0.141(-0.251, -0.030)<br>CT: -0.072 (-0.156, 0.012)<br>TT: 0.070 (-0.115, 0.254)<br><br>Simple Linear Regression<br>*tHcy against folate intake by genotype, Coef. (95%CI)*<br><br>CC (Wt): -0.204 (-0.327, -0.080)<br>CT: -0.093 (-0.192, 0.006)<br>TT: 0.109 (-0.090, -0.307)		MLR<br>P = 0.013<br>P = 0.93<br>P = 0.454<br><br>Folate intake * genotype: P = 0.056<br><br>Simple regression<br>P = 0.001<br>P = 0.066<br>P = 0.281	7/8	
	**MTHFR**<br>rs1801133 (C677T)										x					Simple Linear Regression<br>*Plasma folate against folate intake by genotype, Coef (95%CI)*<br><br>CC (Wt): 0.273 (0.107, 0.438)<br>CT: 0.318 (0.188, 0.447)<br>TT: 0.247 (-0.067, -0.561)		P = 0.001<br>P < 0.001<br>P = 0.121		
		de Batlle et al., 2018 †									x			N/R (likely Caucasian)		CC: Ref.<br>CT: -6.48%<br>TT: -15.89%		P = 0.038<br>P < 0.001	7/8	
		Crider et al., 2011									x			Asian (Chinese)	*Mean (95%CI) [nmol/L]*<br><br>CC (Wt): 10.3 (9.7, 11.1)<br>CT: 9.8 (9.5, 10.2)<br>TT: 9.1 (8.7, 9.5)	CC: 1<br>CT: NS<br>TT: 0.883		CC <> TT P = 0.025<br>CC <> CT P = 0.67<br>CT <> TT P = 0.005	7/10	
													x		*Mean (95%CI) [µmol/L]*<br><br>CC (Wt): 6.1 (5.7, 6.4)<br>CT: 6.7 (6.5, 7.0)<br>TT: 10.2 (9.8, 10.6)	CC: 1<br>CT: 1.098<br>TT: 1.672	*Odds fo Hhcy, OR (95%CI)*<br><br>TT: 23.0 (9.2, 57.6)	CC <> CT P = 0.013<br>CC <> TT P < 0.001<br>CT <> TT P < 0.001		
									x						*Mean (95%CI) [nmol/L]*<br><br>CC (Wt): 669.0 (630.6, 709.8)<br>CT: 616.3 (594.7, 638.8)<br>TT: 552.2 (529.7, 575.6)	CC: 1<br>CT: 0.921<br>TT: 0.825		CC <> CT P = 0.06<br>CC <> TT P < 0.001<br>CT <> TT P < 0.001		
		Caudill et al., 2009									x			Hispanic	*Mean ± SEM [nmol/L]*<br><br>CC (Wt): 11.4 ± 0.9<br>TT: 8.0 ± 0.6	CC: 1<br>TT: 0.702		P = 0.004	7/8	
													x		*Mean ± SEM [µmol/L]*<br><br>CC (Wt): 11.6 ± 0.3<br>TT: 30.9 ± 3.1	CC: 1<br>TT: 2.664		P < 0.001		Effect on Hcy:<br>CC interacts with Creatinine<br>TT interacts with folate, riboflavin,
		Ni et al., 2017											x	Asian (Chinese)	*Mean ± SD [µM]* [b]<br><br>CC (Wt): 11.69 ± 3.87<br>CT: 13.32 ± 5.50<br>TT: 21.28 ± 15.22	CC: 1<br>CT: 1.139<br>TT: 1.820		P < 0.01 for both	4/8	HWE equilibrium not met for this SNP<br><br>[b] Interaction depends on folate levels. For results stratified by B12 & folate refer to Ni et al. (2017)
	**MTHFR**<br>rs1801133 (C677T)								x						*Mean ± SD [nM]*<br><br>CC (Wt): 648.14 ± 385.20<br>CT: 811.54 ± 446.41<br>TT: 731.22 ± 388.00	CC: 1<br>CT: 1.252<br>TT: 1.128		TT: P < 0.05		
		Stanisławska et al., 2008 †							x					N/R (likely Caucasian)		*Coef. (SE) [nmol/L]*<br><br>Men<br>CC: Ref.<br>CT: -0.06 (0.02)<br>TT: -0.15 (0.04)<br><br>Women<br>CC: Ref.<br>CT: -0.05 (0.03)<br>TT: -0.10 (0.04)	R2: 0.08<br><br>R2: 0.03	P < 0.01<br><br>P < 0.05	6/8	
											x					*Coef. (SE) [nmol/L]*<br><br>Men*<br>CC: Ref.<br>CT: -0.07 (0.03)<br>TT: -0.10 (0.05)	R2: 0.03	P = 0.05		* results in women not significant

	Gene and SNP	Source	Vitamin												Outcome				Risk of bias	Notes
			Vitamin D					Vit. B12	Vit. B9 (Folate)			Vit. B6 (PLP)	Hcy							
			Calcifediol (25(OH)D)	Calcitriol (1,25(OH)2D)	Ergocalciferol (D2)	VDBP	Vit. D deficiency		Red blood cell folate	Folate / Folic acid	Serum / Plasma Folate			Ancestry	Absolute Values	Effect size	Other	P-value	JBI assessment	
Homocysteine metabolism	MTHFR<br>rs1801133 (C677T)	Zittan et al., 2007											x	Israeli	*Mean ± SD [µmol/l]*<br><br>CC (Wt): 20.6 ± 18<br>CT + TT: 9.4 ± 3.2	CC: 1<br>CT + TT: 0.456	*Prevalence of Hhcy*<br><br>CC: 46%<br>TT + CT: 3.75%	Absolute values<br>P < 0.0001<br><br>Allele freq.<br>P < 0.0001	8/8	Hhcy:<br>Hcy >15µmol/L
								x									*Prevalence of B12 deficiency*<br>CC: 29.8%<br>TT + CT: 9.2%<br><br>*Odds for B12 deficiency, OR(95%CI)*<br>CC: 4.2 (2.1, 8.3)	P < 0.001		B12 deficiency:<br>B12 ≤ 150pmol/L
		Thuesen et al., 2010						x						Caucasian	*Geometric mean (95%CI) [pmol/L]*<br><br>CC (Wt): 293 (288, 297)<br>CT: 287 (282, 292)<br>TT: 274 (264, 285)	CC: 1<br>CT: 0.980<br>TT: 0.935	*Prevalence of B12 < 148pmol/L*<br>CC: 4.9%<br>CT: 4.5%<br>TT: 8.4%<br><br>*Odds for B12 deficiency, OR(95%CI)*<br>TT: 1.78 (1.25, 2.54)	Absolute values<br>P = 0.004<br><br>Prevalence<br>P = 0.001<br><br>OR<br>P = 0.003	7/8	
											x				*Geometric Mean (95% CI) [nmol/L]*<br><br>CC (Wt): 9.3 (9.2, 9.5)<br>CT: 8.6 (8.4, 8.8)<br>TT: 7.5 (7.2, 7.9)	CC: 1<br>CT: 0.925<br>TT: 0.806	*Prevalence of folate < 6.8nmol/L*<br>CC: 27.5%<br>CT: 33.9%<br>TT: 45.9%<br><br>*Odds for low folate, OR(95%CI)*<br>TT: OR (95%CI): 2.24 (1.85, 2.70)	Absolute values<br>P < 0.001<br><br>Prevalence<br>P < 0.001<br><br>OR<br>P < 0.001		
		Wang et al., 2023									x			Asian (Chinese)	*Mean ± SD [µg/L]*<br><br>CC (Wt): 5.00 ± 2.95<br>TC: 6.19 ± 4.74<br>TT: 4.09 ± 2.52	CC: 1<br>CT: 1.238<br>TT: 0.818		P = 0.014	6/8	Values apply to men only
		Al-Batayneh et al., 2018						x						Arab (Jordanian)			*Genotype frequencies by B12 status*<br><br>Deficient<br>CC (Wt): 36%<br>CT: 43%<br>TT: 21%<br>C: 115%; T: 85%<br><br>Sufficient<br>CC (Wt): 46%<br>CT: 47%<br>TT: 7%<br>C: 139%; T: 61%<br><br>X^2= 8.397<br><br>*Odds to be B12 deficient, OR(95%CI)*<br>T allele carrier: 1.684 (1.116, 2.542)<br><br>*Mean age * Vit. B12 deficiency [yrs ± SD]*<br>CC: 31.4 ± 11.9<br>CT: 36.4 ± 14.5<br>TT: 43.7 ± 10.9	Genotype Freq.<br>P = 0.015<br><br>OR<br>P = 0.017<br><br>age * B12 def.<br>P = 0.007	3/5	B12 deficiency:<br>B12 < 200 mg/mL
		Fredriksen et al., 2007 [†]						x						N/R (likely Caucasian)	*Mean (95%CI) [pmol/L]*<br><br>CC (Wt): 335.75 (325.67, 345.83)<br>CT: 329.90 (321.64, 338.17)<br>TT: 324.43 (310.49, 338.38)	CC: 1<br>CT: 0.983<br>TT: 0.966		P = 0.020	6/8	P for trend test
											x				*Mean (95%CI) [nmol/L]*<br><br>CC (Wt): 18.72 (18.14, 19.29)<br>CT: 17.30 (16.83, 17.77)<br>TT: 13.34 (12.54, 14.13)	CC: 1<br>CT: 0.924<br>TT: 0.713		P < 0.001		
												x			*Mean (95%CI) [pmol/L]*<br><br>CC (Wt): 63.19 (60.68, 65.69)<br>CT: 62.44 (60.38, 64.49)<br>TT: 57.96 (54.50, 61.43)	CC: 1<br>CT: 0.988<br>TT: 0.917		P < 0.001		
													x		*Mean (95%CI) [µmol/L]*<br><br>CC (Wt): 10.11 (9.92, 10.31)<br>CT: 10.65 (10.49, 10.81)<br>TT: 13.37 (13.10, 13.65)	CC: 1<br>CT: 1.053<br>TT: 1.322		P < 0.001		
	MTHFR<br>rs1801133 (C677T)	Waśkiewicz et al., 2011[†]											x	Caucasian	*Mean [µmol/L]*<br><br>Men<br>CC/CT: 10.18<br>TT: 13.14<br>Women<br>CC/CT: 8.77<br>TT: 9.77	CC/CT: 1<br>TT: 1.291<br><br>CC/CT: 1<br>TT: 1.114	*Frequency of HHcy by genotype **<br><br>Men<br>CC/CT: 25.8%<br>TT: 52.5%<br>Women<br>CC/CT: 14.9%<br>TT: 22.4%	Absolute values<br>Men P < 0.0001<br>Women P < 0.0001<br><br>Frequencies<br>Men P < 0.0001<br>Women P = 0.0129	7/8	Hhcy:<br>Hcy > 12µmol/L<br><br>* E.g. 25.8% of male carriers of the CC or CT genotype had Hhcy
		Wang et al., 2019											x	Asian (Chinese)	*Mean ± SD [µmol/L]*<br><br>CC (Wt): 12.8 ± 7.5<br>CT: 14.74 ± 6.98<br>TT: 20.92 ± 10.75	CC: 1<br>CT: 1.152<br>TT: 1.634	*Hcy status by genotype **<br><br>Hhcy<br>TT: 47.58%<br>T: 69.17%<br>Hcy normal<br>TT: 21.92%<br>T: 50.58%	Absolute values<br>P = 0.00<br><br>Frequencies<br>P = 0.00	5/8	Hhcy:<br>Hcy ≥ 15 µmol/L<br><br>* E.g. 47.58% of Hhcy subjects had the TT genotype, while 21.92% of subjects with normal Hcy levels were TT homomzygotes.
		Sun et al., 2017 [†]											x	Asian (Chinese)	*Mean ± SD [µmol/L]*<br><br>CC (Wt): 2.53 ± 0.50<br>CT: 2.56 ± 0.40<br>TT: 2.77 ± 0.46	CC: 1<br>CT: 1.012<br>TT: 1.095		P = 0.012	5/8	
		Sukla et al., 2012											x	Asian (Indian)	*Mean [µmol/L]*<br><br>CC (Wt): 11.2<br>CT: 14.6<br>TT: 20.8	CC: 1<br>CT: 1.304<br>TT: 1.857	*Relative risk for HHcy*<br>TT: 35.50	P < 0.0001	5/8	Hhcy:<br>Hcy > 15 µmol/L
		Petr et al., 2013											x	Caucasian	*Mean ± SD [µmol/L]*<br><br>**Baseline**<br>CC (Wt): 5.9±1.3<br>CT: 6.6±1.3<br>TT: 33.2<br><br>**Post-intervention ***<br>CC: 9.9±2.9<br>CT: 11.6±3.3<br>TT: 17.1	CC: 1<br>CT: 1.119<br>TT: 5.627<br><br>CC: 1<br>CT: 1.172<br>TT: 1.727		NS	8/10	* Intervention: 5g/d creatinine for 30 days
		Nienaber-Rousseau et al., 2013 [1]											x	Black-African	*Mean (95%CI) [µmol/L]*<br><br>CC (Wt): 9.74 (9.56, 9.91)<br>CT: 10.5 (10, 10.9)<br>TT: 16.6 (12.9, 21.5)	CC: 1<br>CT: 1.078<br>TT: 1. 704		P < 0.05 for all	7/8	

	Gene and SNP	Source	Vitamin											Ancestry	Outcome				Risk of bias	Notes
			Vitamin D					Vit. B12	Vit. B9 (Folate)			Vit. B6 (PLP)	Hcy							
			Calcifediol (25(OH)D)	Calcitriol (1.25(OH)2D)	Ergocalciferol (D2)	VDBP	Vit. D deficiency		Red blood cell folate	Folate / Folic acid	Serum / Plasma Folate			Ancestry	Absolute Values	Effect size	Other	P-value	JBI assessment	
Homocysteine metabolism		Naushad et al., 2017											x	Asian (Indian)	*Estimated Mean [μmol/L]*<br><br>Men<br>CC: 18<br>CT: 19.5<br>TT: 21<br>Women<br>CC: 13.2<br>CT: 14.7<br>TT: 16.2	Men<br>CC: 1<br>CT: 1.083<br>TT: 1.167<br>Women<br>CC: 1<br>CT: 1.114<br>TT: 1.227	Genotype effect was associated with vegetarian diet only. A non-vegetarian diet compensated the genetic effect, protecting against high Hcy.		7/8	Absolute values are estimated based on Fig. 2 in Naushad et al. (2017)
	**MTHFR**<br>rs1801133 (C677T)	Kumar et al., 2009											x	Asian (Indian)	*Median [μmol/L]*<br><br>**Phase I**<br>Codnominant model<br>CC: 13.4; CT: 13.1; TT: 23.3<br>P(q-value): 0.000458 (0.027)<br>Recessive model<br>CC + CT: 13.3; TT: 23.3<br>P(q-value): 0.0010 (0.024)<br><br>**Phase II**<br>Dominant model<br>CC: 17.8; CT+ TT: 19.9; P= 0.007<br>Allelic association<br>C: 18.0; T: 20.0; P value= 0.0001<br><br>**Pooled sample**<br>Codominant model<br>CC: 15.0; CT: 15.4; TT: 22.0<br>P(q-value): 0.00276 (0.027)<br>Recessive model<br>CC + CT: 15.0; TT: 22.0<br>P(q-value): 0.00165 (0.024)<br>Dominant Model<br>CC: 15.0; CT + TT: 15.9<br>P(q-value): 0.036 (0.15)	**Phase I**<br>Codnominant model<br>CC: 1<br>CT: 0.978<br>TT: 1.739<br><br>Recessive model<br>CC + CT: 1<br>TT: 1.752<br><br>**Phase II**<br>Dominant model<br>CC: 1<br>CT+ TT: 1.118<br>Allelic association<br>C: 1<br>T: 1.111<br><br>**Pooled sample**<br>Codominant model<br>CC: 15.0<br>CT: 1.027<br>TT: 1.467<br>Recessive model		**Phase I**<br>Codnominant model<br>P(q-value): 0.000458 (0.027)<br>Recessive model<br>P(q-value): 0.0010 (0.024)<br><br>**Phase II**<br>Dominant model<br>P= 0.007<br>Allelic association<br>P = 0.0001<br><br>**Pooled sample**<br>Codominant model<br>P(q-value): 0.00276 (0.027)<br>Recessive model<br>P(q-value): 0.00165 (0.024)<br>Dominant Model<br>P(q-value): 0.036 (0.15)	7/8	Phase I<br>546 individuals genotyped and checked for associations between of 14 nsSNPs with Hcy levels.<br><br>Phase II<br>SNPs that were significantly associated with Hcy levels under multiple genetic models were further genotyped in an additional 330 individuals
		du Plessis et al., 2020 [1]											x	Black-African	*Mean (95%CI) [μmol/L]*<br><br>CC (Wt): 10.1 (9.9, 10.3)<br>CT: 11.1 (10.6, 11.6)<br>TT: 18.5 (16.2, 20.7)	CC: 1<br>CT: 1.099<br>TT: 1.832<br><br>*Cohen's d* = 0.22		P = 0.00001	7/8	P value for GLM, remained sugnificant after adjusting for multiple testing
		Arai et al., 2007											x	Asian (Japanese)	*Mean ± SEM [μmol/L]*<br><br>CC (Wt): 10.9 ± 0.3<br>CT: 11.6 ± 0.24<br>TT: 15.7 ± 1.23	CC: 1<br>CT: 1.064<br>TT: 1.440		P < 0.001	3/8	Values were also significant when men & women were analyzed individually (see source for details)
	**MTHFR**<br>rs1801133 (C677T)	Du et al., 2018											x	Asian (Chinese)			*Allele freq. by Hcy therapy status*<br><br>Success grp<br>TT: 47.29%<br>TT + CT: 89.14%<br>T: 55.85%<br>Failure grp<br>TT: 33.85%<br>TT + CT: 77.85%<br>T: 68.21%<br><br>*Odds for treatment failure*<br>*OR (95%CI)*<br><br>TT: 2.68 (1.59, 4.54)<br>CT + TT: 2.12 (1.3, 3.44)<br>T: 1.69 (1.35, 2.13)	P < 0.05 for all<br><br>P < 0.000<br>P = 0.003<br>P = 0.000	10/10	Therapy: 5 mg/d oral folate for 90d<br><br>Success group: patients whose Hcy levels decreased to ≤ 15 μmol/L<br>Failure group: patients whose Hcy levels were ≥15 μmol/L
		Rupalika et al., 2018											x	Asian (Indian)			TT genotype was present in low frequency (0.72%) only among the HHcy case group:<br>OR (95%CI): 0.81 (0.22, 2.91)<br>No TT genotype was observed in Control grp	P = 0.61 (NS)	5/10	The association was not significant due to low sample size (only 21 controls).

Footnotes
Effect directions refer to minor allele T; reference genotype: CC (Wt)
[1] Nienaber-Rousseau et al. (2013) and du Plessis et al (2020) both conducted their studies on the South African arm of the PURE study. Overlaps in subject samples can not be excluded.
[†] Statistical analyses in this study were performed on log-transformed values.
Abbreviations: NS, Not significant; N/R, not reported; SE, standard error; MLR, multilinear regression; OR, odds ratio; R2, coefficient of determination; Coef, Coefficient; HHcy, hyperhomocysteinemia; grp, group

Homocysteine metabolism	rs1801133 x rs1801394	Yang et al., 2008 [†]											x	non-Hispanic white	*Adjusted geometric Mean (95%CI) [μmol/L]*<br><br>TT + GG: 9.3 (8.2, 10.5)<br>TT + AA: 12.5 (10.8, 14.5)	TT + AA: 1<br>TT + GG: 0.744	Adjusted geometric mean serum folate concentration was ....<br><br>25.6% (95% CI: 16.0, 34.4)<br><br>..lower for the 677TT + 66GG haplotype than the TT/AA genotypes	P = 0.0138	7/8	P for trend test

Footnotes
[†] Statistical analyses in this study were performed on log-transformed values.

Homocysteine metabolism	rs1801133 x rs1801131	Wang et al., 2019											x	Asian (Chinese)			*Odds for Hhcy, OR (95%CI)*<br><br>C/A: 0.634 (0.469, 0.859)<br><br>C/C: 0.440 (0.298, 0.648)<br><br>T/A: 1.842 (1.418, 2.394)	P = 0.003<br><br>P < 0.00<br><br>P < 0.00	5/8	Hhcy:<br>Hcy ≥ 15 μmol/L
		Du et al., 2018											x	Asian (Chinese)			*Haplotype freq. by Hcy therapy status*<br><br>Success grp<br>C/A: 28.8%<br>C/C: 15.4%<br>T/A: 55.2%<br>Failure grp<br>C/A: 21.9%<br>C/C: 9.9%<br>T/A: 68.2%<br><br>*Odds for treatment failure*<br>*OR (95%CI)*<br><br>C/A: 0.69 (0.53, 0.89)<br>C/C: 0.60 (0.43, 0.84)<br>T/A: 1.72 (1.36, 2.16)	C/A: P = 0.004<br>C/C: P = 0.003<br>T/A: P = 0.000<br><br>C/A: P = 0.004<br>C/C: P = 0.003<br>T/A: P = 0.000	10/10	Therapy: 5 mg/d oral folate for 90d<br><br>Success group: patients whose Hcy levels decreased to ≤ 15 μmol/L<br>Failure group: patients whose Hcy levels were ≥15 μmol/L
Homocysteine metabolism	rs1801133 x T833C/844ins68	Nienaber-Rousseau et al., 2013											x	Black-African	*Mean (95%CI) [μmol/L]*<br><br>TT x TT: 24.5 (20.5, 28.8)<br>TT x TC: 15 (12, 18.2)<br>TT x CC: 12.2 (1, 21.2)	TT: 1<br>TC: 0.612<br>CC: 0.498		P < 0.01	7/8	Absolute values are estimated based on Fig. 1 in Nienaber-Rousseau et al. (2013)
Homocysteine metabolism	rs1801133 TT x rs7946	Caudill et al., 2009											x	Hispanic	*Mean ± SEM [μmol/L]*<br><br>TT x GG: 42.5 ± 4<br>TT x GA: 25 ± 2.5<br>TT x AA: 22.5 ± 2.5	TT x GG: 1<br>TT x GA: 0.588<br>TT x AA: 0.529	R2: 0.12	P < 0.001 for both	7/8	Absolute values are estimated based on Fig. 4 in Caudill et al. (2009)<br>Co-variable Creatinine excluded from this
Homocystein e metabolism	rs1801133 x rs9001	Kumar et al., 2009											x	Asian (Indian)	*Median [μmol/L]*<br><br>CC x CC: 11.2<br>TT x AA: 22.0	CC x CC: 1<br>TT x AA: 1.964	1.7% of variation explained	P = 0.0002	7/8	

	Gene and SNP	Source	Vitamin											Ancestry	Outcome				Risk of bias	Notes
			Vitamin D					Vit. B12	Vit. B9 (Folate)			Vit. B6 (PLP)	Hcy							
			Calcifediol (25(OH)D)	Calcitriol (1.25(OH)2D)	Ergocalciferol (D2)	VDBP	Vit D deficiency		Red blood cell folate	Folate / Folic acid	Serum / Plasma Folate				Absolute Values	Effect size	Other	P-value	JBI assessment	
Homocysteine metabolism	**MTHFR**<br>rs1801131 (A1298C)	Wang et al., 2019											x	Asian (Chinese)	*Mean ± SD [µmol/L]*<br><br>AA (Wt): 16.99 ± 9.57<br>AC: 16.48 ± 8.48<br>CC: 11.66 ± 5.04	AA: 1<br>AC: 0.970<br>CC: 0.686	*Hcy status by genotype **<br><br>Hhcy<br>A: 80.1%<br>C: 19.90%<br>CC: 1.94%<br>Hcy normal<br>A: 73.46%<br>C: 26.54%<br>CC: 8.85%<br><br>*Odds for Hhcy, OR (95%CI)*<br>A allele: 0.69 (0.50, 0.94)<br>CC: 0.20 (0.07, 0.60)	Absolute values<br>P = 0.01<br><br>Frequencies<br>CC: P = 0.003<br>C: P = 0.02<br>A: P = 0.02	5/8	Hhcy:<br>Hcy ≥ 15 µmol/L<br><br>* E.g. 1.94% of Hhcy subjects had the CC genotype, while 8.85% of subjects with normal Hcy levels were CC homomzygotes.
		Sukla et al., 2012											x	Asian (Indian)	*Mean [µmol/L]*<br><br>AA (Wt): 11.6<br>AC: 12.3<br>CC: 13.1	AA: 1<br>AC: 1.06<br>CC: 1.129			5/8	
		Fredriksen et al., 2007 †											x	N/R (likely Caucasian)	*Mean (95%CI) [µmol/L]*<br><br>AA (Wt): 10.15 (10.02, 10.28)<br>AC: 10.35 (10.24, 10.45)<br>CC: 10.65 (10.51, 10.80)	AA: 1<br>AC: 1.02<br>CC: 1.049		P < 0.001	6/8	P for trend test
															Mean (95%CI) [µmol/L]<br><br>AA (Wt): 37.55 (36.95, 38.15)<br>AC: 37.59 (37.12, 38.06)<br>CC: 36.36 (35.71, 37.02)	AA: 1<br>AC: 1.001<br>CC: 0.968		P = 0.008		
											x				*Mean (95%CI) [µmol/L]*<br><br>AA (Wt): 18.73 (18.16, 19.30)<br>AC: 17.76 (17.32, 18.21)<br>CC: 16.98 (16.35, 17.60)	AA: 1<br>AC: 0.948<br>CC: 0.907		P < 0.001		
		Wang et al., 2023									x			Asian (Chinese)	*Mean ± SD [µg/L]*<br><br>AA (Wt): 4.86 ± 3.56<br>AC + CC: 6.55 ± 4.87	AA: 1<br>AC + CC: 1.348		P = 0.028	6/8	Values apply to men only
		Du et al., 2018											x	Asian (Chinese)			*Allele freq. by Hcy therapy status*<br><br>Failure grp<br>AA (Ref.) : 81.47%<br>AC: 17.25%<br>CC: 1.28<br>CC + AC: 18.53%<br>C: 9.90%<br><br>Success grp:<br>AA (Ref.): 71.39%<br>AC: 25.23%<br>CC: 3.38<br>CC+AC: 28.62%<br>C: 16%<br><br>*Odds for treatment failure compared to AA (Ref.), OR (95%CI)*<br><br>AC: 0.52(0.33, 0.81)<br>CC: 0.26 (0.07, 0.91)<br>AC + CC: 0.48 (0.32, 0.74)<br>C: 0.58 (0.41, 0.81)	AC: P = 0.009<br>CC: NS<br>CC+ AC: P = 0.003<br>C: P = 0.001<br><br>P = 0.004<br>P = 0.035<br>P = 0.001<br>P = 0.001	10/10	Therapy: 5 mg/d oral folate for 90d<br><br>Success group: patients whose Hcy levels decreased to ≤ 15 µmol/L<br>Failure group: patients whose Hcy levels were ≥15 µmol/L

Footnotes
Effect directions refer to minor allele C (G on reverse DNA strand); reference genotype: AA (Wt) (TT on reverse strand)
† Statistical analyses in this study were performed on log-transformed values.
Abbreviations: Hhcy, hyperhomocysteinemia; NS, not significant; OR, odds ratio; grp, group; Ref., reference

	Gene and SNP	Source	Calcifediol (25(OH)D)	Calcitriol (1.25(OH)2D)	Ergocalciferol (D2)	VDBP	Vit D deficiency	Vit. B12	Red blood cell folate	Folate / Folic acid	Serum / Plasma Folate	Vit. B6 (PLP)	Hcy	Ancestry	Absolute Values	Effect size	Other	P-value	JBI assessment	Notes
Homocysteine metabolism	**MTHFR**<br>rs2274976 (G1793A)	Lucock et al., 2013							x					Caucasian		Slope estimate (SE)<br><br>All: -597.8 (173.1)<br><br>Subjects for which RBC folate ≥ Median: -202.5 (87.1)		P = 0.0173<br><br>P = 0.0352<br><br>Subjects for which RBC folate < Median:<br>P = 0.0087	6/8	median RBC folate: 848 nmol/L.
		Wang et al., 2023									x			Asian (Chinese)	*Mean ± SD [µg/L]*<br><br>GG (Wt): 4.96 ± 3.60<br>GA: 7.93 ± 5.96<br>AA: not observed	GG (Wt): 1<br>GA: 1.599		P = 0.006	6/8	Values apply to men only

Footnotes
Effect directions refer to minor allele T (A on reverse DNA strand); reference genotype: CC (Wt) (GG on reverse strand)

	Gene and SNP	Source	Calcifediol (25(OH)D)	Calcitriol (1.25(OH)2D)	Ergocalciferol (D2)	VDBP	Vit D deficiency	Vit. B12	Red blood cell folate	Folate / Folic acid	Serum / Plasma Folate	Vit. B6 (PLP)	Hcy	Ancestry	Absolute Values	Effect size	Other	P-value	JBI assessment	Notes
Homocysteine metabolism	**MTRR**<br>rs1801394 (66AG)	Naushad et al., 2017											x	Asian (Indian)	*Estimated Mean [µmol/L]*<br><br>Men<br>AA (Wt): 18<br>AG: 20.1<br>GG: 22.5<br>Women<br>AA (Wt) : 13.2<br>AG: 15.6<br>GG: 17.7	Men<br>AA: 1<br>AG: 1.117<br>GG: 1.25<br>Women<br>AA: 1<br>AG: 1.182<br>GG: 1.341		N/R	7/8	Absolute values are estimated based on Fig. 2 in Naushad et al. (2017)
		Du et al., 2018											x	Asian (Chinese)			*Allele freq. by Hcy therapy status*<br><br>Success grp<br>G: 24.31%<br>Failure grp<br>G: 36.74%<br><br>*Odds for treatment failure compared to A allele*<br><br>G: OR (95%CI): 1.81 (1.42, 2.30)	P = 0.000<br><br>P = 0.000	10/10	Therapy: 5 mg/d oral folate for 90d<br><br>Success group: patients whose Hcy levels decreased to ≤ 15 µmol/L<br>Failure group: patients whose Hcy levels were ≥15 µmol/L

Footnotes
Effect directions refer to minor allele G; reference genotype: AA (Wt)
Results by Yang et al. (2008) present the effect of rs1801394 AA and GG genotypes in combination with the rs1801133 TT genotype.

	Gene and SNP	Source	Vitamin D: Calcifediol (25(OH)D)	Vitamin D: Calcitriol (1.25(OH)2D)	Vitamin D: Ergocalciferol (D2)	Vitamin D: VDBP	Vitamin D: Vit.D deficiency	Vit. B12	Vit. B9 (Folate): Red blood cell folate	Vit. B9 (Folate): Folate / Folic acid	Vit. B9 (Folate): Serum / Plasma Folate	Vit. B6 (PLP)	Hcy	Ancestry	Outcome: Absolute Values	Outcome: Effect size	Outcome: Other	Outcome: P-value	Risk of bias: JBI assessment	Notes
Homocysteine metabolism	rs1801394 x rs162036	Du et al., 2018											x	Asian (Chinese)			*Allele freq. by Hcy therapy status*<br><br>Success grp<br>A/G: 16.6%<br>G/A: 22.8%<br>Failure grp<br>A/G: 11.7%<br>G/A: 33%<br><br>*Odds for treatment failure OR(95%CI)*<br><br>A/G: 0.66 (0.48, 0.91)<br>G/A: 1.68 (1.31, 2.15)	A/G: P = 0.011<br>G/A: P = 0.000<br><br>A/G: P= 0.011<br>G/A: P = 0.000	10/10	Therapy: 5 mg/d oral folate for 90d<br><br>Success group: patients whose Hcy levels decreased to ≤ 15 µmol/L<br>Failure group: patients whose Hcy levels were ≥15 µmol/L
Homocysteine metabolism	**MTR**<br>rs1805087 (A2756G)	Nienaber-Rousseau et al., 2013 [1]											x	Black-African	*Mean (95%CI) [µmol/L]*<br><br>AA (Wt): 10.1 (9.86, 10.3)<br>AG: 9.5 (9.22, 9.79)<br>GG: 9.5 (8.79, 10.2)	AA: 1<br>AG: 0.941<br>GG: 0.941		P < 0.05	7/8	
		Fredriksen et al., 2007 [†]											x	N/R (likely Caucasian)	*Mean (95%CI) [µmol/L]*<br><br>AA (Wt): 10.90 (10.81, 10.98)<br>AG: 10.65 (10.52, 10.78)<br>GG: 10.36 (10.00, 10.72)	AA: 1<br>AG: 0.977<br>GG: 0.950		P < 0.001	6/8	
		du Plessis et al., 2020 [1]											x	Black-African	*Means (95%CI) [µmol/L]*<br><br>AA (Wt): 10.6 (10.3; 10.8)<br>AG + GG: 9.9 (9.5; 10.2)	AA: 1<br>AG + GG: 0.934<br><br>*Cohen's d* = 0.16	AA: 1<br>AG + GG: 0.934<br><br>*Cohen's d* = 0.16	P = 0.004	7/8	P value for GLM, remained sugnificant after adjusting for multiple testing
		Naushad et al., 2017											x	Asian (Indian)	*Estimated Mean [µmol/L]*<br><br>Men<br>AA (Wt): 18<br>AG: 20.7<br>GG: 23.4<br>Women<br>AA (Wt): 13.2<br>AG: 15.9<br>GG: 18.6	Men<br>AA: 1<br>AG: 1.15<br>GG: 1.3<br>Women<br>AA: 1<br>AG: 1.205<br>GG: 1.409	Genotype effect associated with vegetarian diet, and a non-vegetarian diet compensated genetic effect		7/8	Absolute values are estimated based on Fig. 2 in Naushad et al. (2017)

Footnotes
Effect directions refer to minor allele G; reference genotype: AA (Wt)
[†] Statistical analyses in this study were performed on log-transformed values.
[1] Nienaber-Rousseau et al. (2013) and du Plessis et al (2020) both conducted their studies on the South African arm of the PURE study. Overlaps in subject samples are possible.

	Gene and SNP	Source	Vitamin D: Calcifediol (25(OH)D)	Vitamin D: Calcitriol (1.25(OH)2D)	Vitamin D: Ergocalciferol (D2)	Vitamin D: VDBP	Vitamin D: Vit.D deficiency	Vit. B12	Vit. B9 (Folate): Red blood cell folate	Vit. B9 (Folate): Folate / Folic acid	Vit. B9 (Folate): Serum / Plasma Folate	Vit. B6 (PLP)	Hcy	Ancestry	Outcome: Absolute Values	Outcome: Effect size	Outcome: Other	Outcome: P-value	Risk of bias: JBI assessment	Notes
Homocysteine metabolism	**RFC1**<br>rs1051266 (G80A)	Sukla et al., 2012											x	Asian (Indian)	*Mean [µmol/L]*<br><br>GG (Wt): 12.2<br>GA: 12.6<br>AA: 9.9	GG: 1<br>GA: 1.033<br>AA: 0.811		P < 0.0001	5/8	
		Naushad et al., 2017											x	Asian (Indian)	*Estimated Mean [µmol/L]*<br><br>Men<br>GG: 18<br>GA: 16.6<br>AA: 14.8<br>Women<br>GG: 13.2<br>GA: 11<br>AA: 9.8	Men<br>GG: 1<br>GA: 0.922<br>AA: 0.822<br>Women<br>GG: 1<br>GA: 0.833<br>AA: 0.742	Genotype effect associated with vegetarian diet, and a non-vegetarian diet compensated the genetic effect		7/8	Absolute values are estimated based on Fig. 2 in Naushad et al. (2017)
Homocysteine metabolism	**DHFR**<br>19-bp deletion	Steluti et al., 2018								x				N/R (likely Hispanic)			**19bbp del. * Folate conc.**<br>β (95%CI): 1.02 (1.01, 1.03)	P =0.002	7/8	
		Stanisławska et al., 2008 [†]									x			N/R (likely Caucasian)		*Coef. (SE) [nmol/L]*<br><br>ins/ins: Ref.<br>ins/del: -0.006 (0.04)<br>del/del: 0.12 (0.05)	R2: 0.05	P = 0.02	6/8	The results were only significant in women
		Lucock et al., 2013							x					Caucasian		*β (SE)*<br><br>SNP * Vit. C<br>Wt: NS<br>het.: 0.576 (0.298)<br>homozyg.: 1.380 (0.588)<br><br>SNP * PteGlu<br>Wt: 0.735 (0.378)<br>Het.: 0.745 (0.204)<br>homozyg.: NS	"SNP associated with RBCF in subjects with values >848 nmol/L"	SNP * Vit. C<br>Het.: P = 0.056<br>Homozyg.:P = 0.0245<br><br>SNP * PteGlu<br>Wt: P = 0.0555<br>Het.: P = 0.0004<br><br>SNP * RBCF >848 nmol/L<br>P = 0.0343	6/8	
		Kalmbach et al., 2008 [†]							x					N/R (likely Caucasian)	*Mean (95%CI) [nmol/L]*<br><br>..when folic acid intake <250µg/d<br>Wt: 844.4 (787.8, 905.1)<br>ins/del: 793.0 (750.3, 837.4)<br>del/del: 732.3 (669.1, 801.4)	Wt: 1<br>het.: 0.939<br>hom.: 0.867		del/del: P < 0.05	7/8	
										x							*Prevalence of high folic acid when intake >500µg/d*<br>Wt: 24.4%; 95% CI: 13.9, 35.0<br>ins/del: 21.4%, 95% CI: 13.6, 29.3<br>del/del: 47.0%, 95% CI: 31.6, 62.5	ins/del: P < 0.05<br>del/del: P < 0.05		high folic acid: >1.35nmol/L

Footnotes
Effect directions refer to the double deletion (del/del); reference genotype: double insertion (ins/ins; Wt)
[†] Statistical analyses in this study were performed on log-transformed values.
Abbreviations: Coef, Coefficient; SE, standard error; Ref., Reference; PteGlu, Pteroylmonoglutamic acid (= folic acid)

	Gene and SNP	Source	Vitamin: Vitamin D: Calcifediol (25(OH)D)	Vitamin D: Calcitriol (1.25(OH)2D)	Vitamin D: Ergocalciferol (D2)	Vitamin D: VDBP	Vitamin D: Vit.D deficiency	Vit. B12	Vit. B9 (Folate): Red blood cell folate	Vit. B9 (Folate): Folate / Folic acid	Vit. B9 (Folate): Serum / Plasma Folate	Vit. B6 (PLP)	Hcy	Ancestry	Outcome: Absolute Values	Outcome: Effect size	Outcome: Other	Outcome: P-value	Risk of bias: JBI assessment	Notes
Homocysteine metabolism	**TCN2**<br>rs1801198 (C776G)	Stanisławska et al., 2010						x						Caucasian	*Median (IQR), [pmol/L]*<br><br>CC (Wt): 243.5 (182.8–333.0)<br>CG: 269.7 (212.8–343.7)<br>GG: 279.7 (213.9–351.5)	Wt: 1<br>CG: 1.11<br>GG: 1.15		P overall = 0.01<br>CG: P = 0.03<br>GG: P = 0.045	7/8	Bonferroni corrected P-values<br>P overall = P value by Kruskal-Wallis
													x		*Median (IQR), [pmol/L]*<br><br>…when B12 ≤ 261.96 pmol/L<br>CC (Wt): 7.5 (6.1–9.4)<br>CG: 7.4 (6.2–8.9)<br>GG: 8.5 (6.5–10.9)	CC: 1<br>CG: 0.987<br>GG: 1.133		P overall = 0.04<br>CG: P = 0.01 *<br>CC + CG: P = 0.06		Bonferroni corrected P-values<br>P overall = P value by Kruskal-Wallis<br>* unadjusted
		Garrod et al., 2010 †						x						Hispanic	*Mean ± SD, [%]*<br><br>**HoloTC/B12 Ratio**<br>CC (Wt): 26.5 ± 0.1<br>GG: 22.8 ± 0.1	CC: 1<br>GG: 0.860		P = 0.04	7/8	
													x		*Mean (95%CI), [μmol/L]*<br><br>…when holoTC < 35pmol/L<br>CC (Wt): 17 (11, 23)<br>CG + GG: 14 (11.5, 16)	Wt: 1<br>CG + GG: 0.824	*Odds for Hhcy, OR (95%CI)*<br><br>**Model 1** (incl. B12)<br>Total B12 * genotype<br>5.41 (1.08, 27.0)<br>CG+GG (compared to CC)<br>18.8 (1.04, 339)<br><br>**Model 2** (incl. holoTC)<br>holoTC * genotype<br>5.99 (1.14, 31.6)<br>CG+GG (compared to CC)<br>27.9 (1.33, 584)	Absolute values<br>P = 0.02<br><br>OR Model 1<br>P = 0.04<br><br>P = 0.05<br><br>OR Model 2<br>P = 0.03<br><br>P = 0.03		Absolute values are estimated based on Fig. 2 in Garrod et al. (2010)<br><br>Hhcy:<br>Hcy >13 μmol/L
		Al-Batayneh et al., 2020						x						Arab			*Genotype frequencies by B12 status*<br><br>Deficient<br>GG: 15%<br>G: 28%<br>Sufficient<br>GG: 0%<br>G: 6.5%<br><br>*Odds for B12 deficiency, OR (95%CI)*<br>5.6 (2.95, 10.63)	GG: P = 0.0001<br>G: P = 0.0001<br><br>P = 0.0001	8/10	B12 deficiency:<br>B12 < 200 mg/mL

Footnotes
Effect directions refer to minor allele G; reference genotype: CC (Wt)
† Statistical analyses in this study were performed on log-transformed values.
Abbreviations: IQR, interquartile range; holoTC, Holotranscobalamin (bioavailable B12); OR, odds ratio; Hhcy, hyperhomocysteinemia, freq., frequency;

	Gene and SNP	Source	Calcifediol (25(OH)D)	Calcitriol (1.25(OH)2D)	Ergocalciferol (D2)	VDBP	Vit.D deficiency	Vit. B12	Red blood cell folate	Folate / Folic acid	Serum / Plasma Folate	Vit. B6 (PLP)	Hcy	Ancestry	Absolute Values	Effect size	Other	P-value	JBI assessment	Notes
Homocysteine metabolism	**CBS**<br>844ins68 (LD with rs5742905 (T833C))	du Plessis et al., 2020											x	Black-African			*844ins68 * biotin intake, Slope ± SE [μg/day]*<br>TT (Wt): −0.02 ± 0.01<br>CT: −0.01 ± 0.01<br>CC: 0.03 ± 0.02<br><br>*844ins68 * HDL-c, Slope ± SE [mmol/L]*<br>TT (Wt): 1.73 ± 0.24<br>CT: 0.91 ± 0.26<br>CC: −0.17 ± 0.57	SNP * biotin intake<br>Interaction P = 0.04<br>Slope P = 0.05 for all genotypes<br><br>SNP * HDL-c<br>Interaction P = 0.001<br>Slope P-value<br>TT: P< 0.000001<br>CT: P = 0.001<br>CC: P = 0.77	7/8	
		Fredriksen et al., 2007 †											x	N/R (likely Caucasian)	*Mean (95%CI) [μmol/L]*<br><br>Wt: 10.90 (10.78, 11.02)<br>het.: 10.61 (10.38, 10.83)<br>hom: 10.35 (9.74, 10.96)	Wt: 1<br>het.: 0.973<br>hom.: 0.949		P < 0.001	6/8	HWE not met for this SNP in this cohort

Footnotes
Effect directions refer to minor allele C; reference genotype: TT (Wt)
† Statistical analyses in this study were performed on log-transformed values.

	Gene and SNP	Source	Calcifediol (25(OH)D)	Calcitriol (1.25(OH)2D)	Ergocalciferol (D2)	VDBP	Vit.D deficiency	Vit. B12	Red blood cell folate	Folate / Folic acid	Serum / Plasma Folate	Vit. B6 (PLP)	Hcy	Ancestry	Absolute Values	Effect size	Other	P-value	JBI assessment	Notes
Homocysteine metabolism	**TCN1**<br>rs34530014	Hu et al., 2018						x									Association with B12, *β ± SE*<br><br>JHS cohort: - 0.817 ± 0.104<br>BioVU cohort: - 0.158 ± 0.038<br>BioME cohort: - 0.272 ± 0.037	P= 6.48E-15<br>P= 3-28E-5<br>P= 2.76E-13	7/8	

Footnotes
Rs34530014 was not directly genotyped or imputed in BioVU or BioMe, therefore LD proxy rs11822978 (r2=0.98 in JHS) was used to confirm its association with lower Vit. B12 levels in BioVU and BioMe (n= 3,924)
Effect directions refer to minor allele A; reference genotype: AC (Wt) for rs34530014 and minor allele T; reference genotype: CC (Wt) for rs11822978

	Gene and SNP	Source	Calcifediol (25(OH)D)	Calcitriol (1.25(OH)2D)	Ergocalciferol (D2)	VDBP	Vit.D deficiency	Vit. B12	Red blood cell folate	Folate / Folic acid	Serum / Plasma Folate	Vit. B6 (PLP)	Hcy	Ancestry	Absolute Values	Effect size	Other	P-value	JBI assessment	Notes
Vitamin D	**GC**<br>rs4588	Zhang et al., 2013	x											Asian (Chinese)	*Mean [ng/mL]*<br><br>GG (Wt): 20.3<br>GT: 19.55<br>TT: 17.88	Wt: 1<br>GT: 0.963<br>TT: 0.881	β = -0.1635<br><br>*Odds for VDD, OR (95%CI)*<br>1.403 (1.245–1.583)	P = 0.002016 *<br><br>OR: P = 2.7E-8	7/8	* Bonferroni corrected
						x									*Mean [mg/mL]*<br><br>GG (Wt): 317<br>GT: 307<br>TT: 300	GG: 1<br>GT: 0.968<br>TT: 0.946				Values estimated based on Fig. 4 in Zhang et al. (2013)
	**GC**<br>rs4588	Sinotte et al., 2009	x											Caucasian	*Mean ± SE [nmol/L]*<br><br>CC (Wt): 67.2 ± 0.9; Ref.<br>CA: 63.2 ± 0.9; P = 0.0018<br>AA: 58.4 ± 2.0; P < 0.0001<br><br>Seasonal Association<br>**May-Oct:**<br>CC: 74.1 ± 1.1<br>CA: 69.3 ± 1.3<br>AA: 61.5 ± 2.7<br><br>**Nov-Apr**<br>CC: 58.2 ± 1.3<br>CA: 54.8 ± 1.5<br>AA: 52.7 ± 3.2	CC (Wt): 1<br>CA: 0.940<br>AA: 0.869<br><br>**May-Oct**<br>CC: 1<br>CA: 0.935<br>AA: 0.830<br><br>**Nov-Apr**<br>CC: 1<br>CA: 0.942<br>AA: 0.905	**Total**<br>β ± SE = 24.22 ± 0.93<br>**May-Oct**:<br>β = -5.73<br>**Nov-Apr**<br>β= -3.03	Total<br>P for trend < 0.0001<br>Summer<br>P for trend < 0.0001<br>Winter<br>P for trend = 0.037	7/8	
		Santos et al., 2019				x								Caucasian (20% African + Caucasian mixed)	*Mean ± SD [μg/mL]*<br><br>CC (Wt): 202.98±28.28<br>CA: 196.49±29.88<br>AA: 183.95±36.85	CC (Wt): 1<br>CA: 0.968<br>AA: 0.906	β = -8.4	P < 0.001	7/8	

	Gene and SNP	Source	Vitamin												Outcome				Risk of bias	Notes
			Vitamin D					Vit. B12	Vit. B9 (Folate)			Vit. B6 (PLP)	Hcy							
			Calcifediol (25(OHD)	Calcitriol (1.25(OH)2D)	Ergocalciferol (D2)	VDBP	Vit.D deficiency		Red blood cell folate	Folate / Folic acid	Serum / Plasma Folate			Ancestry	Absolute Values	Effect size	Other	P-value	JBI assessment	
Vitamin D	GC<br>rs4588	Sallinen et al., 2021[†]	x											Caucasian	*Mean ± SD [nmol/L]*<br><br>CC (Wt): 69.3 ± 13.2<br>CA: 65.7 ± 12.6<br>AA: 60.5 ± 10.8	CC (Wt): 1<br>CA: 0.948<br>AA: 0.873	β = -3.7	P = 1.2E-22	6/8	
		Robien et al., 2013 [†]	x											Asian (Chinese)	*Geometric Mean (95%CI) [nmol/L]*<br>CC (Wt): 68.7 (66.7, 70.8)<br>CA: 64.3 (61.9, 66.8)<br>AA: 57.2 (52.8, 61.9)	CC (Wt): 1<br>CA: 0.936<br>AA: 0.833	Variance explained: 3.7%	P for trend < 0.001	7/8	
		Rivera-Paredez et al., 2020					x							Hispanic			"rs4588 was positively associated with higher odds of VDD"<br><br>AA & AC had higher prevalence of VDD compared to CC	AA: P = 0.003<br>AC: P = 0.021	8/8	VDD:<br>25(OH)D <20 ng/mL
	GC<br>rs4588	Perna et al., 2013	x											Caucasian	*Median (IQR), [nmol/L]*<br><br>**Women**<br>CC (Wt): 41.1 (31.3; 53.7)<br>CA: 39.1 (30.3; 50.1)<br>AA: 34.9 (29.7; 47.5)<br>**Men**<br>CC (Wt): 55.1 (39.2; 73.4)<br>CA: 51.5 (35.8; 69.8)<br>AA: 44.3 (33.9; 62.7)	**Women**<br>CC (Wt): 1<br>CA: 0.951<br>AA: 0.849<br><br>**Men**<br>CC (Wt): 1<br>CA: 0.935<br>AA: 0.804	*Adjusted Estimates (SE) [nmol/L]*<br><br>CC (Ref.): 0.0<br>CA: -2.3 (0.7)<br>AA: -5.5 (1.4)		7/8	
		Pooyan et al., 2020					x							Iranian			**Allele Freq.**<br>CC (Wt)<br>VDD: 70.4% <> Normal: 29.6%<br>AC+AA<br>VDD: 75.3% <> Normal: 24.7%		8/8	VDD:<br>Vit D < 30ng/mL<br><br>SNP was not associated with Vit.D in binary regresison model
		Nissen, Vogel et al., 2014 [1,†]	x											Caucasian	*Geometric Mean (95%CI) [nmol/L]*<br><br>CC (Wt): 75.1 (73.1, 77.2)<br>CA: 69.9 (67.7, 72.1)<br>AA: 61.2 (56.9, 62.9)	CC (Wt): 1<br>CA: 0.927<br>AA: 0.812		P < 0.0001	6/8	
		Nissen et al., 2014 [1,†]	x											Caucasian	*Geometric Mean (95%CI) [nmol/L]*<br><br>CC (Wt): 74.1 (71.0, 77.3)<br>CA: 69.7 (66.3, 73.2)<br>AA: 63.6 (57.1, 70.8)	CC (Wt): 1<br>CA: 0.941<br>AA: 0.858		P = 0.0008	7/8	
		Li et al., 2014	x											Asian (Chinese)			β (SE): -3.084 (0.899)	P = 0.0006<br>P = 0.005 *	8/8	* Bonferroni corrected
		Lafi et al., 2015	x											Mostly Caucasian (Mixed)	*Mean ± SD [ng/mL]*<br><br>CC (Wt): 32.7 ± 20.9<br>CA: 24.6 ± 20.0<br>AA: 12.4 ± 6.9<br><br>CC + AC: 28.7 ± 20.5<br>AA + AC: 18.5 ± 15.6	CC (Wt): 1<br>CA: 0.752<br>AA: 0.379<br><br>CC + AC: 1<br>AA + AC: 0.645	*Odds for low Vit. D, OR (95%CI)*<br><br>CC (Wt):Ref.<br>CA: 2.60 (1.36, 4.96)<br>AA: 11.58 (1.39, 96.78)<br><br>CC+ AC: 7.95 (4.48, 14.12)<br>AA + AC: 3.01 (1.61, 5.61)	CA: P= 0.004<br>AA: P = 0.024<br><br>CC + AC: P < 0.0001<br>AA + AC: P = 0.0005	5/8	
	GC<br>rs4588	Jannsens et al., 2010 [2]	x											Caucasian	*Mean ± SD [ng/mL]*<br><br>CC (Wt): 21.0 ± 7.9<br>CA: 19.2 ± 7.0<br>AA: 15.6 ± 7.2	CC (Wt): 1<br>CA: 0.914<br>AA: 0.743	Multivariate analysis:<br>association of rs4588 with 25-OHD when correcting for age, gender, current smoking status, BMI and seasonal variation	Univariate analysis<br>CA: P = 0.15 (NS)<br>AA: P = 0.01<br><br>Multivariate analysis<br>controls: P = 0.03<br>patients: P = 0.05	8/10	
		Gaffney-Stomberg et al., 2017	x											Caucasian			Discovery cohort<br>β (SE) = –2.53 (0.797)<br>Replication<br>β (SE) = –3.952 (0.955)	Discovery cohort<br>P = 0.0016<br>Replication<br>P = 4.97E-5 *<br>P = 0.0013 **	7/8	* FDR corrected<br>** Bonferroni corrected
		Engelman et al., 2008	x											Hispanic & African American		Effect of SNP, *Coef ± SE*<br><br>San Antonio Hispanics<br>0.199 ± 0.068<br>San Luis Hispanics<br>0.285 ± 0.075<br>Los Angeles African Americans<br>0.234 ± 0.087		P = 0.004<br><br>P < 0.001<br><br>P = 0.007	6/8	
				x												Effect of SNP, *Coef ± SE*<br><br>San Antonio Hispanics<br>0.255 ± 0.093<br>San Luis Hispanics<br>0.182 ± 0.085		P= 0.007<br><br>P=0.032		
		Barry et al., 2014 [†]	x											Caucasian			Estimated diff. In serum level per variant allele: -8.87%<br>95%CI: -10.90, -6.78	P < 0.0001 (Wald test)	7/8	
	GC<br>rs4588	Gozdzik et al., 2011 [†]	x											Diverse (East & South Asian, Europan, other)	*Mean ± SD [nmol/L]*<br><br>**East Asians**<br>Fall:<br>CC: 54.4 ± 15.7<br>AC: 42.5 ± 12.0<br>AA: 34.2 ± 6.50<br>Winter:<br>CC: 31.3 ± 17.1<br>CA: 29.5 ± 12.5<br>AA: 24.4 ± 7.39<br><br>**Europeans**<br>Fall:<br>CC: 81.4 ± 26.1<br>CA: 73.4 ± 22.6<br>AA: 59.6 ± 20.6	**East Asians**<br>Fall<br>CC (Wt): 1<br>AC: 0.781<br>AA: 0.629<br>Winter:<br>CC (Wt): 1<br>AC: 0.942<br>AA: 0.780<br><br>**Caucasians**<br>Fall:<br>CC (Wt): 1<br>AC: 0.902<br>AA: 0.732	*A allele partial correlations*<br><br>**East Asians**<br>Fall: -0.463,<br>Winter: -0.384<br>**Europeans**<br>Fall: -0.256	P < 0.001<br>P < 0.001<br><br>P = 0.009	7/8	

	Gene and SNP	Source	Vitamin												Outcome				Risk of bias	Notes
			Vitamin D					Vit. B12	Vit. B9 (Folate)			Vit. B6 (PLP)	Hcy							
			Calcifediol (25(OH)D)	Calcitriol (1.25(OH)2D)	Ergocalciferol (D2)	VDBP	Vit.D deficiency		Red blood cell folate	Folate / Folic acid	Serum / Plasma Folate			Ancestry	Absolute Values	Effect size	Other	P-value	JBI assessment	
Vitamin D	**GC**<br>rs4588	Engelman et al., 2012	x											Caucasian	*Mean stratified by season*[3] *[nmol/L]*<br><br>High exposure<br>CC (Wt): 65.6<br>CA: 62.0<br>AA: 53.5		Additive genetic model<br>β = -0.25<br><br>SNP * High exposure<br>β = -0.33	P < 0.001<br><br>High exposure<br>P = 0.0002	7/8	
Vitamin D	**GC**<br>rs4588	Ammar et al., 2023	x											Arab			*Odds of non-response *, OR (95%CI)*<br><br>GG (Wt): 1<br>GT: 7.58 (1.97, 27.2)<br>TT: 11.51 (3.36, 39.4)	P = 0.002<br>P < 0.001	7/9	* non-response: serum 25(OH)D <30ng/mL after Vit. D supplementation
Vitamin D	**GC**<br>rs4588	Grant et al., 2022	x											Mixed		*Adjusted Mean Difference (95%CI), [nmol/L]*<br><br>TT: Ref. (0.00)<br>TG: 6.12 (3.71, 8.77)<br>GG (Wt): 10.94 (8.52, 13.69)		both P < 0.001	7/8	
Vitamin D	**GC**<br>rs4588	Parlato et al., 2023	x											Black-African		*UK Biobank Sample*<br>β (SE) = -0.11 (0.01)<br><br>*SCCS Sample **<br>β (SE)= -0.12 (0.06)	Variance explained: 0.01%	P = 1.48E-13<br>P = 2.28E-02	7/8	* Southern Community Cohort Study
Vitamin D	**GC**<br>rs4588	Parlato et al., 2023				x								Black-African		*SCSS Sample **<br>β (SE)= 0.13 (0.05)		P = 0.01	7/8	* Southern Community Cohort Study
Vitamin D	**GC**<br>rs4588	Szili et al., 2018				x								Caucasian	*Mean [mg/L]*<br><br>GG (Wt): 320.2<br>GT: 305.5<br>TT: 277.3	GG: 1<br>GT: 0.954<br>TT: 0.866		TT: P = 0.004 *	7/8	* remained signif. After FDR correction
Vitamin D	**GC**<br>rs4588	Lu et al., 2011 †	x											Asian (Chinese)			GLR<br>Beijing: β (SE) = -0.087 (0.01)<br>Shanghai: β (SE) = -0.064 (0.01)<br>Meta-analysis: β (SE) = -0.076 (0.01)<br><br>Conditional analyses<br>Beijing: β (SE) = -0.086 (0.02)<br>Shanghai: β (SE) = -0.057 (0.02)<br>Meta-analysis: β (SE) = -0.072 (0.01)	P= 4.5E-9<br>P= 2.6E-6<br>P= 1.3E-26<br><br>P= 1.7E-4<br>P= 0.0045<br>P= 4.3E-7	8/8	GLR<br>Analyses performed under additive model with adjusting for covariates.<br>Conditional analyses:<br>including all variants, which were in the same locus, in one multiple regression model.

Footnotes
Effect directions refer to minor allele T (A on reverse DNA strand); reference genotype: GG (Wt) (CC on reverse DNA strand)
β = variation in serum 25(OH)D per risk-allele.
† Statistical analyses in these studies were performed on log-transformed 25(OH)D values.
[1] Both studies by Nissen et al. were conducted in the same cohort.
[2] Mean values & corresponding P-values derive from univariate analysis in COPD patients.
[3] association not significant for low sun exposure (winter)
Abbreviations: Wt, wild type; VDD, Vitamin D deficiency; Coef., coefficient; GLR, general linear regression; FDR, False discovery rate

Vitamin D	rs12512631 x rs2282679 x rs3755967 x rs4588	Barry et al., 2014 †	x											Caucasian			Ref.: CTCC<br>Estimated diff. in baseline 25(OH)D level for TTCC: -9.34% 95%CI: -11.63, -6.99	P < 0.0001 (Wald test)	7/8	
Vitamin D	rs7041 x rs4588 x rs1155563 x rs2282679	Lu et al., 2011 †	x											Asian (Chinese)			Ref.: GCTA<br>TACC:<br>Beijing: β (SE)= -0.098 (0.02)<br>Shanghai: β (SE)= -0.071 (0.02)<br>Meta-analysis: β (SE)= -0.085 (0.01)	Beijing<br>P = 4.8E-6<br>Shanghai<br>P = 2.4E-4<br>Meta-analysis<br>P = 2.3E-9	8/8	Analyses performed under additive model with adjusting for covariates.<br><br>Meta-analysis: fixed-effect model

Footnotes
β = variation in serum 25(OH)D per haplotype, compared to reference haplotype.
† Statistical analyses in these studies were performed on log-transformed 25(OH)D values.
Abbreviations: Ref., Reference haplotype

Vitamin D	**GC**<br>rs222020	Xu et al., 2014	x											Asian (Chinese)			Pearson correlation<br>r = 0.080	P = 0.001	8/8	
Vitamin D	**GC**<br>rs222020	Barry et al., 2014 †	x											Caucasian			Estimated diff. in 25(OH)D level per variant allele: 3.21%<br>95% CI: 0.24, 6.26	P = 0.03 (Wald test)	7/8	
Vitamin D	**GC**<br>rs222020	Bu et al., 2010	x											Caucasian			Discovery cohort<br>β = 8.42<br>Replication cohort<br>β = 4.42<br>Pooled<br>β = 5.79	Discovery cohort<br>P (Wald test) = 0.010<br>P = 0.009 *<br>P = NS **<br>Replication cohort<br>P (Wald test) = 0.037<br>P = 0.038 *<br>P = NS **<br>Pooled<br>P (Wald test) = 0.001<br>P = 0.002 *<br>P = 0.006 **	7/8	* empirical P-value<br>** adjusted for multiple testing

Footnotes
Pearson correlation coefficient r indicates direction & strength of linear correlation. Value 0-1 indicates a positive correlation. In this case, 25(OH)D is positively (but weakly) associated with the GC SNP.
β = variation in serum 25(OH)D per risk-allele.
† Statistical analyses in this study were performed on log-transformed 25(OH)D values.

Vitamin D	rs222020 x rs2298849	Xu et al., 2014	x											Asian (Chinese)		GLR<br>β (SE) = 0.104 (0.030)	Pearson correlation<br>TA : r = - 0.082<br>CG: r = 0.094	GLR: P = 0.001<br><br>TA: P = 0.001<br>CG: P < 0.001	8/8	
Vitamin D	rs222020 x rs16847015 [1] x rs1155563	Barry et al., 2014 †	x											Caucasian			Ref.: TGT<br>Estimated diff. in baseline 25(OH)D level for TGC: -8.44% 95%CI: -10.55, -6.28	P < 0.0001	7/8	

Footnotes
Pearson correlation coefficient r indicates direction & strength of linear correlation. Value 0-1 indicates a positive correlation. In this case, 25(OH)D is negatively (but weakly) associated with the GC SNPs.
† Statistical analyses in this study were performed on log-transformed 25(OH)D values.
[1] Associations of rs16847015 were not replicated in this review.
Abbreviations: Ref., reference haplotype; GLR, general linear regression

Vitamin D	**GC**<br>rs2298849	Xu et al., 2014	x											Asian (Chinese)		GLR<br>β (SE) = 0.105 (0.029)	Pearson correlation<br>r = 0.095	GLR: P < 0.001<br><br>P < 0.001	8/8	
Vitamin D	**GC**<br>rs2298849	Robien et al., 2013 †	x											Asian (Chinese)	*Geometric Mean (95%CI), [nmol/L]*<br><br>TT (Wt): 63.9 (61.6, 66.3)<br>TC: 66.1 (64.0, 68.3)<br>CC: 72.2 (68.2, 76.4)	TT (Wt): 1<br>TC: 1.034<br>CC: 1.130	Variance explained: 2.1%	P for trend = 0.001	7/8	
Vitamin D	**GC**<br>rs2298849	Bu et al., 2010	x											Caucasian			Discovery cohort<br>β = 6.38<br>Discovery + Replication cohort<br>β = 3.59	Discovery cohort<br>P (Wald test) = 0.026<br>P* = 0.026<br>P** = NS<br>Pooled<br>P (Wald test) = 0.021<br>P* = 0.020<br>P** = NS	7/8	* empirical P-value<br>** adjusted for multiple testing

Footnotes
Pearson correlation coefficient r indicates direction & strength of linear correlation. Value 0-1 indicates a positive correlation. In this case, 25(OH)D is positively (but weakly) associated with the GC SNPs.
† Statistical analyses in this study were performed on log-transformed 25(OH)D values.
Abbreviations: Wt, wild type; GLR, general linear regression

	Gene and SNP	Source	Vitamin												Outcome				Risk of bias	Notes
			Vitamin D					Vit. B12	Vit. B9 (Folate)			Vit. B6 (PLP)	Hcy							
			Calcifediol (25(OH)D)	Calcitriol (1.25(OH)2D)	Ergocalciferol (D2)	VDBP	Vit.D deficiency		Red blood cell folate	Folate / Folic acid	Serum / Plasma Folate			Ancestry	Absolute Values	Effect size	Other	P-value	JBI assessment	
Vitamin D	**GC**<br>rs17467825	Rivera-Paredez et al., 2018					x							Amerindian (Hispanic-Caucasian range)			*Odds to be VDD, OR (95%CI)*<br><br>Codominant model<br>AA (Wt): Ref.<br>AG: 1.64 (1.04, 2.60)<br>GG: 3.48 (1.19, 10.20)<br>Additive model: 1.73 (1.19, 2.52)	P = 0.033<br>P = 0.023<br>P = 0.004	8/8	VDD:<br>25(OH)D < 20 ng/mL
		Nissen et al., 2014 †	x											Caucasian	*Geometric Mean (95%CI), [nmol/L]*<br><br>AA (Wt): 73.8 (70.7, 77.0)<br>GA: 70.0 (66.6, 73.6)<br>GG: 63.6 (57.1, 70.8)	AA (Wt): 1<br>GA: 0.949<br>GG: 0.862		P = 0.0015	7/8	
		Elkum et al., 2014	x											Mixed (59% Arab, 41% Asian)	*Mean [ng/mL]*<br><br>Arabs<br>AA (Wt): 14.4<br>AG: 13.4<br>GG: 10.3<br><br>South Asians<br>AA (Wt): 15.8<br>AG: 12.9<br>GG: 13.0	Arabs<br>AA (Wt): 1<br>AG: 0.931<br>GG: 0.715<br><br>South Asians<br>AA (Wt): 1<br>AG: 0.816<br>GG: 0.823		Arabs<br>P = 0.0165<br>South Asians<br>P = 0.0013	6/8	
		Batai et al., 2014 †	x											Caucasian			β = -0.04	P = 0.003	7/8	

Footnotes
Effect directions refer to minor allele G; reference group: AA (Wt)
Pearson correlation coefficient r indicates direction & strength of linear correlation. Value 0-1 indicates a positive correlation. In this case, 25(OH)D is positively (but weakly) associated with the GC SNPs.
β = variation in serum 25(OH)D per risk-allele.
† Statistical analyses in these studies were performed on log-transformed 25(OH)D values.
Abbreviations: Wt, wild type; VDD, Vitamin D deficiency

Vitamin D	**GC**<br>rs3755967	Rivera-Paredez et al., 2018					x							Amerindian (Hispanic-Caucasian range)			*Odds to be VDD, OR (95%CI)*<br><br>Codominant model<br>CC: Ref.<br>CT: 1.61 (1.02, 2.54)<br>TT: 3.48 (1.19, 10.18)<br>Additive model: 1.71 (1.18, 2.49)	P= 0.040<br>P= 0.023<br>P = 0.005	8/8	VDD:<br>25(OH)D < 20 ng/mL
		Elkum et al., 2014	x											Mixed (59% Arab, 41% Asian)	*Mean [ng/mL]*<br><br>Arabs<br>CC (Wt): 14.4<br>CT: 13.3<br>TT: 10.5<br><br>South Asians<br>CC (Wt): 15.8<br>CT: 12.8<br>TT: 13.0	Arabs<br>AA (Wt): 1<br>AG: 0.924<br>GG: 0.729<br><br>South Asians<br>AA (Wt): 1<br>AG: 0.810<br>GG: 0.823		Arabs<br>P = 0.0368<br>South Asians<br>P = 0.0007	6/8	
		Batai et al., 2014 †	x											Caucasian			β = -0.04	P= 0.002	7/8	

Footnotes
Effect directions refer to minor allele T; reference group: CC (Wt)
† Statistical analyses in this study were performed on log-transformed 25(OH)D values.
Abbreviations: Wt, wild type; VDD, Vitamin D deficiency; Ref., reference

Vitamin D	**GC**<br>rs12512631	Nissen et al., 2014 †	x											Caucasian	*Geometric Mean (95% CI), [nmol/L]*<br><br>TT (Wt): 66.8 (71.3, 78.0)<br>TC: 74.6 (71.3, 78.0)<br>CC: 75.3 (69.0, 82.1)	TT (Wt): 1<br>TC: 1.117<br>CC: 1.127		P = 0.0004	7/8	
		Barry et al., 2014 †	x											Caucasian			Estimated diff. In serum level per variant allele: -4.69%<br>95%CI: 2.50, 6.92	P < 0.0001 (Wald test)	7/8	
		Rivera-Paredez et al., 2018	x											Amerindian (Hispanic-Caucasian range)			"SNP rs12512631 had an inverse association with VD deficiency, only with the unadjusted model."	P = 0.03	8/8	

Footnotes
Effect directions refer to minor allele C; reference group: TT (Wt)
rs12512631 has also been observed to affect Vitamin D in association with other GC SNPs (view Haplotype: rs12512631 x rs2282679 x rs3755967 x rs4588)
† Statistical analyses in these studies were performed on log-transformed 25(OH)D values.
Abbreviations: Wt, wild ; VDD, Vitamin D deficient

Vitamin D	**GC**<br>rs2298850	Elkum et al., 2014	x											Mixed (59% Arab, 41% Asian)	*Mean [ng/mL]*<br><br>Arabs<br>GG (Wt): 14.4<br>GC: 13.3<br>CC: 10.3<br><br>South Asians<br>GG (Wt): 15.5<br>GC: 13.2<br>CC: 13.0	Arabs<br>GG (Wt): 1<br>GC: 0.924<br>CC: 0.715<br><br>South Asians<br>GG (Wt): 1<br>GC: 0.852<br>CC: 0.839		Arabs<br>P = 0.0374<br>South Asians<br>P = 0.0103	6/8	
		Rivera-Paredez et al., 2018					x							Amerindian (Hispanic-Caucasian range)			*Odds to be VDD, OR (95%CI)*<br><br>Codominant model<br>GG (Wt): Ref.<br>GC: 1.64 (1.04, 2.60)<br>CC: 2.88 (0.98, 8.51)<br>Additive model: 1.67 (1.14, 2.43)	P= 0.034<br>P= 0.055<br>P = 0.008	8/8	VDD:<br>25(OH)D <20 ng/mL
		Batai et al., 2014 †	x											Caucasian			β = -0.03	P = 0.04	7/8	

Footnotes
Effect directions refer to minor allele C; reference group: GG (Wt), except for Batai et al. (2014), who refer to the G allele as the minor allele (possibly genotyped the reverse DNA strand).
Pearson correlation coefficient r indicates direction & strength of linear correlation. Value 0-1 indicates a positive correlation. In this case, 25(OH)D is positively (but weakly) associated with the GC SNPs.
β = variation in serum 25(OH)D per risk-allele.
† Statistical analyses in this study were performed on log-transformed 25(OH)D values.

	Gene and SNP	Source	Vitamin											Ancestry	Outcome				Risk of bias	Notes
			Vitamin D					Vit. B12	Vit. B9 (Folate)			Vit. B6 (PLP)	Hcy							
			Calcifediol (25(OH)D)	*Calcitriol (1.25(OH)2D)*	*Ergocalciferol (D2)*	*VDBP*	*Vit.D deficiency*		*Red blood cell folate*	*Folate / Folic acid*	*Serum / Plasma Folate*			*Ancestry*	*Absolute Values*	*Effect size*	*Other*	*P-value*	*JBI assessment*	
		Zhang et al., 2013	x											Asian (Chinese)	*Mean [ng/mL]*<br><br>Wt: 19.32<br>Het.: 18.33<br>Hom.: 16.87	Wt: 1<br>Het.: 0.949<br>Hom.: 0.873	β= - 0.1529	P = 0.006206	7/8	* Bonferroni corrected P-value
						x									Mean [mg/mL] [1]<br><br>TT (Wt): 317<br>TG: 308.5<br>GG: 296	TT (Wt): 1<br>TG: 0.973<br>GG: 0.934	"rs2282679 was associated with concentrations of DBP, with the minor allele G related to reduced protein concentrations."			[1] Values estimated based on Fig. 4 in Zhang et al. (2013)
		Santos et al., 2019	x											Caucasian (20% African + Caucasian mixed)	*Mean ± SD [ng/mL]*<br><br>AA (Wt) : 23.39 ± 8.79<br>AC: 22.83 ± 7.80<br>CC: 19.70 ± 7.17	AA (Wt): 1<br>AC: 0.976<br>CC: 0.842	β = -1.3	P = 0.034	7/8	
							x										*Prevalence ratio of VDD for CC*<br><br>PR (95%CI): 1.696 (1.263, 2.276)	P < 0.001		PR for AC not significant<br><br>VDD:<br>25(OH)D < 20 ng/mL
						x									*Mean ± SD [µg/mL]*<br><br>AA (Wt): 203.13 ± 27.90<br>AC: 196.41 ± 30.04<br>CC: 180.88 ± 38.20	AA (Wt): 1<br>AC: 0.967<br>CC: 0.890	β = -9.3	P < 0.001		
		Perna et al., 2013	x											Caucasian	*Median (IQR) [nmol/L]*<br><br>**Women**<br>AA (Wt): 41.1 (31.3; 53.7)<br>AC: 39.3 (30.3; 50.5)<br>CC: 34.7 (29.7; 46.1)<br><br>**Men**<br>AA (Wt): 55.1 (39.2; 73.4)<br>AC: 51.5 (35.8; 69.8)<br>CC: 44.4 (33.9; 62.7)	**Women**<br>AA (Wt): 1<br>AC: 0.956<br>CC: 0.844<br><br>**Men**<br>AA (Wt): 1<br>AC: 0.935<br>CC: 0.806	*Adjusted Estimates (SE) [nmol/L]*<br><br>AA: Ref. 0.0<br>AC: −2.2 (0.7)<br>CC: −5.8 (1.4)	"Linear regression analysis showed strong associations between Vit. D and the genetic variant in the total population and by sex"	7/8	Estimates adjusted for covariates
	**GC**<br>rs2282679	Nissen et al., 2014 [†]	x											Caucasian	*Geometric mean (95%CI), [nmol/L]*<br><br>AA (Wt): 73.8 (70.7, 77.0)<br>CA: 70.1 (66.6, 73.7)<br>CC: 63.6 (57.1, 70.8)	AA (Wt): 1<br>AC: 0.950<br>CC: 0.862		P= 0.0020	7/8	
		Li et al., 2014	x											Asian (Chinese)			β (SE): -3.077 (0.903)	P = 0.0007<br>P = 0.006 *	8/8	* Bonferroni corrected
		Barry et al., 2014 [†]	x											Caucasian			"baseline associations were statistically significant."			Results not shown, authors did not respond to request for aboslute values.
Vitamin D		Lu et al., 2011 [†]	x											Asian (Chinese)			GLR<br>Beijing: β (SE)= -0.080 (0.01)<br>Shanghai: β (SE)= -0.063 (0.01)<br>Meta-analysis: β (SE)= -0.072 (0.01)<br><br>Conditional analyses<br>Beijing: β (SE)= -0.069 (0.02)<br>Shanghai: β (SE)=-0 .052 (0.02)<br>Meta-analysis: β (SE)= -0.061 (0.01)	P = 7.4E-8<br>P = 4.1E-6<br>P = 4.9E-24<br><br>P = 0.0022<br>P = 0.0080<br>P = 1.9E-5	8/8	GLR<br>Analyses performed under additive model with adjusting for covariates.<br><br>Conditional analyses:<br>incl. all variants, which were in the same locus, in one multiple regression model.
		Wang et al., 2010 [†]	x											Caucasian	*Mean ± SE [nmol/L]*<br><br>Wt: 82.6 ± 0.73<br>het.: 74.8 ± 0.81<br>hom.: 64.6 ± 1.79	Wt: 1<br>het.: 0.906<br>hom.: 0.782		*Candidate gene analysis*<br>Discovery Cohort<br>P = 4.57E-63<br>Replication Cohort<br>P = 2.88E-48<br>Overall<br>P = 2.9E-108	8/8	All Analyses reached genome-wide significance
							x										*Odds for low Vit. D, OR (95%CI)*<br><br>< 75nmol/L: 1.63 (1.53, 1.73)<br>< 50nmol/L: 1.49 (1.40, 1.59)	P = 3.5E-50<br>P = 7.5E-33		OR per copy of the risk allele
	**GC**<br>rs2282679					x											"minor allele related to lower DBP concentration"	P = 4.0E-42		"rs2282679 was strongly associated with DBP, with the minor allele related to lower DBP conc."
		Batai et al., 2014 [†]	x											Caucasian			β = -0.05	P = 0.001	7/8	
		Elkum et al., 2014	x											Mixed (59% Arab, 41% Asian)	*Mean [ng/mL]*<br><br>Arabs<br>TT (Wt): 14.4<br>GT: 13.3<br>GG: 10.4<br>South Asians<br>TT (Wt): 15.8<br>GT: 12.8<br>GG:13.0	Arabs<br>AA (Wt): 1<br>AG: 0.924<br>GG: 0.722<br><br>South Asians<br>AA (Wt): 1<br>AG: 0.810<br>GG: 0.823		Arabs<br>P = 0.0377<br>South Asians<br>P = 0.0007	6/8	
		Rivera-Paredez et al., 2018					x							Amerindian (Hispanic-Caucasian range)			*Odds to be VDD, OR (95%CI)*<br><br>Subsample (n=400)<br>1.73 (1.19, 2.52)<br><br>Whole sample (n= 689)<br>1..53 (1.15, 2.04)	P = 0.004<br><br>P= 0.003	8/8	VDD:<br>25(OH)D < 20 ng/mL
	**GC**<br>rs2282679	Ahn et al., 2010 [1]	X											Caucasian	*Mean [nmol/L]*<br><br>ATBC<br>AA (Wt): 42.1<br>AC: 37.2<br>CC: 35.2<br><br>CLUE II<br>AA (Wt): 67.5<br>AC: 58.4<br>CC: 44.3<br><br>PLCO<br>AA (Wt): 61.5<br>AC: 55.3<br>CC: 53.4<br><br>NHS-CGEMS<br>AA (Wt): 86.8<br>AC: 77.3<br>CC: 72.5<br><br>NHS-T2D<br>AA (Wt): 58.8<br>AC: 55.8<br>CC: 48.8	ATBC<br>AA (Wt): 1<br>AC: 0.884<br>CC: 0.836<br><br>CLUE II<br>AA (Wt): 1<br>AC: 0.865<br>CC: 0.656<br><br>PLCO<br>AA (Wt): 1<br>AC: 0.899<br>CC: 0.868<br><br>NHS-CGEMS<br>AA (Wt): 1<br>AC: 0.891<br>CC: 0.835<br><br>NHS-T2D<br>AA (Wt): 1<br>AC: 0.949<br>CC: 0.830	ATBC<br>β(SE): -0.36 (0.05)<br>CLUE II<br>β(SE): -0.69 (0.22)<br>PLCO<br>β(SE): -0.37 (0.05)<br>NHS-CGEMS<br>β(SE): -0.46 (0.09)<br>NHS-T2D<br>β(SE): -0.24 (0.07)	ATBC<br>P= 1.5E-8<br>CLUE II<br>P= 2.2E-3<br>PLCO<br>P= 5.8E-13<br>NHS-CGEMS<br>P= 1.2E-7<br>NHS-T2D<br>P= 9.5E-4	6/8	

	Gene and SNP	Source	Vitamin												Outcome				Risk of bias	Notes
			Vitamin D					Vit. B12	Vit. B9 (Folate)			Vit. B6 (PLP)	Hcy							
			Calcifediol (25(OH)D)	*Calcitriol (1.25(OH)2D)*	*Ergocalciferol (D2)*	*VDBP*	*Vit.D deficiency*		*Red blood cell folate*	*Folate / Folic acid*	*Serum / Plasma Folate*			*Ancestry*	*Absolute Values*	*Effect size*	*Other*	*P-value*	*JBI assessment*	
Vitamin D		Slow et al., 2020	x											Caucasian			*Baseline 25(OH)D [nmol/L]*<br>β (95% CI): -6.34 (-10.29, -2.39) [2,3]	P = 0.00181	12/13	[2] association remained significant after 2 mo of Vit. D3 supplementation and after Bonferroni correction at that time point<br>[3] association remained significant after 18 mo of Vit. D3 supplementation
						x											*Baseline VDBP [nmol/L]*<br>β (95% CI): -298.81 (-440.35, -157.26)	P = 0.00005 *		*remained signif. after Bonferroni correction
		Kwak et al., 2018	x											Asian (Korean)			"found negative associations btw serum 25(OH)D level and the effect allele G"	P = 0.004	7/8	
		Man et al., 2022	x											Asian (Chinese)			*Odds to be VDD, OR (95%CI):*<br>Genotypic model<br>AC: 2.30 (1.37, 3.87)<br>Univariate model<br>1.52 (1.03, 2.24)<br>Multivariate model<br>1.57 (1.04, 2.39)	P < 0.01 *<br>P = 0.04<br>P = 0.03	7/8	VDD:<br>25(OH)D < 30 ng/ mL<br>*remained signif. after Bonferroni correction
	**GC**<br>rs2282679	Waterhouse et al., 2014	x											Caucasian	*Mean change (95%CI) [nmol/L]*<br>AA (Wt): 43.3 (41.9, 44.8)<br>AC: 41.0 (39.3, 42.7)<br>CC: 36.0 (32.3, 39.7)	AA (Wt): 1<br>AC: 0.947<br>CC: 0.831		P < 0.005<br>(P trend < 0.005)	12/13	significant after Bonferroni correction (P trend = 2E-4)
		Cheung et al., 2013 †	x											Asian (Chinese)			β (95%CI): -0.066 (-0.100, 0.033)<br>**SNP * season**<br>Summer/Autumn<br>β (95%CI): -0.063 (-0.105, -0.021)<br>Winter/Spring<br>β (95%CI): -0.072 (-0.126, -0.019)	P= 9.7E-5<br>**SNP * season**<br>Summer/Autumn<br>P= 0.003<br>Winter/Spring<br>P= 0.008	7/8	
							x										*Odds to be VDD, OR (95%CI):*<br>1.51 (1.19, 1.93)<br>Independent association:*<br>OR (95%CI): 1.52 (1.19, 1.94)<br>**SNP * season**<br>Summer/Autumn<br>OR (95%CI): 1.64 (1.19, 2.27)	P= 8.6E-4<br>P= 8.1E-4<br>**SNP * season**<br>Summer/Autumn<br>P= 0.003		* model adjusted for rs12785879<br>Association for Winter/Spring was not significant<br>VDD:<br>25(OH)D < 20 ng/mL
		Didriksen et al., 2013	x											Caucasian	*Mean ± SD [nmol/L]*<br>TT (Wt): 61.9 ± 24.5<br>TG: 53.4 ± 20.3<br>GG: 46.5 ± 16.4	TT (Wt): 1<br>TG: 0.863<br>GG: 0.751	*Increase (delta) in serum 25(OH)D after Vit.D supplementation*<br>GG baseline: 46.0 ± 15.5<br>GG 6mo: 139.2 ± 23.9<br>MD: 93.2 ± 21.6	GG: P < 0.01<br>Delta: P < 0.05	7/8	TG not significant
	**G**C<br>rs2282679	Trummer et al., 2012	x											Caucasian	*Mean ± SD [ngl/mL]*<br>Cross-sectional study cohort<br>TT (Wt): 35.9 ± 13.3<br>TG: 31.8 ± 12.9<br>GG: 26.1 ± 8.7<br>Prospective cohort study<br>TT (Wt): 9.4 ± 7.6<br>TG: 8.7 ± 5.5<br>GG: 7.5 ± 4.6	Cross-sectional<br>TT (Wt) : 1<br>TG: 0.886<br>GG: 0.727<br>Prospective cohort<br>TT (Wt): 1<br>TG: 0.926<br>GG: 0.798		P= 0.001<br>P= 0.048	7/8	94% of subjects in the prospective cohort were Vit. D deficient (thus the low mean values)
		Slater et al., 2017					x							Caucasian			*Odds to be VDD for T allele, OR (95%CI)*<br>Initial model<br>0.42 (0.18, 0.93)<br>Final model:<br>0.346 (0.143, 0.838)	P= 0.037<br>P= 0.019	7/8	Initial model: all potential confounders final model: significant confounders only<br>VDD:<br>25(OH)D < 30ng/mL
		Simon et al., 2011	x											Caucasian			"Each additional 'C' allele was associated with a 7.8 nmol/L decrease in 25(OH)D"		5/8	
		Thongthai et al., 2015	x											Asian (Thai)	*Mean ± SD [ng/mL]*<br>AA (Wt): 30.1 ± 9.0<br>AC: 27.5 ± 8.7<br>CC: 25.2 ± 8.3	AA (Wt): 1<br>AC: 0.914<br>CC: 0.837	*Multivariate analysis*<br>Association C allele: β= -0.17	P < 0.001<br>Multivariate P<0.001	8/8	
							x										*"A allele: 34% lower risk of VDD"*<br>*Odds to be VDD for each C allele*<br>OR (95%CI): 1.80 (1.57, 2.01)<br>*VDD prevalence by genotype*<br>AA (Wt): 10.6%<br>AC: 16.7%<br>CC: 26.4%	P < 0.001		VDD:<br>25(OH)D < 20ng/mL

Footnotes

Unless specified, effect directions refer to minor allele C (G on reverse strand); reference group: AA (Wt) (T on reverse strand)

β = variation in serum 25(OH)D per risk allele.

† Statistical analyses in these studies were performed on log-transformed 25(OH)D values.

[1] Ahn et al (2020) analyzed 5 GWAS for associations between SNPs and 25(OH)D, the cohorts listed here found significant results. Subjects include cases (VDD) and contros.

Abbreviations: Wt, wild type; VDD, Vitamin D deficiency; PR, prevalence ratio; Ref., reference; GLR, general linear regression; MD, mean difference; VDBP, Vitamin D binding protein

	Gene and SNP	Source	Vitamin												Outcome				Risk of bias	Notes
			Vitamin D					Vit. B12	Vit. B9 (Folate)			Vit. B6 (PLP)	Hcy							
Vitamin D			Calcifediol (25(OH)D)	Calcitriol (1,25(OH)2D)	Ergocalciferol (D2)	VDBP	Vit.D deficiency		Red blood cell folate	Folate / Folic acid	Serum / Plasma Folate			Ancestry	Absolute Values	Effect size	Other	P-value	JBI assessment	
	rs705117 x rs2282679 x rs1491710	Zhang et al., 2013	x											Asian (Chinese)			TGA<br>β = - 0.1529<br>freq.: 0.3083<br>CTA<br>β = 0.1<br>freq.: 0.1758	P = 6.6E-5<br><br>P = 0.03234	7/8	
Vitamin D	GC<br>rs1155563	Perna et al., 2013	x											Caucasian	*Median (IQR) [nmol/L]*<br><br>**Women**<br>TT (Wt): 41.0 (31.3; 53.7)<br>TC: 39.1 (30.5; 50.0)<br>CC: 34.5 (29.5; 46.1)<br><br>**Men**<br>TT (Wt): 54.6 (38.4; 73.2)<br>TC: 51.5 (36.8; 69.8)<br>CC: 45.6 (33.9; 63.9)	**Women**<br>TT (Wt): 1<br>TC: 0.954<br>CC: 0.841<br><br>**Men**<br>TT (Wt): 1<br>TC: 0.943<br>CC: 0.835	*Adjusted Estimates (SE) [nmol/L]*<br><br>TT: Ref. 0.0<br>TC: -2.1 (0.7)<br>CC: -5.8 (1.4)	N/R *	7/8	* "Linear regression analysis showed strong associations between Vit. D and the genetic variant in the total population and by sex"
		Barry et al., 2014 †	x											Caucasian			Estimated diff. In serum level per variant allele: -8.44%<br>95%CI: -10.48, -6.35	P < 0.0001 (Wald test)	7/8	
		Batai et al., 2014 †	x											Black African			African American: β= -0.04	P = 0.048	7/8	
		Elkum et al., 2014	x											Arab	*Mean [ng/mL]*<br><br>TT (Wt): 14.4<br>TC: 13.3<br>CC: 10.4	TT (Wt): 1<br>TC: 0.924<br>CC: 0.722		P = 0.0289	6/8	
		Rivera-Paredez et al., 2018					x							Amerindian (Hispanic-Caucasian range)			*Odds to be VDD, OR (95%CI)*<br><br>Codominant model<br>TT: Ref.<br>TC: 1.76 (1.13, 2.76)<br>CC: 2.96 (0.71, 12.34)<br>Additive model: 1.76 (1.18, 2.61)	P= 0.013<br>P= 0.135<br>P = 0.006	8/8	VDD<br>25(OH)D <20 ng/mL
		Slow et al., 2020	x											Caucasian			*Baseline 25(OH)D [nmol/L]*<br>β (95% CI): -5.48 (-9.37, -1.59) [2]	P = 0.00609	12/13	[2] association remained significant after 2 mo of Vit. D3 supplementation and after Bonferroni correction at that time point<br><br>* Remained significant after Bonferroni correction
						x											*Baseline VDBP [nmol/L]*<br>β (95% CI): -257.38 (-397.16, -117.59)	P= 0.00036 *		
		Lu et al., 2011 †	x											Asian (Chinese)			GLR<br>Beijing: β (SE)= -0.048 (0.01)<br>Shanghai: β (SE)= -0.042 (0.01)<br>Meta-analysis: β (SE)= -0.045 (0.01)	P= 9.8E-4<br>P= 0.0015<br>P= 2.0E-10	8/8	Analyses performed under additive model, adjusted for covariates.<br>Meta-analysis: fixed-effect model

Footnotes
Effect directions refer to minor allele C (G on reverse strand) ; reference group: AA (Wt) (T on reverse strand)
β = variation in serum 25(OH)D per haplotype, compared to reference haplotype. Lu et al (2011) refer to changes in log-25(OH)D
† Statistical analyses in these studies were performed on log-transformed 25(OH)D values.
[1] Ahn et al (2020) analyzed 5 GWAS for associations between SNPs and 25(OH)D, the cohorts listed here found significant results. Subjects include cases and contros.
[illegible]

Vitamin D		Sinotte et al., 2009	x											Caucasian	*Mean ± SE [nmol/L]*<br><br>GG: 67.5 ± 1.1<br>GT: 64.5 ± 0.8<br>TT: 60.8 ± 1.5<br><br>Seasonal Association<br>**May-Oct:**<br>GG: 73.4 ± 1.4<br>GT: 71.4 ± 1.1<br>TT: 64.8 ± 2.1	GG: 1<br>GT: 0.956<br>TT: 0.901<br><br>**May-Oct**<br>GG: 1<br>GT: 0.973<br>TT: 0.883	**Total**<br>β ± SE = 23.29 ± 0.89<br>**May-Oct**<br>β = -3.78	GG: Ref.<br>GT: P = 0.034<br>TT: P = 0.0003<br><br>Total<br>P for trend = 0.0003<br>Summer<br>P for trend = 0.0018	7/8	Association not significant for Winter
		Santos et al., 2019	x											Caucasian (20% African + Caucasian mixed)	*Mean ± SD [ng/mL]*<br><br>TT (Wt) : 21.48 ± 7.54<br>TG: 23.20 ± 8.24<br>GG: 23.78 ± 9.14	GG: 1<br>TG: 0.976<br>TT: 0.903	β = 1.2	P= 0.030	7/8	
		Robien et al., 2013 †	x											Asian (Chinese)	*Geometric Mean (95%CI) [nmol/L]*<br><br>TT (Wt): 64.0 (61.9, 66.1)<br>TG: 67.7 (65.4, 70.0)<br>GG: 70.6 (65.9, 75.6)	GG: 1<br>TG: 0.959<br>TT: 0.907	Variance explained: 1.6%	P for trend = 0.003	7/8	
	GC<br>rs7041	Rivera-Paredez et al., 2020					x							Hispanic			"Carriers of the GG & GT had lower prevalence of VDD compared to TT genotype"	GG: P = 0.019<br>GT: P = 0.022	8/8	VDD:<br>25(OH)D <20 ng/mL
		Pooyan et al., 2020		x										Iranian			"T allele carriers had lower Vitamin D compared to others"<br><br>β ± SE = -0.46 ± 0.14<br>OR: 0.63	P < 0.0001<br>(P-trend: 0.002)	8/8	
		Meshkibaf et al., 2021					x							Iranian (Persian)			**Allele freq.(n)**<br>**VDD**<br>GG: 35.4% (17)<br>GT: 31.3% (15)<br>TT: 33% (n= 16) *<br>**Insufficient**<br>GG:34.2% (40)<br>GT: 52.1% (61) **<br>TT: 13.7% (n=16)<br>**Normal**<br>GG: 41.9% (13)<br>GT: 35.5% (11)<br>TT: 22.6% (7)	* P = 0.03<br>** P= 0.02	5/8	VDD:<br>25(OH)D < 20 ng/ml<br>Insufficient:<br>25(OH)D 21–29 ng/ml<br>Normal:<br>25(OH)D > 30 ng/ml<br><br>P-values after Bonferroni correction
	GC<br>rs7041	Lafi et al., 2015	x											Mostly Caucasian (Mixed)	*Mean ± SD [ng/mL]*<br><br>GG (Wt): 36.1 ± 20.4<br>GT: 28.5 ± 21.8<br>TT: 24.6 ±17.7<br><br>TT + GT: 27.2 ± 20.5<br>GG + GT: 31.3 ± 21.5	GG (Wt): 1<br>GT: 0.789<br>TT: 0.681<br><br>TT + GT: 1<br>GG + GT: 1.151		GT: P = 0.001<br>TT: P= 0.004<br><br>TT + GT: P = 0.0006<br>GG + GT: P= 0.026	5/8	
							x										*Odds for low Vit. D, OR (95%CI)*<br><br>GG (Wt):Ref.<br>GT: 3.25 (1.59, 6.63)<br>TT: 3.31 (1.46, 7.50)<br><br>TT + GT: 3.27 (1.66, 6.42)<br>GG+ GT: 2.15 (1.10, 4.20)			
		Janssens et al., 2010	x											Caucasian	*Mean ± SD [ng/mL]*<br><br>GG (Wt): 26.3 ± 8.7<br>GT: 24.6 ± 9.1<br>TT: 20.9 ± 5.0	GG (Wt): 1<br>GT: 0.935<br>TT: 0.795	Multivariate analysis<br>β (95%CI): -4.5 (-6.5, -2.4)	P < 0.0001<br><br>GT: P = 0.5 (NS)<br>TT: P = 0.03		

	Gene and SNP	Source	Vitamin												Outcome				Risk of bias	Notes
			Vitamin D					Vit. B12	Vit. B9 (Folate)			Vit. B6 (PLP)	Hcy							
			Calcifediol (25(OH)D)	Calcitriol (1.25(OH)2D)	Ergocalciferol (D2)	VDBP	Vit.D deficiency		Red blood cell folate	Folate / Folic acid	Serum / Plasma Folate			Ancestry	Absolute Values	Effect size	Other	P-value	JBI assessment	
Vitamin D	GC<br>rs7041	Gaffney-Stomberg et al., 2017	x											Caucasians, African Americans, other			**Entire cohort**<br>Discovery cohort<br>β (SE) = 3.201 (0.614)<br>Replication<br>β (SE) = 4.458 (0.667)<br><br>**Caucasians only**<br>Discovery cohort<br>β (SE) = -2.179 (0.696)<br>Replication<br>β (SE) = -3.268 (0.863)	**Entire cohort**<br>P = 2.88E-7<br>P* = 1.97E–10<br>P ** = 5.32E–09<br>**Caucasians**<br>P = 0.0019<br>P* = 0.0002<br>P** = 0.0053	7/8	*FDR corrected<br>** Bonferroni corrected
				x													**Entire cohort**<br>Discovery cohort<br>β (SE) = 4.127 (1.665)<br>Replication<br>β (SE) = 9.633 (2.504)<br><br>**African Americans only**<br>Discovery cohort<br>β (SE) = -9.705 (4.067)<br>Replication: NS	**Entire cohort**<br>P = 0.0136<br>P* = 0.04701<br>P ** = 0.0039<br>**African Americans**<br>P = 0.0198		
		Engelman et al., 2008	x											Hispanic & African American			Effect of SNP (T allele), *Coef ± SE*<br><br>San Antonio Hispanics<br>-0.181 ± 0.061<br>San Luis Hispanics<br>-0.220 ± 0.063<br>Los Angeles African Americans<br>-0.2159 ± 0.071	P = 0.003<br><br>P < 0.001<br><br>P = 0.025	6/8	Association between rs7041 and 1.25(OH)2D was not significant
		Barry et al., 2014 †	x											Caucasian			Estimated diff. In serum level per variant allele: -6.69%<br>95%CI: -8.57, -4.76	P < 0.0001 (Wald test)	7/8	
		Engelman et al., 2012	x											Caucasian	*Association stratified by season [1] [nmol/L]*<br><br>High exposure<br>GG (Wt): 67.8<br>GT: 63.1<br>TT: 57.4	GG Wt): 1<br>GT: 0.931<br>TT: 0.847	Additive genetic model<br>β = -0.1<br><br>SNP * high exposure<br>β = -0.33	P = 0.002<br><br>SNP * season<br>P = 0.01<br>SNP * exposure<br>P < 0.0001	7/8	
		Szili et al., 2018	x											Caucasian	*Mean [ng/mL]*<br><br>AA (Wt): 13.6<br>AC: 14.6<br>CC: 16.2	CC: 1<br>CA: 0.901<br>AA: 0.840	Explained variance: 2.6%<br><br>MLR<br>dependent: β (SE): −2.959 (0.905)<br>univariate: β (SE):−1.228 (0.718)	CC: P = 0.006 *<br><br>MLR<br>P = 0.005	7/8	* remained signif. After FDR correction<br><br>Association was not significant after Benjamini-Hochberg correction<br><br>A allele noted as ref. Allele in this study
	GC<br>rs7041	Wang et al., 2010 †	x											Caucasian			after adjusting for rs7041, no other SNP in GC remained significant in this cohort.	*Candidate gene analysis*<br>Discovery Cohort<br>P= 3.74E-42<br>Replication Cohort<br>P= 1.78E-18<br>Overall<br>P= 6.31E-59	8/8	All analyses reached genome-wide significance
		Batai et al., 2014 †	x											Caucasian			β= -0.04	P= 0.0007	7/8	after adjusting for rs7041, no other SNP in GC remained significant in this cohort.
		Elkum et al., 2014	x											Mixed (59% Arab, 41% Asian)	*Mean [ng/mL]*<br><br>Arabs<br>CC (Wt): 14.5<br>AC: 14.3<br>AA: 11.7<br><br>South Asians<br>CC (Wt): 15.8<br>AC: 14.4<br>AA: 12.3	Arabs<br>CC (Wt): 1<br>AC: 0.986<br>AA: 0.807<br><br>South Asians<br>CC (Wt): 1<br>AC: 0.911<br>AA: 0.778		Arabs<br>P = 0.0110<br>South Asians<br>P = 0.0072	6/8	
		Slow et al., 2020	x											Caucasian			*Baseline 25(OH)D [nmol/L]*<br>β (95% CI): -7.40 (-10.90, -3.89) [2]<br><br>*Baseline free 25(OH)D [pmol/L]*<br>β (95% CI): -2.45 (-4.36, -0.54) [2]	P = 0.00005*<br><br>P = 0.01249	12/13	[2] association remained significant after 2 mo of Vit. D3 supplementation<br>*remained signif. after Bonferroni correction
						x											*Baseline VDBP [nmol/L]*<br>β (95% CI): -162.79 (-292.66, -32.92)	P = 0.01461		
	GC<br>rs7041	Lee et al., 2021	x											Asian (Korean)	*Mean ± SE [ng/mL]*<br><br>CC (Wt): 21.0 ± 0.50<br>AC: 19.8 ± 0.22<br>AA: 18.5 ± 0.18	CC: 1<br>CA: 0.0.943<br>AA: 0.881	*Associations in multivariate model*<br><br>β = 1.2090<br>P= 3.20E-9 **	P= 2.44E-8 *	7/8	* obtained through GLM<br>** obtained thorugh additive linear model
		Parlato et al., 2023				x								Black-African			*SCCS Sample**<br>β (SE) = 0.48 µg/mL<br><br>Variance explained: 0.01%<br><br>"C allele was associated with a 1.83µg/L increase of VDBP.<br>CC: 28.12% higher VDBP than AA."	P = 4-1E-21	7/8	Southern Community Cohort Study<br><br>These results refer to the major allele C
		Didriksen et al., 2013	x											Caucasian	*Mean ± SD [nmol/L]*<br><br>Wt: 63.7 ± 24.8<br>Het.: 57.2 ± 22.4<br>Hom.: 50.0 ± 19.2	Wt: 1<br>Het: 0.898<br>Hom: 0.785		Hom.: P < 0.01	7/8	Difference for heterozygotes was not significant
		Simon et al., 2011	x											Caucasian			"Each additional 'T' allele was associated with a 5.5 nmol/L decrease in 25(OH)D"		5/8	
		Hansen et al., 2015	x											African-American	*Mean in supplement users [mg/L]*<br><br>GG/GT: 29<br>TT: 25.3	GG/GT: 1<br>TT: 0.872	β ± SE = -0.93 ± 0.53	β: P = 0.08<br><br>*Mean values*<br>P = 0.002	7/8	The association was not significant in non-supplement users (Vit.D & multivitamin)
		Larcombe et al., 2012				x								Canadian First Nation (Dené)	*Mean ± SD [µg/mL]*<br><br>GG (Wt): 469.4 ± 72.85<br>GT: 277.26 ± 6.40<br>TT: 208.1 ± 153.8	GG (Wt): 1<br>GT: 0.591<br>TT: 0.443		P < 0.005		Associations shown apply to winter-time, those for summer were not significant

Footnotes

Effect directions refer to risk allele T (A on reverse DNA strand); reference genotype: GG (CC on reverse DNA strand) - Exception: Parlato et al., 2023 refer effect size to major allele C

β = variation in serum 25(OH)D per risk-allele.

† Statistical analyses in these studies were performed on log-transformed 25(OH)D values.

[1] association not significant for low sun exposure (winter)

Abbreviations: Wt, wild type; VDD, Vitamin D deficiency; VDBP, Vitamin D binding protein; GLR, general linear regression; FDR, False discovery rate; Coef., coefficient

	Gene and SNP	Source	Vitamin												Outcome				Risk of bias	Notes
			Vitamin D					Vit. B12	Vit. B9 (Folate)			Vit. B6 (PLP)	Hcy							
			Calcifediol (25(OH)D)	Calcitriol (1,25(OH)2D)	Ergocalciferol (D2)	VDBP	Vit.D deficiency		Red blood cell folate	Folate / Folic acid	Serum / Plasma Folate			Ancestry	Absolute Values	Effect size	Other	P-value	JBI assessment	
Vitamin D		Fohner et al., 2016	x											Alaskan Native (Yup'ik)			Variance explained by Gc1s haplotype: 2.8%	Gc2: P = 1.4E-4<br>Gc1f: NS<br>Gcx (TC): NS	6/8	The p-values signify the associations with 25(OH)D3 in comparison with the reference haplotype GC
		Li et al., 2014	x											Asian (Chinese)	*Mean ± SE [nmol/L]*<br><br>Gc2-2(Ref.): 51.9 ± 1.9<br>Gc1s-1s: 62.0 ± 1.9<br>Gc1s-1f: 59.9 ± 1.2<br>Gc2-1s: 58.6 ± 1.5	Gc2-2(Ref.): 1<br>Gc1s-1s: 1.195<br>Gc1s-1f: 1.154<br>Gc2-1s: 1.129		All P < 0.05	8/8	
	**GC**<br>rs4588 x rs7041<br>Haplotypes	Gozdzik et al., 2011 †	x											Diverse (East & South Asian, Europan, other)	*Mean ± SD [nmol/L]*<br><br>Fall<br>Gc1f-1f (Ref.): 55.5 ± 18.2<br>GC2-1f: 43.4 ± 12.7<br>GC2-1s: 41.2 ± 11.2<br>GC2-2: 34.2 ± 6.53<br><br>Winter<br>Gc1f-1f (Ref.): 40.8 ± 18.9<br>GC2-1f: 26.6 ± 12.0<br>GC2-2: 24.4 ± 7.39	Fall<br>Gc1f-1f (Ref.): 1<br>GC2-1f: 0.782<br>GC2-1s: 0.742<br>GC2-2: 0.616<br><br>Winter<br>Gc1f-1f (Ref.): 1<br>GC2-1f : 0.652<br>GC2-2: 0.598	*Partial correlations*<br><br>Fall<br>GC2-1f: -0.256<br>GC2-1s: -0.240<br>GC2-2: -0.303<br>Winter<br>GC2-1f : -0.311<br>GC2-2:-0.291<br><br>*Variance explained by Gc2-2: 7%*	Fall<br>P=0.014<br>P=0.021<br>P=0.003<br>Winter<br>P= 0.005<br>P= 0.009	7/8	
		Robien et al., 2013 †	x											Asian (Chinese)	*Geometric Mean (95%CI) [nmol/L]*<br><br>Gc2-2 (Ref.): 56.6 (52.1, 61.4)<br>Gc1s-1s: 70.9 (66.1, 76.2)<br>Gc1s-1f: 67.6 (64.7, 70.6)<br>Gc2-1s: 67.3 (63.3, 71.5)<br>GC1f-1f: 68.9 (65.1, 72.8)<br>GC2-1f: 62.3 (59.3, 65.5)	Gc2-2 (Ref.): 1<br>Gc1s-1s: 1.253<br>Gc1s-1f: 1.194<br>Gc2-1s: 1.189<br>GC1f-1f: 1.217<br>GC2-1f: 1.101		All haplotypes were significantly different from Gc2-2 at P < 0.01<br><br>P for trend = 0.0001	7/8	
			x												*Mean ± SD [ng/mL]*<br><br>GC1s-1s (Ref.): 23.83 ± 9.19<br>GC1f-1f: 20.63 ± 8.29<br>GC2-2:20.25 ± 7.39	GC1s-1s (Ref.): 1<br>GC1f-1f: 0.866<br>GC2-2: 0.850	β = -1.9	P = 0.015		PR's adjusted for age, BMI, & vitamin D supplementation. Associations were also significant in the unadjusted model (view source)
		Santos et al., 2019				x								Caucasian (20% African + Caucasian mixed)	*Mean ± SD [µg/mL]*<br><br>GC1s-1s (Ref.): 199.41 ± 29.89<br>GC1f-1f: 204.83 ± 31.58<br>GC2-2: 180.75 ± 37.58	GC1s-1s (Ref.): 1<br>GC1f-1f: 1.027<br>GC2-2: 0.906	β = -7.7	P = 0.012	7/8	
	**GC**<br>rs4588 x rs7041<br>Haplotypes						x										*GC2 frequency by Vit.D status*<br><br>VDD: 28.4%<br>Sufficient: 15.0%<br><br>*Prevalence Ratio for VDD,* PR (95%CI)<br>GC1s-1s: Ref.<br>GC1f-1f: 1.593 (1.120, 2.265)<br>GC2-2: 0.881 (0.790, 0.982)	P = 0.016<br><br>P = 0.010<br>P = 0.022		VDD:<br>25(OH)D < 20ng/mL<br><br>PR's adjusted for age, BMI, & vitamin D supplementation. Associations were also significant in the unadjusted model (view source)
		Rivera-Paredez et al., 2020					x							Hispanic			Odds for VDD, *OR (95%CI)*<br><br>GC1fs-1f: Ref.<br>GC2-2: 2.83 (1.14, 7.02)<br>GC2-1f: 2.30 (1.40, 3.78)	N/R	8/8	VDD:<br>25(OH)D < 20ng/mL
		Pooyan et al., 2020					x							Iranian			*Frequencies of VDD by haplotype*<br><br>**Gc1s (GC)**<br>VDD: 76.5% <> Normal: 23.5%<br>**Gcx (TC)**<br>VDD: 66.7% <> Normal: 33.3%<br>**Gc2 (TA)**<br>VDD: 79.3% <> Normal: 20.7%<br>**Gc1f (GA)**<br>VDD: 78.7% <> Normal: 21.3%	N/R	8/8	VDD:<br>25(OH)D < 30 ng/ mL
		Meshkibaf et al., 2021					x							Iranian (Persian)			*Frequencies of haplotype by VDD*<br><br>**Gc1f (GA)**<br>VDD: 42.7% (41/96) *<br>Insufficient: 20.5% (48/234)<br>Normal: 33.9% (21/62)<br><br>**Gc2 (TA)**<br>VDD: 6.4% (6/97)<br>Insufficient: 19.3% (45/234) **<br>Normal: 6.5% (4/62)	P < 0.001*<br>P = 0.001 **	5/8	VDD:<br>25(OH)D <20 ng/ml<br>Insufficiency:<br>25(OH)D 21–29 ng/ml<br>Normal:<br>25(OH)D >30 ng/ml<br><br>P-values remained significant after Bonferroni correction
	**GC**<br>rs4588 x rs7041<br>Haplotypes	Lafi et al., 2015					x							Mostly Caucasian (Mixed)			**Gc1s-1f (n= 56)**<br>VDD: 46.5% (26)<br>Insuff.: 8.9% (5)<br>Suff.: 44.6% (25)<br>OR (95%CI): 2.5 (1.1,5.5)<br><br>**Gc2-1f (n = 15)**<br>VDD: 53.3% (8)<br>Insuff.: 6.7% (1)<br>Suff.: 40.0% (6)<br>OR (95%CI): 3.3 (1, 10.6)<br><br>**Gc2-1s (n= 38)**<br>VDD: 65.8% (25)<br>Insuff.: 7.9% (3)<br>Suff.: 26.3% (10)<br>OR (95%CI): 5.5 (2.3–13.4)<br><br>**Gc2-2 (n= 8)**<br>VDD: 87.5% (7)<br>Insuff.: 12.5% (1)<br>Suff.: 0% (0)<br>OR (95%CI):20.1 (2.3, 177)<br><br>**Gc2 (n= 69)**<br>VDD: 68.1% (47)	P = 0.024<br><br>P = 0.047<br><br>P =0.0002<br><br>P = 0.007<br><br>P< 0.0001	5/8	VDD:<br>25(OH)D <20 ng/mL

Footnotes
All Alleles are noted as on the forward DNA strand. Notations in papers using the reverse DNA allele were adapted for easier comparison in this table.
† Statistical analyses in these studies were performed on log-transformed 25(OH)D values.
Haplotype codes explained (rs4588 x rs7041): Gc1s/1s: GG x CC; Gc1s/1f: GG x CA; Gc1f/1f: GG x AA; Gc: GT x CC; Gc2/1s: GT x CA; Gc2/1f: GT x AA. Gcx-x: TT x CC; Gc2-x: TT x CA; Gc2/2: TT x AA; Gc1f: GxA; Gc1s: GxC;; Gc2: TxA
Abbreviations: VDD, Vitamin D deficiency; Ref., reference haplotype

	Gene and SNP	Source	Vitamin											Ancestry	Outcome				Risk of bias	Notes
			Vitamin D					Vit. B12	Vit. B9 (Folate)			Vit. B6 (PLP)	Hcy							
			Calcifediol (25(OH)D)	Calcitriol (1.25(OH)2D)	Ergocalciferol (D2)	VDBP	Vit.D deficiency		Red blood cell folate	Folate / Folic acid	Serum / Plasma Folate				Absolute Values	Effect size	Other	P-value	JBI assessment	
		Zhang et al., 2013	x											Asian (Chinese)	*Mean [ng/mL]*<br><br>GG (Wt): 19.48<br>GA: 18.17<br>AA: 17.87	GG (Wt): 1<br>GA: 0.933<br>AA: 0.917	β= -0.1484<br><br>*Odds for VDD, OR (95%CI)*<br>1.215 (1.037, 1.424)	P = 0.004411 *<br>P = 0.016	7/8	* Bonferroni corrected
		Ammar et al., 2023	x											Arab			*Odds of non-response *, OR (95%CI)*<br><br>GG (Wt): 1<br>GA: 5.31 (1.21, 35.7)<br>AA: 6.92 (1.70, 28.31)	P = 0.011<br>P = 0.008	7/9	* non-response: serum 25(OH)D <30ng/mL after Vit. D supplementation
	**CYP2R1**<br>rs10766197	Xu et al., 2015					x							Asian (Chinese)<br>Uygur ethnic group			*Odds for VDD, OR (95%CI)*<br>6.533 (1.361, 31.357)<br><br>**Allele Freq.**<br>VDD<br>GG: 0.39<br>GA: 0.48<br>AA: 0.13<br><br>Control<br>GG: 0.69<br>GA: 0.12<br>AA: 0.19	P = 0.019	8/8	VDD:<br>25(OH)D < 20ng/mL
		Nissen, Vogel et al., 2014 [1, †]	x											Caucasian	*Geometric Mean (95% CI) [nmol/L]*<br><br>GG (Wt): 74.3 (71.6, 77.1)<br>GA: 72.9 (70.8, 75.1)<br>AA: 66.9 (64.2, 69.8)	GG (Wt): 1<br>GA: 0.981<br>AA: 0.900		P < 0.0001	6/8	
Vitamin D		Nissen et al., 2014 [1, †]	x											Caucasian	*Geometric Mean (95% CI) [nmol/L]*<br><br>GG (Wt): 73.0 (69.0, 77.3)<br>GA: 73.2 (69.9, 76.6)<br>AA: 66.2 (62.1, 70.5)	GG (Wt): 1<br>GA: 1.003<br>AA: 0.907		P = 0.0081	7/8	
	**CYP2R1**<br>rs10766197	Barry et al., 2014 [†]	x											Caucasian			**Baseline**<br>Estimated diff. In serum level per variant allele: -3.83%<br>95%CI: -5.79, -1.83<br><br>**After 1 yr D3 supplementation** [2]<br>Estimated diff. In serum level per variant allele: -4.12%<br>95%CI: -7.40, -0.76<br><br>Only optimally adherent subjects: -4.21%<br>95%CJ: -7.66, -0.63	P = 0.0002 (Wald test)<br><br>Genotype * D3:<br>P = 0.02 (Wald test)<br><br>Optimally adherent:<br>P = 0.02	7/8	
	**CYP2R1**<br>rs10766197	Bu et al., 2010	x											Caucasian			Discovery cohort<br>β = -6.45<br>Replication cohort<br>β = -3.55<br>Pooled<br>β = -4.53	Discovery<br>P (Wald test) = 0.005<br>P* = 0.005<br>P** = NS<br>Replication<br>P (Wald test) = 0.022<br>P* = 0.024<br>P** = NS<br>Pooled<br>P (Wald test) = 4.04E-4<br>P* = 3E-4<br>P** = 0.002	7/8	* empirical P-value<br>** adjusted for multiple testing
		Slow et al., 2020	x											Caucasian			*Baseline 25(OH)D [nmol/L]*<br>β (95% CI): -4.00 (-7.45, -0.54)	P = 0.03413	12/13	Each copy of the minor allele A for rs10766197 was associated with a 4.00 nmol/L lower 25(OH) D conc.
		Waterhouse et al., 2014 [3]	x											Caucasian			*Coef. (95%CI)*<br><br>Model 2: -3.7 (-6.2, -1.1)<br>Model 3: -3.5 (-6.0, -1.0)	P < 0.005 *<br>P = 0.01	12/13	* Remained significant after after Bonferroni correction<br>(P = 4.8E-3)

Footnotes
Effect directions refer to minor allele A; reference group: GG (Wt)
β = variation in serum 25(OH)D per risk-allele.
[†] Statistical analyses in these studies were performed on log-transformed 25(OH)D values.
[1] Both studies by Nissen et al. were conducted in the same cohort.
[2] Shows that the SNP negatively impacts the efficacy of Vit. D3 treatment to increase year 1 [25(OH)D]
[3] Models apply to both supplementation groups combined (30'000 + 60'000 IU/ month). Model 2 includes supplement dose, baseline serum 25(OH)D level, and SNPs. Model 3 includes variables from Model 2 + personal & environmental factors.

	Gene and SNP	Source	Calcifediol (25(OH)D)	Calcitriol (1.25(OH)2D)	Ergocalciferol (D2)	VDBP	Vit.D deficiency	Vit. B12	Red blood cell folate	Folate / Folic acid	Serum / Plasma Folate	Vit. B6 (PLP)	Hcy	Ancestry	Absolute Values	Effect size	Other	P-value	JBI assessment	Notes
		Batai et al., 2014 [†]	x											African-American & Caucasian			African American: β= 0.03<br>European American: β= 0.04	P = 0.02<br>P = 0.005	7/8	
		Barry et al., 2014 [†]	x											Caucasian			Baseline associations significantly associated, but results not shown in paper		7/8	
Vitamin D	**CYP2R1**<br>rs2060793	Engelman et al., 2012	x											Caucasian	*Association stratified by season* [1]<br>*[nmol/L]*<br>High exposure<br>GG (Wt): 58.4<br>GA: 65.6<br>AA: 67.3	GG (Wt): 1<br>GA: 1.123<br>AA: 1.152	Additive genetic model<br>β = 0.25<br><br>SNP * high exposure<br>β = 0.31	P < 0.001<br><br>P = 0.0001	7/8	
		Ahn et al., 2010 [2]	X											Caucasian	*Mean [nmol/L]*<br><br>NHS-CGEMS<br>GG (Wt): 77.0<br>GA: 83.3<br>AA: 80.5<br><br>NHS-T2D<br>GG (Wt): 53.8<br>GA: 58.0<br>AA: 59.3	NHS-CGEMS<br>GG (Wt): 1<br>GA: 1.082<br>AA: 1.045<br><br>NHS-T2D<br>GG (Wt): 1<br>GA: 1.078<br>AA: 1.102	NHS-CGEMS<br>β (SE): 0.28 (0.08)<br>NHS-T2D<br>β(SE): 0.22 (0.07)	NHS-CGEMS<br>P = 3.5E-4<br>NHS-T2D<br>P = 8.9E-4	6/8	rs1993116 was r2=1 with rs2060793 in the HapMap CEU panel

Footnotes
Effect directions refer to minor allele A; reference group: GG (Wt).
β = per risk-allele variation in log-25(OH)D conc.
[†] Statistical analyses in these studies were performed on log-transformed 25(OH)D values.
[1] association not significant for low sun exposure
[2] Ahn et al (2020) analyzed 5 GWAS for associations between SNPs and 25(OH)D, the cohorts listed here found significant results. Subjects include cases and contros.

	Gene and SNP	Source	Calcifediol (25(OH)D)	Calcitriol (1.25(OH)2D)	Ergocalciferol (D2)	VDBP	Vit.D deficiency	Vit. B12	Red blood cell folate	Folate / Folic acid	Serum / Plasma Folate	Vit. B6 (PLP)	Hcy	Ancestry	Absolute Values	Effect size	Other	P-value	JBI assessment	Notes
Vitamin D	rs12794714 x rs10741657 x rs2060793	Barry et al., 2014 [†]	x											Caucasian			Estimated % diff. In serum level per variant allele (95%CI)<br>AGC: Ref.<br>GAT: 5.82 (3.49, 8.20)<br>GGC: 2.95 (-0.05, 6.03)	P < 0.0001<br>P = 0.05	7/8	

Footnotes
[†] Statistical analyses in this studywere performed on log-transformed 25(OH)D values.

	Gene and SNP	Source	Vitamin											Ancestry	Outcome				Risk of bias	Notes
			Vitamin D					Vit. B12	Vit. B9 (Folate)			Vit. B6 (PLP)	Hcy							
			Calcifediol (25(OH)D)	Calcitriol (1.25(OH)2D)	Ergocalciferol (D2)	VDBP	Vit.D deficiency		Red blood cell folate	Folate / Folic acid	Serum / Plasma Folate				Absolute Values	Effect size	Other	P-value	JBI assessment	
Vitamin D	**CYP2R1**<br>rs1562902	Nissen et al., 2014 †	x											Caucasian	*Geometric Mean (95% CI) [nmol/L]*<br><br>TT (Wt): 67.5 (63.9, 71.4)<br>TC: 73.3 (70.0, 76.6)<br>CC: 73.4 (68.6, 78.5)	TT (Wt): 1<br>TC: 1.086<br>CC: 1.087		P = 0.0353	7/8	
		Barry et al., 2014 †	x											Caucasian			Estimated diff. In serum level per variant allele: 3.30%<br>95%CI: 1.21, 5.42	P = 0.002	7/8	
		Bu et al., 2010	x											Caucasian			Discovery cohort<br>β= 6.08<br>Discovery + Replication cohort<br>β= 2.69	Discovery<br>P (Wald test) = 0.011<br>P*= 0.011<br>P**= NS<br>Pooled<br>P (Wald test) = 0.030<br>P*= 0.031<br>P**= NS	7/8	* empirical P-value<br>** adjusted for multiple testing

Footnotes
Effect directions refer to minor allele C (G on reverse DNA strand); reference group: TT (Wt) (AA on reverse DNA strand)
β = variation in serum 25(OH)D per risk-allele.
† Statistical analyses in these studies were performed on log-transformed 25(OH)D values.
rs1562902 was also associated with 25(OH)D when occurring with rs10766197 (view Haplotype rs1562902 x rs10766197)

Vitamin D	rs1562902 x rs10766197	Barry et al., 2014 †	x											Caucasian			Estimated % diff. In serum level per genotype (95%CI)<br>AA: Ref.<br>AG: 3.89 (1.67, 6.15)<br>GG: 4.29 (0.56, 8.15)	AG: P= 0.001<br>GG: P= 0.02	7/8	

Footnotes
† Statistical analyses in this studywere performed on log-transformed 25(OH)D values.

Vitamin D	**CYP2R1**<br>rs11023374	Fohner et al., 2016	x											Alaskan Native (Yup'ik)	Mixed-effects model linear regression 1 (n= 743)<br>*Mean ± SE [ng/mL]*<br><br>TT (Wt) + CT: 31.3 ± 0.5<br>CC: 25.0 ± 2.2	TT + CT : 1<br>CC: 0.799	Mixed-effects model linear regression 2 (n=677)<br>TT (Wt) + CT: Ref.<br>CC: β (SE) = -3.8 (1.7)<br>Heritability: 0.46<br><br>MLR (n=526)<br>TT (Wt) + CT: Ref.<br>CC: β (SE) = -4.5 (2.0)	Mixed-model 1:<br>P = 0.0067<br><br>Mixed model 2:<br>P = 2.3E-2<br><br>MLR: P= 1.5E-2	6/8	Specified outcome measure: 25(OH)D3
		Engelman et al., 2012	x											Caucasian	*Association stratified by season [1] [nmol/L]*<br>High exposure<br>AA (Wt): 66.4<br>AG: 60.2<br>GG: 59.4	AA (Wt): 1<br>AG: 0.907<br>GG: 0.895	Additive genetic model<br>β = -0.19<br><br>SNP * high exposure<br>β = -0.29	P=0.005<br><br>P = 0.001	7/8	

Footnotes
Effect directions refer to minor allele C (G on reverse DNA strand); reference group: TT (wt) (AA on reverse DNA strand)
β = variation in serum 25(OH)D per risk-allele.
† Statistical analyses in these studies were performed on log-transformed 25(OH)D values.
[1] association not significant for low sun exposure (winter)
Abbreviations: Wt, wild type; MLR, Multiple linear regression

Vitamin D	**CYP2R1**<br>rs1993116	Robien et al., 2013 †	x											Asian (Chinese)	*Geometric Mean (95% CI), [nmol/L]*<br><br>CC (Wt): 64.6 (62.6, 66.7)<br>CT: 67.8 (65.5, 70.2)<br>TT: 68.5 (63.5, 73.9)	CC (Wt): 1<br>CT: 1.050<br>TT: 1.060	Variance explained: 0.8%	P for trend = 0.04	7/8	
		Batai et al., 2014 †	x											African-American & Caucasian			African American: β = 0.03<br>European American: β = 0.04	P = 0.02<br>P = 0.0006	7/8	VDD:<br>25(OH)D <20 ng/mL (<50nmol/L)
							x										*Odds for VDD in European Americans*<br>OR (95%CI): 0.51 (0.35, 0.76)	P = 0.0008		
		Li et al., 2014	x											Asian (Chinese)			β (SE): -1.789 (0.865)	P = 0.04<br>P = 0.30 *	8/8	* corrected for Bonferroni
		Ahn et al., 2010 [1]	x											Caucasian	*Mean [nmol/L]*<br><br>NHS-Polyps<br>GG (Wt): 62.5<br>GA: 68.8<br>AA: 75.0<br><br>NHS-CRC<br>GG (Wt): 65.5<br>GA: 68.8<br>AA: 73.8<br><br>HPFS<br>GG (Wt): 56.0<br>GA: 62.3<br>GG: 67.0	NHS-Polyps<br>GG (Wt):1<br>GA: 1.101<br>AA: 1.200<br><br>NHS-CRC<br>GG (Wt): 1<br>GA: 1.050<br>AA: 1.127<br><br>HPFS<br>GG (Wt): 1<br>GA: 1.113<br>GG: 1.196	NHS-Polyps<br>β(SE): -0.39 (0.07)<br><br>NHS-CRC<br>β(SE): 0.29 (0.08)<br><br>HPFS<br>β(SE): 0.33 (0.06)	NHS-Polyps<br>P= 6.2E-9<br><br>NHS-CRC<br>P= 5.2E-4<br><br>HPFS<br>P= 1.6E-8	6/8	rs1993116 was r2=1 with rs2060793 in the HapMap CEU panel
		Slow et al., 2020	x											Caucasian			*Increase per minor allele*<br><br>*Baseline 25(OH)D [nmol/L]*<br>β (95% CI): 3.95 (0.49, 7.41)<br><br>*Baseline free 25(OH)D [pmol/L]*<br>β (95% CI): 2.00 (0.16, 3.84)	P= 0.02584<br><br>P = 0.03409	P= 0.02584<br><br>P = 0.03409	Associations did not remain significant after Bonferroni correction

Footnotes
Effect directions refer to minor allele T ; reference group: CC (wt)
β = variation in serum 25(OH)D per risk-allele
† Statistical analyses in these studies were performed on log-transformed 25(OH)D values.
[1] Ahn et al (2020) analyzed 3 case-control studies for associations between SNPs and 25(OH)D, the cohorts listed here found significant results. Subjects include cases and contros.
A trend toward association with log-25(OH)D levels was observed in a Chinese cohorts too, however associations were not significant (P = 0.08) (Lu et al., 2011)
Abbreviations: NHS-Polyps, NHS colon polyp study; NHS-CRC, NHS colorectal cancer study; HPFS, study of prostatecancer in the Health Professionals Follow-up Study

	Gene and SNP	Source	Vitamin											Ancestry	Outcome				Risk of bias	Notes
			Vitamin D					Vit. B12	Vit. B9 (Folate)			Vit. B6 (PLP)	Hcy							
			Calcifediol (25(OH)D)	Calcitriol (1.25(OH)2D)	Ergocalciferol (D2)	VDBP	Vit.D deficiency		Red blood cell folate	Folate / Folic acid	Serum / Plasma Folate				Absolute Values	Effect size	Other	P-value	JBI assessment	
Vitamin D	**CYP2R1**<br>rs7116978	Tomei et al., 2020	**x**											Arab			*Genotype frequency by Vit.D status*<br><br>Deficient: **0% CC**, 80% CT, 20% TT<br>Insuff.: **13% CC**, 80% CT, 7% TT<br>Sufficient: **30% CC**, 45% CT, 25% TT<br><br>"CC genotype showed higher response to Vit. D supplementation than CT or TT (no data)"	P = 0.0163	3/9	VDD:<br>25(OH)D < 20ng/mL<br>(50nmol/L)<br><br>Insufficiency:<br>21 < 29 ng/mL<br><br>Sufficiency:<br>≥ 30ng/mL
		Nissen et al., 2014 †	**x**											Caucasian	*Geometric Mean (95% CI)*<br>*[nmol/L]*<br><br>CC (Wt): 67.5 (64.2, 71.0)<br>CT: 72.8 (69.5, 76.3)<br>TT: 77.5 (71.8, 83.8)	TT (Wt): 1<br>TC: 1.079<br>CC: 1.148		P = 0.0093	7/8	

Footnotes
Effect directions refer to minor allele T; reference group: CC (wt)
† Statistical analyses in this study were performed on log-transformed 25(OH)D values.

Vitamin D	**CYP2R1**<br>rs10500804	Lee et al., 2021	**x**											Asian (Korean)	*Mean ± SE [ng/mL]*<br><br>Total<br>TT (Wt): 20.0 ± 0.22<br>GT: 18.9 ± 0.20<br>GG: 18.0 ± 0.33<br><br>Summer/Fall<br>TT (Wt): 25.3 ± 0.31<br>GT: 23.7 ± 0.24<br>GG: 22.0 ± 0.38	Total<br>TT (Wt): 1<br>TG: 0.945<br>GG: 0.900<br><br>Summer/Fall<br>TT (Wt): 1<br>TG: 0.937<br>GG: 0.870	*Associations in multivariate model*<br><br>β = - 1.0149<br>P = 3.02E-8<br><br>SNP * Season<br>β = - 1.4964<br>P = 8.01E-5	Total<br>P = 1.79E-7<br><br>Summer/Fall<br>P = 6.56E-9	7/8	
		Elkum et al., 2014	**x**											Arab	*Mean [ng/mL]*<br><br>TT (Wt): 14.4,<br>TG: 14.3<br>GG: 12.0	TT (Wt): 1<br>TG: 0.993<br>GG: 0.933		P= 0.0379	6/8	
		Engelman et al., 2012	**x**											Caucasian	*Association stratified by season*[1]<br>*[nmol/L]*<br>High exposure<br>AA (Wt): 66.0<br>AC: 63.9<br>CC: 57.1	AA (Wt): 1<br>AC: 0.968<br>CC: 0.865	Additive genetic model<br>β = - 0.19<br><br>SNP * high exposure<br>β = - 0.25	P = 0.001<br><br>P = 0.002	7/8	

Footnotes
Effect directions refer to minor allele G (C on reverse stand) ; reference group: TT (Wt) (AA on reverse strand)
1 association not significant for low sun exposure (winter)

Vitamin D	**CYP2R1**<br>rs731236	Tomei et al., 2020	**x**											Arab			*Genotype frequency by Vit.D status*<br><br>Deficient: 0**%** GG, 20% AG, 80% AA<br>Insuff.: 0**%** GG, 5% AG, 95% AA<br>Sufficient: **4%** GG, 44% AG, 54% AA<br><br>"GG genotype showed higher response to Vit. D supplementation than GA or AA (no data)"	P = 0.0336	3/9	VDD:<br>25(OH)D < 20ng/mL<br>(50nmol/L)<br><br>Insufficiency:<br>21 < 29 ng/mL<br><br>Sufficiency:<br>≥ 30ng/mL
		Nissen et al., 2014 †	**x**											Caucasian	*Geometric Mean (95% CI)*<br>*[nmol/L]*<br><br>TT (Wt): 69.3 (67.0, 71.7)<br>TC: 73.2 (71.1, 75.4)<br>CC: 73.7 (70.1, 77.5)	TT (Wt): 1<br>TC: 1.056<br>CC: 1.063		P = 0.0346	7/8	

Footnotes
Effect directions refer to minor allele G (C on reverse strand) ; reference group: AA (Wt) (TT on reverse strand)
† Statistical analyses in this study were performed on log-transformed 25(OH)D values.
Data by Nissen et al (2014) refer to the entire cohort, which includes adults and children (not adults only).

	Gene and SNP	Source	Calcifediol (25(OH)D)	Calcitriol (1.25(OH)2D)	Ergocalciferol (D2)	VDBP	Vit.D deficiency	Vit. B12	Vit. B9 (Folate): Red blood cell folate	Vit. B9 (Folate): Folate / Folic acid	Vit. B9 (Folate): Serum / Plasma Folate	Vit. B6 (PLP)	Hcy	Ancestry	Outcome: Absolute Values	Outcome: Effect size	Outcome: Other	Outcome: P-value	Risk of bias: JBI assessment	Notes
Vitamin D		Sallinen et al., 2021, †	x											Caucasian	*Mean ± SD [nmol/L]*<br><br>GG (Wt): 65.9 ± 12.5<br>GA: 68.4 ± 13.1<br>AA: 69.9 ± 13.9	GG (Wt): 1<br>GA: 1.038<br>AA: 1.061	β = 2.2	P = 4.9E-12	6/8	
		Robien et al., 2013 †	x											Asian (Chinese)	*Geometric Mean (95%CI) [nmol/L]*<br><br>GG (Wt): 64.9 (62.9, 67.0)<br>GA: 67.6 (65.3, 70.0)<br>AA: 70.0 (65.3, 75.3)	GG (Wt): 1<br>GA: 1.042<br>AA: 1.079	Variance explained: 1%	P for trend = 0.02	7/8	
		Nissen, Vogel et al., 2014 [1, †]	x											Caucasian	*Geometric Mean (95%CI) [nmol/L]*<br><br>GG (Wt): 67.2 (65.0, 69.5)<br>GA: 73.9 (71.8, 76.1)<br>AA: 76.6 (73.0, 80.5)	GG (Wt): 1<br>GA: 1.1<br>AA: 1.140		P < 0.0001	6/8	
	**CYP2R1**<br>rs10741657	Nissen et al., 2014 [1, †]	x											Caucasian	*Geometric Mean (95%CI) [nmol/L]*<br><br>GG (Wt): 66.6 (63.3, 70.1)<br>GA: 74.0 (70.7, 77.4)<br>AA: 75.2 (69.9, 80.9)	GG (Wt): 1<br>GA: 1.111<br>AA: 1.129		P= 0.0067	7/8	
		Li et al., 2014	x											Asian (Chinese)			β(SE): -1.866 (0.863)	P = 0.04<br>P*= 0.30	8/8	* corrected for Bonferroni
		Grant et al., 2022	x											Mixed		*Adjusted Mean Difference (95%CI), [nmol/L]*<br><br>GG: Ref. (0.00)<br>GA: 2.94 (1.38, 4.50)<br>AA: 4.44 (2.38, 6.51)		both P < 0.001	7/8	
		Lafi et al., 2015	x											Mostly Caucasian (Mixed)	*Mean ± SD [ng/mL]*<br><br>GG (Wt): 25.3 ± 18.1<br>GA: 31.8 ± 21.4<br>AA: 38.7 ± 24.9<br><br>GG + AG: 28.5 ± 20.0	GG (Wt): 1<br>GA: 1.257<br>AA: 1.530		GA: P= 0.013<br>AA: P= 0.026<br><br>AA+AG: P= 0.004	5/8	Associations for GG + AG NS
							x										*Odds for low Vit. D, OR (95%CI)*<br><br>GG (Wt):Ref.<br>GA: 0.47 (0.26, 0.85)<br>AA: 0.33 (0.12, 0.87)<br><br>AA+AG: 0.44 (0.25–0.77)			VDD:<br>25(OH) < 20 ng/mL
		Barry et al., 2014 †	x											Caucasian			Estimated diff. In serum level per variant allele: 4.91%<br>95%CI: 2.76, 7.10	P < 0.0001 (Wald test)	7/8	
	**CYP2R1**<br>rs10741657	Bu et al., 2010	x											Caucasian			Discovery cohort<br>β= 7.56<br>Replication cohort<br>NS<br>Pooled<br>β= 4.12	Discovery cohort<br>P (Wald test) = 0.001<br>P*= 0.002<br>P**= 0.051<br>Replication cohort<br>NS<br>Pooled<br>P (Wald test) = 0.002<br>P*= 0.002<br>P**= 0.010	7/8	* empirical P-value<br>** adjusted for multiple testing
		Wang et al., 2010 †	x											Caucasian	*Mean ± SE [nmol/L]*<br><br>Framingham heart study<br>Wt: 75.4 ± 0.87<br>Het.: 78.6 ± 0.76<br>Hom.: 81.6 ± 1.26<br><br>1958 British Birth Cohort<br>Wt: 56.8 ± 0.34<br>Het.: 60.2 ± 0.32<br>Hom.: 61.1 ± 0.29	Framingham Heart<br>Wt: 1<br>Het.: 1.042<br>Hom.: 1.082<br><br>1958 British Birth<br>Wt: 1<br>Het.: 1.060<br>Hom.: 1.076		*Candidate gene analysis*<br>Discovery Cohort<br>P= 3.91E-8<br>Replication Cohort<br>P= 2.09E-14<br>Overall<br>P= 3.27E-20	8/8	All associations reached genome-wide significance (P < 5E-8)
	**CYP2R1**<br>rs10741657						x										*Odds for Vit. D insufficiency per risk allele*<br>*OR (95%CI)*<br><br><75nmol/L: 1.21 (1.45-1.29)<br><50nmol/L: 1.06 (1.00-1.13)	P = 9.4E-11<br>P = 0.06		
		Batai et al., 2014 †	x											African-American & Caucasian			African American: β= 0.04<br>European American: β= 0.04	P= 0.01<br>P= 0.003	7/8	
		Elkum et al., 2014	x											Arabs	*Mean [ng/mL]*<br><br>GG (Wt): 13.4<br>GA: 14.6<br>AA: 16.2	GG (Wt): 1<br>GA: 1.090<br>AA: 1.209		P= 0.0437	6/8	
		Didriksen et al., 2013	x											Caucasian	*Mean ± SD [nmol/L]*<br><br>Wt.: 55.6 ± 21.9<br>Het.: 56.2 ± 22.5<br>Hom.: 61.4 ± 24.4	Wt.: 1<br>Het.: 1.011<br>Hom.: 1.104	*Increase (delta) in serum 25(OH)D after Vit.D supplementation*<br><br>Hom. baseline: 64.0 ± 26.6<br>Hom. 6mo: 176.8 ± 37.4<br>MD: 112.8± 33.0	Hom.: P < 0.05<br><br>Delta: P < 0.01	7/8	Difference for heterozygotes NS
		Slater et al., 2017					x							Caucasian			*Odds to be VDD, OR (95%CI)*<br><br>Initial model<br>3.67 (1.35, 9.99)<br><br>Final model:<br>4.040 (1.429, 11.420)	P= 0.011<br><br>P= 0.008	7/8	Initial model: all potential confounders final model: significant confounders only<br><br>VDD:<br>25(OH)D < 30ng/mL
		Simon et al., 2011	x											Caucasian			"Each additional 'A' allele was associated with a 5.3 nmol/L increase in 25(OH)D level"		5/8	

Footnotes
Effect directions refer to minor allele A (T on reverse DNA strand); reference genotype: GG (wt) (CC on reverse DNA strand)
β = variation in serum 25(OH)D per risk-allele, Sallinen et al (2021) refer to changes in log-25(OH)D
† Statistical analyses in these studies were performed on log-transformed 25(OH)D values.
[1] Both studies by Nissen et al. were conducted in the same cohort.
Abbreviations: NS, not significant; hom., homozygote; het., heterozygote; VDD, Vitamin D deficiency

	Gene and SNP	Source	Vitamin											Ancestry	Outcome				Risk of bias	Notes
			Vitamin D					Vit. B12	Vit. B9 (Folate)			Vit. B6 (PLP)	Hcy							
			Calcifediol (25(OH)D)	Calcitriol (1.25(OH)2D)	Ergocalciferol (D2)	VDBP	Vit.D deficiency		Red blood cell folate	Folate / Folic acid	Serum / Plasma Folate				Absolute Values	Effect size	Other	P-value	JBI assessment	
Vitamin D	**CYP2R1**<br>rs12794714	Robien et al., 2013 [†]	x											Asian (Chinese)	*Geometric Mean (95%CI) [nmol/L]*<br><br>GG (Wt): 69.2 (66.8, 71.7)<br>GA: 66.0 (64.0, 68.2)<br>AA: 58.6 (54.9, 62.5)	GG (Wt): 1<br>GA: 0.954<br>AA: 0.847	Variance explained: 3.1%	P for trend < 0.001	7/8	
Vitamin D		Barry et al., 2014 [†]	x											Caucasian			**Baseline**<br>Estimated diff. In serum level per variant allele: -4.74<br>95%CI: -6.68, -2.75<br><br>**After 1 yr D3 supplementation** [1]<br>*Analysis restricted to optimally adherent subjects*<br><br>Estimated diff. In serum level per variant allele: -3.69%<br>95%CI: -7.18, -0.07	P < 0.0001 (Wald test)<br><br>P = 0.05	7/8	
Vitamin D		Bu et al., 2010	x											Caucasian	*Mean ± SE [nmol/L] (ANOVA)*<br><br>Discovery cohort<br>GG: 77.82 ± 21.96<br>GA: 70.65 ± 21.70<br>AA: 62.03 ± 17.82<br><br>Replication cohort<br>GG: 75.09 ± 23.67<br>GA: 74.94 ± 19.90<br>AA: 66.13 ± 16.18<br><br>Pooled<br>GG: 76.62 ± 23.32<br>GA: 74.44 ± 20.83<br>AA: 65.20 ± 16.96	Discovery cohort<br>GG: 1<br>GA: 0.908<br>AA: 0.797<br><br>Replication cohort<br>GG: 1<br>GA: 0.998<br>AA: 0.881<br><br>Pooled<br>GG: 1<br>GA: 0.972<br>AA: 0.851	Discovery cohort<br>β = -7.63<br>Replication cohort<br>β = -3.61<br>Pooled<br>β = -5.03	Discovery cohort<br>P ANOVA = 0.001<br>P (Wald test) = 0.001<br>P*= 0.001<br>P**= 0.022<br>Replication cohort<br>P ANOVA = 0.017<br>P (Wald test) = 0.018<br>P*= 0.016<br>P**= NS<br>Pooled<br>P ANOVA = 0.0002<br>P (Wald test) = 5.74E-5<br>P*= 1E-4<br>P**= 1E-4	7/8	* empirical P-value<br>** adjusted for multiple testing
Vitamin D		Batai et al., 2014 [†]	x											African-American & Caucasian			African American: β= -0.04<br>European American: β= -0.04	P = 0.01<br>P = 0.005	7/8	VDD:<br>25(OH)D <20 ng/mL<br>(<50nmol/L)
Vitamin D							x										*Odds for VDD in AA*<br>OR (95%CI): 1.72 (1.20, 2.47)	P = 0.003		
Vitamin D	**CYP2R1**<br>rs12794714	Elkum et al., 2014	x											Arab	*Mean [ng/mL]*<br><br>GG (Wt): 14.4<br>GA: 14.3<br>AA: 12.0	GG (Wt): 1<br>GA: 0.993<br>AA: 0.833		P= 0.0402	6/8	
Vitamin D		Ammar et al., 2023	x											Arab			*Odds of non-response *, OR (95%CI)*<br><br>GG (Wt): 1<br>GA: 3.45 (1.49, 7.99)<br>AA: 5.09 (1.70, 24.08)	P = 0.014<br>P = 0.004	7/9	* non-response: serum 25(OH)D <30ng/mL after Vit. D supplementation
Vitamin D		Slow et al., 2020	x											Caucasian			*Baseline 25(OH)D [nmol/L]*<br>β (95% CI): -4.62 (-8.15, -1.08)<br><br>*Baseline free 25(OH)D [pmol/L]*<br>β (95% CI): -2.71 (-4.61, -0.81)	P= 0.01095<br><br>P= 0.00545	12/13	
Vitamin D		Kwak et al., 2018	x											Asian (Korean)			"found negative associations btw serum 25(OH)D level and the effect allele A"	P = 0.020	7/8	
Vitamin D		Hansen et al., 2015	x											African- American			*Estimated change in serum 25(OH)D per A allele*<br>β ± SE = 1.30 ± 0.56	P= 0.02	7/8	This SNP was imputed in this cohort

Footnotes
Effect directions refer to minor allele A; reference group: GG (wt)
β = variation in serum 25(OH)D per risk-allele
[†] Statistical analyses in these studies were performed on log-transformed 25(OH)D values.
[1] shows that the SNP negatively impacts the efficacy of Vit. D3 treatment to increase year 1 [25(OH)D]

Vitamin D	**CYP2R1**<br>rs7936142 x rs12794714 x rs2060793 x rs16930609	Hansen et al., 2015	x											African- American			**AAGA**<br>β = 0.0899<br>frequency = 0.1332	P = 0.1123	7/8	
Vitamin D	**CYP24A1**<br>rs6013897	Wang et al., 2010 [†]	x											Caucasian				*Candidate gene analysis*<br>Discovery Cohort<br>P = 7.2 E-4 (NS)<br>Replication Cohort<br>P = 8.4E-8<br>Overall<br>P = 6.0E-10 *	8/8	* Genome-wide significant
Vitamin D		Ammar et al., 2023	x											Arab	Mean [median min, max] *[ng/mL]*<br><br>TT (Wt): 20.6 (6.3, 24.9)<br>TG: 12.4 (0, 24.4)<br>GG: 13.5 (3.9, 23.3)	TT: 1<br>TG: 0.602<br>GG: 0.655		P < 0.001	7/9	
Vitamin D		Barry et al., 2014 [†]	x											Caucasian			**After 1 yr D3 supplementation** [1]<br>Estimated diff. In serum level per variant allele: -4.24 %<br>95%CI: -8.22, -0.09<br><br>Only optimally adherent subjects: -4.86 %<br>95%CI: -9.10, -0.42	Genotype * D3<br>P = 0.04 (Wald test)<br><br>Optimally adherent<br>P =0.03	7/8	

Footnotes
Effect directions refer to minor allele A; reference group: TT (Wt)
β = variation in serum 25(OH)D per risk-allele
[†] Statistical analyses in these studies were performed on log-transformed 25(OH)D values.
[1] shows that the SNP negatively impacts the efficacy of Vit. D3 treatment to increase year 1 [25(OH)D]

Vitamin D	**CYP24A1**<br>rs2762939	Szili et al., 2018	x											Caucasian	*Mean total-25(OH)D [ng/mL]*<br><br>GG(Wt): 15.5<br>GC: 14.0<br>CC: 11.3	GG (Wt): 1<br>GC: 0.903<br>CC: 0.729		GG (Wt) vs. CC<br>P= 0.018 *	7/8	* remained signif. after FDR correction, became non-significant after adjusting for BMI, age, and sex.
Vitamin D		Barry et al., 2014 [†]	x											Caucasian			Estimated diff. In serum level per variant allele: 2.75%<br>95%CI: 0.32, 5.23	P= 0.03 (Wald test)	7/8	

Footnotes
Effect directions refer to minor allele C ; reference group: GG (Wt)
[†] Statistical analyses in these studies were performed on log-transformed 25(OH)D values.
rs2762939 was also associated with 25(OH)D when occurring with rs4809958 and rs2244719 (view Haplotype rs2762939 x rs4809958 x rs2244719)

Vitamin D	rs2762939 x rs4809958 [1] x rs2244719	Barry et al., 2014 [†]	x											Caucasian			Ref.: GTC<br>Estimated % diff. in baseline 25(OH)D level for GGT: -3.11%<br>95%CI: 0.50, 5.79	P = 0.02	7/8	

Footnotes
[1] Associations of rs4809958 were not replicated in this review
[†] Statistical analyses in these studies were performed on log-transformed 25(OH)D values.
Abbreviations: Ref., reference haplotype

	Gene and SNP	Source	Vitamin											Ancestry	Outcome				Risk of bias	Notes
			Vitamin D					Vit. B12	Vit. B9 (Folate)			Vit. B6 (PLP)	Hcy							
			Calcifediol (25(OH)D)	Calcitriol (1.25(OH)2D)	Ergocalciferol (D2)	VDBP	Vit.D deficiency		Red blood cell folate	Folate / Folic acid	Serum / Plasma Folate				Absolute Values	Effect size	Other	P-value	JBI assessment	
Vitamin D	**CYP24A1**<br>rs2209314	Szili et al., 2018	x											Caucasian	*Mean total-25(OH)D [ng/mL]*<br>TT (Wt): 15.6<br>TC: 13.6<br>CC: 13.1<br><br>*Mean free-25(OH)D [pmol/L]*<br>TT (Wt): 9.9<br>TC: 8.9<br>CC: 5.8<br><br>*Mean Bioavailable-25(OH)D [nmol/mL]*<br>TT (Wt): 4.1<br>TC: 3.6<br>CC: 3.1	Total<br>TT (Wt): 1<br>TC: 0.872<br>CC: 0.840<br><br>Free<br>TT (Wt): 1<br>TC: 0.899<br>CC: 0.586<br><br>Bioavailable<br>TT (Wt): 1<br>TC: 0.878<br>CC: 0.756	Total<br>β (SE): −5.787 (2.844)<br>P = 0.005<br>Explained variance: 2.7%	Total: P = 0.017 *<br><br>Free: P = 0.012<br><br>Bioavailable: P = 0.008	7/8	* remained signif. after FDR correction but non-significant after adjusting for BMI, age, and sex.<br><br>P-values by Szili et al. (2018) refer to the differences between Wt and CC genotype
						x									*Mean, [mg/L]*<br><br>TT (Wt): 306.2<br>TC: 309.8<br>CC: 360.9	TT (Wt): 1<br>TC: 1.012<br>CC: 1.179		P= 0.036		
		Barry et al., 2014 [†]	x											Caucasian			Estimated diff. In serum level per variant allele: 2.67%<br>95%CI: 0.19, 5.21	P = 0.03 (Wald test)	7/8	

Footnotes
Effect directions refer to minor allele C ; reference group: TT (Wt)
[†] Statistical analyses in this study were performed on log-transformed 25(OH)D values.
Abbreviations: Wt, wild type; FDR, False discovery rate

	Gene and SNP	Source	Calcifediol (25(OH)D)	Calcitriol (1.25(OH)2D)	Ergocalciferol (D2)	VDBP	Vit.D deficiency	Vit. B12	Red blood cell folate	Folate / Folic acid	Serum / Plasma Folate	Vit. B6 (PLP)	Hcy	Ancestry	Absolute Values	Effect size	Other	P-value	JBI assessment	Notes
Vitamin D	**VDR**<br>rs10783219	Rivera-Paredez et al., 2018					x							Amerindian (Hispanic-Caucasian range)			**Subsample (n=400) ***<br>*Odds to be VDD per variant allele A , OR (95%CI)*<br>1.40 (1.03, 1.90)	P= 0.031	8/8	VDD:<br>25(OH)D <20 ng/mL<br><br>* The associations were no longer significant when tested in a larger sample (P = 0.377, n=689)
		Engelman et al., 2008	x											Hispanic			Coef (SE) : -0.164 ± 0.056	P= 0.004	6/8	Data from San Antonio Hispanic subjects
		Barry et al., 2014 [†]	x											Caucasian			**After 1 yr D3 supplementation** [1]<br>*Analysis restricted to optimally adherent subjects*<br><br>Estimated diff. In serum level per variant allele: -3.75 %<br>95%CI: -7.41, 0.06	P= 0.05	7/8	Optimally adherent:<br>took ≥80% of pills, no gaps in pill taking ≥7 days, no personal Vit. D supplementation

Footnotes
Effect directions refer to minor allele A (T on reverse strand) ; reference group: TT (Wt) (AA on reverse strand)
[†] Statistical analyses in this study were performed on log-transformed 25(OH)D values.
[1] shows that the SNP negatively impacts the efficacy of Vit. D3 treatment to increase year 1 [25(OH)D]
Abbreviations: Wt, wild type; VDD, Vitamin D deficiency; Coef., coefficient; Ref., reference

	Gene and SNP	Source	Calcifediol (25(OH)D)	Calcitriol (1.25(OH)2D)	Ergocalciferol (D2)	VDBP	Vit.D deficiency	Vit. B12	Red blood cell folate	Folate / Folic acid	Serum / Plasma Folate	Vit. B6 (PLP)	Hcy	Ancestry	Absolute Values	Effect size	Other	P-value	JBI assessment	Notes
Vitamin D	**VDR**<br>rs7139166	Barry et al., 2014 [†]	x											Caucasian			**After 1 yr D3 supplementation** [1]<br>*Analysis restricted to optimally adherent subjects*<br><br>Estimated diff. In serum level per variant allele: 3.99%<br>95%CI: 0.20, 7.92	P = 0.04	7/8	
		Rivera-Paredez et al., 2018					x							Amerindian (Hispanic-Caucasian range)			**Subsample (n=400) ***<br><br>*Odds to be VDD per variant allele , OR (95%CI)*<br>1.93 (1.35, 2.75)	P = 0.001<br>P = 0.011<br>P < 0.001	8/8	VDD:<br>25(OH)D <20 ng/mL<br><br>* this variant was not further examined in the whole cohort (n=689)

Footnotes
Effect directions refer to minor allele G; reference group: CC (Wt)
[†] Statistical analyses in this study were performed on log-transformed 25(OH)D values.
[1] shows that the SNP positively impacts the efficacy of Vit. D3 treatment to increase year 1 [25(OH)D]
Abbreviations: Wt, wild type; VDD, Vitamin D deficiency; Coef., Ref., reference haplotype

	Gene and SNP	Source	Calcifediol (25(OH)D)	Calcitriol (1.25(OH)2D)	Ergocalciferol (D2)	VDBP	Vit.D deficiency	Vit. B12	Red blood cell folate	Folate / Folic acid	Serum / Plasma Folate	Vit. B6 (PLP)	Hcy	Ancestry	Absolute Values	Effect size	Other	P-value	JBI assessment	Notes
Vitamin D	**VDR**<br>rs2228570	Liu et al., 2021			x									Asian (Chinese)			*OR (95%CI)*<br><br>AA (Wt): Ref.<br>GA: 4.400 (0.839, 23.073)<br>GG: 5.371 (1.011, 28.543)	P = 0.08 (NS)<br>P = 0.049	8/10	
		Waterhouse et al., 2014 [1]	x											Caucasian			*Coef. (95%CI)*<br><br>Model 2: 2.5 (-0.1, 5.2)<br>Model 3: 2.6 (0.0, 5.2)	P = 0.06<br>P = 0.05	12/13	
		Kandemiş et al., 2021	x											Caucasian (Turkish Cypriots)	*Mean ± SD [ng/mL]*<br><br>CC (Wt): 28.6 ± 8.7<br>CT + TT: 22.1 ± 8.5	CC (Wt): 1<br>CT + TT: 0.773		P = 0.029	6/8	
		Mohamed et al., 2022	x											Arab	*Mean ± SD [ng/mL]*<br><br>CC (Wt): 16.75 ± 2.05<br>CT+ TT: 9.25 ± 2.30	CC: 1<br>CT+TT: 0.552		P = 0.001	5/10	The SNP was also linked to anemia and Hb levels in this review, however these results were not replicated
		Tuncel et al., 2019	x											Caucasian (Turkish Cypriots)	*Mean ± SD [ng/mL]*<br><br>CC (Wt): 27.2 ± 8.8<br>CT+ TT: 22.1 ± 9.2	CC (Wt): 1<br>CT + TT: 0.813		P = 0.023	5/8	

Footnotes
Effect directions refer to minor allele T; reference group: CC (Wt)
[1] Models apply to both supplementation groups combined (30'000 + 60'000 IU/ month). Model 2 includes supplement dose, baseline serum 25(OH)D level, and SNPs. Model 3 includes variables from Model 2 + personal & environmental factors.

	Gene and SNP	Source	Calcifediol (25(OH)D)	Calcitriol (1.25(OH)2D)	Ergocalciferol (D2)	VDBP	Vit.D deficiency	Vit. B12	Red blood cell folate	Folate / Folic acid	Serum / Plasma Folate	Vit. B6 (PLP)	Hcy	Ancestry	Absolute Values	Effect size	Other	P-value	JBI assessment	Notes
Vitamin D	**VDR**<br>rs1544410	Gaffney-Stomberg et al., 2017	x											Caucasians, African Americans, other			Discovery cohort<br>β (SE)= 2.035 (0.674)	P = 0.0027	7/8	

Footnotes
Effect directions refer to minor allele T; reference group: GG (Wt)

	Gene and SNP	Source	Vitamin – Vitamin D: Calcifediol (25(OH)D)	Vitamin D: Calcitriol (1.25(OH)2D)	Vitamin D: Ergocalciferol (D2)	Vitamin D: VDBP	Vitamin D: Vit.D deficiency	Vit. B12	Vit. B9 (Folate): Red blood cell folate	Vit. B9 (Folate): Folate / Folic acid	Vit. B9 (Folate): Serum / Plasma Folate	Vit. B6 (PLP)	Hcy	Ancestry	Outcome: Absolute Values	Outcome: Effect size	Outcome: Other	Outcome: P-value	Risk of bias: JBI assessment	Notes
Vitamin D	**DAB1**<br>rs6680429 [1]	Engelman et al., 2010		x										Hispanic	*Mean ± SD [pmol/L]*<br><br>GG (Wt): 44.9 ± 15.3<br>GA: 45.8 ± 14.4<br>AA: 50.0 ± 18.3	GG (Wt):1<br>GA: 1.020<br>AA: 1.114		Subsample<br>P = 6.6E-9<br>Total sample<br>P = 1.4E-3	7/8	
	**N/A**<br>rs2806508	Engelman et al., 2010	x											Hispanic	*Mean ± SD [nmol/L]*<br><br>CC (Wt): 15.0 ± 6.5<br>CT: 16.4 ± 7.3<br>TT: 18.6 ± 8.0	CC (Wt): 1<br>CT: 1.093<br>TT: 1.240		Subsample<br>P = 1.6E-6<br>Total sample<br>P = 4.1E-5	7/8	
	**N/A**<br>rs10141935	Engelman et al., 2010	x											Hispanic	*Mean ± SD [nmol/L]*<br><br>AA (Wt): 16.1 ± 7.6<br>AG: 16.5 ± 7.0<br>GG: 18.2 ± 7.7	AA (Wt): 1<br>AG: 1.025<br>GG: 1.130		Subsample<br>P = 3.1E-5<br>Total sample<br>P = 3.9E-4	7/8	
	**N/A**<br>rs4778359	Engelman et al., 2010	x											Hispanic	*Mean ± SD [nmol/L]*<br><br>TT (Wt): 15.6 ± 6.9<br>TG: 18.0 ± 7.8<br>GG: 19.1 ± 7.7	TT (Wt): 1<br>TG: 1.154<br>GG: 1.224		Subsample<br>P = 4.0E-6<br>Total sample<br>P = 5.5E-5	7/8	
	**A2BP1**<br>rs1507023	Engelman et al., 2010	x											Hispanic	*Mean ± SD [nmol/L]*<br><br>TT (Wt): 17.0 ± 7.1<br>TC: 16.3 ± 7.6<br>CC: 15.8 ± 7.1	TT (Wt): 1<br>TC: 0.959<br>CC: 0.929		Subsample<br>P = 7.5E-6<br>Total sample<br>P = 5.1E-4	7/8	
	**GPR114**<br>rs9937918	Engelman et al., 2010	x											Hispanic	*Mean ± SD [nmol/L]*<br><br>CC (Wt): 17.0 ± 7.2<br>CT: 16.8 ± 7.2<br>TT: 15.4 ± 7.5	CC (Wt): 1<br>CT: 0.988<br>TT: 0.906		Subsample<br>P = 3.3E-5<br>Total sample<br>P = 8.2E-3	7/8	
	**N/A**<br>rs1348864	Engelman et al., 2010		x										Hispanic	*Mean ± SD [pmol/L]*<br><br>GG(Wt): 45.0 ± 14.7<br>GT: 45.8 ± 15.3<br>TT: 48.5 ± 17.9	GG (Wt):1<br>GT: 1.018<br>TT: 1.078		Subsample<br>P = 1.8E-5<br>Total sample<br>P = 1.5E-5	7/8	
	**N/A**<br>rs4559029	Engelman et al., 2010		x										Hispanic	*Mean ± SD [pmol/L]*<br><br>AA (Wt): 48.8 ± 16.0<br>AG: 45.2 ± 15.0<br>GG: 42.2 ± 15.3	AA (Wt): 1<br>AG: 0.926<br>GG: 0.865		Subsample<br>P = 1.5E-5<br>Total sample<br>P = 1.2E-4	7/8	
	**N/A**<br>rs12667374	Engelman et al., 2010		x										Hispanic	*Mean ± SD, [pmol/L]*<br><br>GG (Wt): 45.5 ± 15.2<br>GA: 55.4 ± 19.8<br>AA: 55.3 ± 30.6	GG(Wt):1<br>GA: 1.218<br>AA: 1.215		Subsample<br>P = 2.4E-5<br>Total sample<br>P = 1.8E-3	7/8	
	**MLL3**<br>rs7781309	Engelman et al., 2010		x										Hispanic	*Mean ± SD [pmol/L]*<br><br>TT (Wt): 45.2 ± 15.0<br>TC: 46.6 ± 16.1<br>CC: 53.8 ± 17.9	TT (Wt): 1<br>TC: 1.031<br>CC: 1.190		Subsample<br>P = 7.9E-6<br>Total sample<br>P = 3.1E-3	7/8	
	**N/A**<br>rs10505337	Engelman et al., 2010		x										Hispanic	*Mean ± SD [pmol/L]*<br><br>TT (Wt): 44.7 ± 14.7<br>TC: 46.3 ± 16.2<br>CC: 48.5 ± 15.3	TT (Wt): 1<br>TC: 1.036<br>CC: 1.085		Subsample<br>P = 2.0E-5<br>Total sample<br>P = 2.7E-3	7/8	
	**N/A**<br>rs2486443	Engelman et al., 2010		x										Hispanic	*Mean ± SD [pmol/L]*<br><br>GG (Wt): 45.7 ± 15.3<br>GA: 50.6 ± 18.2<br>AA: N/A	GG (Wt):1<br>GA: 1.107		Subsample<br>P = 2.4E-5<br>Total sample<br>P = 3.5E-4	7/8	
	**N/A**<br>rs2154175	Engelman et al., 2010		x										Hispanic	*Mean ± SD [pmol/L]*<br><br>GG (Wt): 44.0 ± 14.0<br>GT: 48.0 ± 16.3<br>TT: 52.7 ± 22.0	GG (Wt):1<br>GT: 1.091<br>TT: 1.198		Subsample<br>P = 1.5E-5<br>Total sample<br>P = 9.9E-4	7/8	

Footnotes
Effect directions refer to minor alleles; reference group: Wt
Absolute values apply to the total sample
These SNPs all were all significantly associated with Vit.D conc. In the study's GWA subsample (P < 1E-4, n= 229) and the entire cohort (P < 1E-2, n= 1190).
[1] rs9970802 and rs10889028 were in high LD with rs6680429 and were the only SNPs to meet Bonferroni corrected P-value cut off (P < 1.62E-7) in the GWA analysis.
Abbreviations: N/A, not applicaple is indicated for a Gene if the SNP is intragenic; LD, linkage disequilibrium

Vitamin D	**C10orf88**<br>rs6599638	Ahn et al., 2010 [1]	x											Caucasian	*Mean [nmol/L]*<br><br>ATBC<br>AA (Wt): 41.8<br>AG: 39.6<br>GG: 39.5<br><br>PLCO<br>AA (Wt): 60.3<br>AG: 57.8<br>GG: 57.6<br><br>NHS-CGEMS<br>AA (Wt): 83.3<br>AG: 83.0<br>GG: 77.5	*Mean [nmol/L]*<br><br>ATBC<br>AA (Wt): 1<br>AG: 0.947<br>GG: 0.945<br><br>PLCO<br>AA (Wt): 1<br>AG: 0.959<br>GG: 0.955<br><br>NHS-CGEMS<br>AA (Wt): 1<br>AG: 0.996<br>GG: 0.930	ATBC<br>β(SE): -0.19 (0.06)<br><br>PLCO<br>β(SE): -0.11 (0.05)<br><br>NHS-CGEMS<br>β(SE): -0.24 (0.08)	ATBC<br>P= 5.0E-4<br><br>PLCO<br>P= 2.3E-2<br><br>NHS-CGEMS<br>P= 2.2E-3	6/8	

Footnotes
Effect directions refer to minor allele G ; reference group: AA (wt)
[1] Ahn et al (2020) analyzed 5 GWAS and 3 case-control studies for associations between SNPs and 25(OH)D, the cohorts listed here include the GWAS that found significant results. Subjects include cases and contros.
Abbreviations: ATBC, Alpha- Tocopherol, Beta-Carotene Cancer Prevention Study; PLCO, Prostate, Lung, Colorectal, Ovarian Cancer Screening Trial; NHS-CGEMS, case–control studies of breast cancer (CGEMS) nested within the Nurses' Health Study (NHS)

	Gene and SNP	Source	Vitamin											Ancestry	Outcome				Risk of bias	Notes
			Vitamin D					Vit. B12	Vit. B9 (Folate)			Vit. B6 (PLP)	Hcy							
			Calcifediol (25(OH)D)	Calcitriol (1.25(OH)2D)	Ergocalciferol (D2)	VDBP	Vit.D deficiency		Red blood cell folate	Folate / Folic acid	Serum / Plasma Folate				Absolute Values	Effect size	Other	P-value	JBI assessment	
Vitamin D	**NADSYN1/DHCR7**<br>rs3829251	Ahn et al., 2010 [1,2]	**x**											Caucasian	ATBC<br>GG (Wt): 41.4<br>GA: 38.8<br>AA: 39.4<br><br>PLCO<br>GG (Wt): 59.3<br>GA: 56.2<br>AA: 53.0<br><br>NHS-CGEMS<br>GG (Wt): 83.3<br>GA: 77.8<br>AA: 81.3	ATBC<br>GG (Wt): 1<br>GA: 0.944<br>AA: 0.969<br><br>PLCO<br>GG (Wt): 1<br>GA: 0.948<br>AA: 0.894<br><br>NHS-CGEMS<br>GG (Wt): 1<br>GA: 0.934<br>AA: 0.976	ATBC<br>β(SE): -0.14 (0.06)<br><br>PLCO<br>β(SE): -0.22 (0.06)<br><br>NHS-CGEMS<br>β(SE): -0.28 (0.11)	ATBC<br>P= 2.0E-2<br><br>PLCO<br>P= 3.5E-4<br><br>NHS-CGEMS<br>P= 9.2E-3	6/8	
		Lu et al., 2011 [†]	**x**											Asian (Chinese)			GLR<br>Beijing: β (SE)= -0.066 (0.02)<br>Shanghai: β (SE)= -0.029 (0.01)<br><br>Conditional analysis<br>Beijing: β (SE)= -0.080 (0.04)	P= 3.1E-5<br>P= 0.0434<br><br>Conditional analysis<br>P= 0.0526	8/8	GLR<br>Analyses performed under additive model with adjusting for covariates.<br>Conditional analyses:<br>incl. all variants, which were in the same locus, in one multiple regression model.
		Slow et al., 2020	**x**											Caucasian			*Baseline 25(OH)D [nmol/L]*<br>β (95% CI): -7.30 (-12.35, -2.24)<br><br>*Baseline free 25(OH)D [pmol/L]*<br>β (95% CI): -3.76 (-6.46, -1.07) [3]	P= 0.005<br><br>P= 0.00658	12/13	[3] association remained significant after 2 mo of Vit. D3 supplementation

Footnotes

Effect directions refer to minor allele A ; reference group: GG (wt)

β = variation in serum 25(OH)D per risk-allele. Lu et al.(2011) refer to changes in log-25(OH)D

[†] Statistical analyses in these studies were performed on log-transformed 25(OH)D values.

[1] Ahn et al (2020) analyzed 5 GWAS and 3 case-control studies for associations between SNPs and 25(OH)D, the cohorts listed here include the GWAS that found significant results. Subjects include cases and contros.

[2] rs11234027 was r2=1 with NADSYN1 SNP rs3829251 in the HapMap CEU panel and significantly associated with 25(OH)D in one of the replication cohorts in Ahn et al., 2010 (cohort: HPFS)

Abbreviations: ATBC, Alpha- Tocopherol, Beta-Carotene Cancer Prevention Study; PLCO, Prostate, Lung, Colorectal, Ovarian Cancer Screening Trial; NHS-CGEMS, case–control studies of breast cancer (CGEMS) nested within the Nurses' Health Study (NHS)

Vitamin D	**NADSYN1/DHCR7**<br>rs12785878	Xu et al., 2015					**x**							Asian (Chinese)<br>Kazak ethnic group			**Odds for VDD**<br>OR(95%CI): 2.442 (1.224, 4.873)<br><br>**Allele Freq.**<br>VDD<br>GG: 0.23<br>GT: 0.55<br>TT: 0.22<br><br>Control<br>GG: 0.39<br>GT: 0.43<br>TT: 0.18	P = 0.011	8/8	VDD:<br>25(OH)D < 20ng/mL
		Wang et al., 2010 [1, †]	**x**											Caucasian	*Mean ± SE [nmol/L]*<br><br>1958 British Birth Cohort<br>Wt: 59.6 ± 0.31<br>het.: 56.3 ± 0.30<br>hom.: 55.7 ± 0.32	Wt: 1<br>het.: 0.945<br>Hom.: 0.935		*Candidate gene analysis*<br>Discovery Cohort<br>P = 1.27E-12<br>Replication Cohort<br>P = 2.39E-16<br>Overall<br>P = 2.12-27	8/8	
		Grant et al., 2022	**x**											Mixed		*Adjusted Mean Difference (95%CI), [nmol/L]*<br><br>GG: Ref. (0.00)<br>GT: 4.22 (1.74, 6.70)<br>TT: 8.06 (5.42, 10.69)		Both P < 0.001	7/8	
		Slow et al., 2020	**x**											Caucasian			*Baseline 25(OH)D [nmol/L]*<br>β (95% CI): -5.42 (-9.24, -1.59)<br><br>*Baseline free 25(OH)D [pmol/L]*<br>β (95% CI): -3.50 (-5.52, -1.47) [2]<br><br>*SNP * Vit.D supplementation on free 25(OH)D [pmol/L]*<br>β (95% CI): -8.86 (-13.95, -3.76)	P= 0.00591<br><br>P= 0.00080 [3]<br><br>P= 0.00071 [3]	12/13	[2] association remained significant after 2 mo of Vit. D3 supplementation<br><br>[3] remained signif. After Bonferroni correction
		Kwak et al., 2018	**x**											Asian (Korean)			"found negative associations btw serum 25(OH)D level and the effect allele T"	P < 0.001	7/8	

Footnotes

Effect directions refer to minor allele T; reference group: GG (wt)

β = variation in serum 25(OH)D per risk-allele

[†] Statistical analyses in these studies were performed on log-transformed 25(OH)D values.

[1] rs7944926 was a perfect proxy in this cohort (r2=1.0) and results can be found in their original paper

Table S11: Antioxidants parameter study

	Gene and SNP	Source	Antioxidant: Selenium	Antioxidant: Selenoprotein P	Antioxidant: Alpha-carotene	Antioxidant: Beta-carotene	Antioxidant: Alpha-tocopherol	Ancestry	Outcome: Absolute values and Mean Difference	Outcome: Effect size	Outcome: Other	Outcome: P-value	Notes
Antioxidants	**BCO1**<br>rs6564851	Yabuta et al., 2016				x		Asian (Japanese)	*Mean ± SD [µg/dL]*<br>GG: 63.9 ± 52.6<br>GT+TT: 31.2 ± 23.4<br>MD ± SD: 32.7 ± 57.57	GG: 1<br>GT + TT: 0.488<br>*Cohen's d* = 0.568		0.0032	
		Ferrucci et al., 2009				x		Caucasian		*Effect (95%CI)*<br>InCHIANTI: 0.160 (0.112, 0.208)<br>WHAS: 0.199 (0.116, 0.282)<br>ATBC: 0.13 (0.092, 0.171)<br>Meta-analysis: 0.149 (0.120, 0.177)	*Standard error (SE)*<br>0.024<br>0.042<br>0.020<br>0.015<br>Variance explained: 1.9% [a]	9.7E-11<br>2.6E-6<br>1.1E-10<br>1.6E-24	Paper states that "Participants with a G allele had an average b-carotene level 0.26 (0.18, 0.34) standard deviations higher than participants with a T allele."
					x					*Effect (95%CI)*<br>InCHIANTI: 0.089 (0.035, 0.143)<br>WHAS: 0.091 (0.003, 0.185)<br>Meta-analysis: 0.089 (0.043, 0.136)	*Standard error (SE)*<br>0.027<br>0.048<br>0.024	0.001<br>0.057<br>0.0001	

Footnotes
Minor allele frequencies vary depending on ancestry.
Effect directions for Yabuta et al (2016) refer to minor allele T; reference genotype: GG (wt) while effect directions for Ferrucci et al (2009) refer to minor allele G; reference genotype: TT (wt)
Effect sizes by Ferruci et al. (2009) were calculated with natural log-transformed concentrations.
Calculations to obtain Cohen's d are presented in the *Calculations* sheet
[a] based on ATBC cohort

	Gene and SNP	Source	Selenium	Selenoprotein P	Alpha-carotene	Beta-carotene	Alpha-tocopherol	Ancestry	Absolute values and Mean Difference	Effect size	Other	P-value	Notes
Antioxidants	**BCO1**<br>rs6420424	Ferrucci et al., 2009				x		Caucasian		*Effect (95%CI)*<br>InCHIANTI: 0.152 (0.103, 0.201)<br>WHAS: 0.165 (0.082, 0.248)<br>Meta-analysis: 0.155 (0.11, 0.198)	*Standard error (SE)*<br>0.025<br>0.042<br>0.022	1.9E-9<br>1.0E-4<br>6.5E-13	

Footnotes
Effect directions refer to minor allele A; reference genotype: GG (wt)
Effect sizes were calculated with natural log-transformed concentrations.
rs6420424 failed genotyping in the ATBC study, therefore no values are presented for this cohort

	Gene and SNP	Source	Selenium	Selenoprotein P	Alpha-carotene	Beta-carotene	Alpha-tocopherol	Ancestry	Absolute values and Mean Difference	Effect size	Other	P-value	Notes
Antioxidants	**BCO1**<br>rs8044334	Ferrucci et al., 2009				x		Caucasian		*Effect (95%CI)*<br>InCHIANTI: 0.161 (0.106, 0.216)<br>WHAS: 0.169 (0.084, 0.255)<br>ATBC: 0.07 (0.034, 0.114)<br>Meta-analysis: 0.109 (0.079, 0.139)	*Standard error (SE)*<br>0.028<br>0.044<br>0.02<br>0.015	1.3E-8<br>1.1E-4<br>0.0003<br>9.3E-13	

Footnotes
Effect directions refer to minor allele G; reference genotype: TT (wt)
Effect sizes were calculated with natural log-transformed concentrations.

	Gene and SNP	Source	Selenium	Selenoprotein P	Alpha-carotene	Beta-carotene	Alpha-tocopherol	Ancestry	Absolute values and Mean Difference	Effect size	Other	P-value	Notes
Antioxidants	**BCO1**<br>rs11645428	Ferrucci et al., 2009				x		Caucasian		*Effect (95%CI)*<br>InCHIANTI: -0.118 (-0.165, -0.072)<br>WHAS: -0.196 (-0.279, -0.113)<br>ATBC: -0.12 (-0.160, 0.075)<br>Meta-analysis: -0.129 (-0.159, -0.099)	*Standard error (SE)*<br>0.024<br>0.042<br>0.022<br>0.015	8.3E-7<br>3.7E-6<br>7.4E-8<br>1.5E-17	

Footnotes
Effect directions refer to minor allele A; reference genotype: GG (wt)
Effect sizes were calculated with natural log-transformed concentrations.

	Gene and SNP	Source	Selenium	Selenoprotein P	Alpha-carotene	Beta-carotene	Alpha-tocopherol	Ancestry	Absolute values and Mean Difference	Effect size	Other	P-value	Notes
Antioxidants	**APOA**<br>rs12272004	Ferrucci et al., 2009					x	Caucasian		*Effect (95%CI)*<br>InCHIANTI: 0.11 (0.066, 0.15)<br>WHAS: 0.121 (0.048, 0.194)<br>ATBC: 0.047 (0.017, 0.077)<br>Meta-analysis: 0.072 (0.049, 0.095) [a]	*Standard error (SE)*<br>0.022<br>0.038<br>0.015<br>0.012	3.9E-7<br>0.001<br>0.0019<br>7.8E-10	

Footnotes
Effect directions refer to minor allele A; reference genotype: CC (wt)
Effect sizes were calculated with natural log-transformed concentrations.
[a] Unadjusted values; values adjusted for TAG: Effect (95% CI): 0.055 (0.02, 0.091), P= 0.002

	Gene and SNP	Source	Selenium	Selenoprotein P	Alpha-carotene	Beta-carotene	Alpha-tocopherol	Ancestry	Absolute values and Mean Difference	Effect size	Other	P-value	Notes
Antioxidants	**SELENOP**<br>rs3877899	Méplan et al., 2007	x					Caucasian & Asian	NR		"SNP affected plasma Se conc. postsupplementation and 2 wk after Se withdrawal."	0.012 *<br>0.001 **	* after 6 weeks of 100 µg Sodium selenite/ day n<br>** 2 weeks after supplementation withdrawal
				x					*Mean ± SE [µg/mL plasma]*<br><u>Women</u><br>GG (Wt) : 5<br>GA: 4.7<br><u>Men</u><br>GG (Wt): 4.2<br>GA: 5.1<br>MD ± SD by gender [µg/mL]<br>GA: -0.4 ± 2.79<br>GG: 0.8 ± 2.42	*Comparison by gender*<br>*GA Cohen's d* = -0.14<br>*GG Cohen's d* = 0.33		<u>Gender effect</u><br>P = 0.028	Absolute values are estimated based on Figure 3A in Méplan et al. (2007)
		Combs et al., 2011		x				NR	*Geometric Mean (-1SD, + 1SD) [ng/mL]*<br>GG (Wt): 3.62 (2.75, 4.76)<br>GA: 3.24 (2.44, 4.31)<br>AA: 3.49 (2.91, 4.18)	GG (Wt): 1<br>GA: 0.895<br>AA: 0.964		NR	No P-value reported, but the difference between GG and GA was stated as significant in the text
		Kopp et al., 2018 [a]		x				Caucasian	**At week 26**<br>*MD (95%CI) CC vs.CT+TT [ng/mL]*<br>-4.68 (8.49, -0.871)			0.018	Model incl. unsupplemented control subjects: P = 0.048
			x						**At week 26**<br>*CC vs.CT+TT [ng/mL]*<br>MD (95%CI): -5.76 (-12.5, 1.01)			0.093	
		Batai et al., 2021	x					Caucasian		β (SE): -0.011 (0.004) [b]		0.002	

Footnotes
Effect directions refer to minor allele T (A on reverse DNA strand); reference genotype: CC (wt) (GG on reverse strand)
Calculations to obtain Cohen's d are presented in the *Calculations* sheet
Values reported as geometric mean with a 1 SD interval were logarithmically transformed to approximated normal distribution.
[a] Supplementation: ca. 50.3µg selenium/day for 26wks
[b] Association significant at baseline, but not after 1 year of supplementating 200 µg of selenized yeast/ day
Abbreviations: MD, mean difference; NR, Not reported; SE, standard error

	Gene and SNP	Source	Selenium	Selenoprotein P	Alpha-carotene	Beta-carotene	Alpha-tocopherol	Ancestry	Absolute values and Mean Difference	Effect size	Other	P-value	Notes
Antioxidants	**GPX1**<br>rs1050450	Combs et al., 2011	x					NR	*Mean ± SD [ng/mL]*<br>CC (Wt): 145.9 ± 24.3<br>CT: 139.5 ± 23.1<br>TT: 135.7 ± 19	CC (Wt): 1<br>CT: 0.956<br>TT: 0.930<br>*CT Cohen's d* = -0.191<br>*TT Cohen's d* = -0.330		NR	No P-value reported, but the difference between TT and CT was stated as significant in the text
		Batai et al., 2021	x					Caucasian		β (SE): 0.010 (0.003) [a]		0.004	

Footnotes
Effect directions refer to minor allele A (T on reverse DNA strand); reference genotype: GG (wt) (CC on reverse strand)
Calculations to obtain Cohen's d are presented in the *Calculations* sheet
[a] Association significant at baseline, but not after 1 year of supplementating 200 µg of selenized yeast/ day
Abbreviations: MD, mean difference; NR, Not reported

Cohen's d calculations

rs3877899: Plasma SePP conc. - Méplan, 2007	Genotype 1: GA women	Genotype 2: GA men
Mean Value (µg/mL)	4.7	5.1
n	42	33
Standard Error (SE) Formula	SD/ sqrt(n)	
SE	0.3	0.35
Standard Deviation (SD) Formula	SE x sqrt(n)	
SD	1.94422221	2.010596926
Mean SD Formula	Sqrt(SD1^2 + SD2^2)	
Mean SD	2.796873254	
Effect size Formula	(Mean 1-Mean 2)/Mean SD	
Effect size	-0.143016849	

rs3877899: Plasma SePP conc. - Méplan, 2007	Genotype 1: GG women	Genotype 2: GG men
Mean Value(µg/mL)	5	4.2
n	42	33
Standard Error (SE) Formula	SD/ sqrt(n)	
SE	0.3	0.25
Standard Deviation (SD) Formula	Sqrt(SD1^2 + SD2^2)	
SD	1.94422221	1.436140662
Mean SD Formula	(SD 1+ SD 2) / 2	
Mean SD	2.417126393	
Effect size Formula	(Mean 1-Mean 2)/Mean SD	
Effect size	0.330971522	

rs1050450: plasma Se - Combs et al., 2011	Genotype 1: CC	Genotype 2: CT
Mean Value (ng/mL)	145.9	139.5
n	119	113
Standard Error (SE) Formula	SD/ sqrt(n)	
SE		
Standard Deviation (SD) Formula	SE x sqrt(n)	
SD	24.3	23.1
Mean SD Formula	Sqrt(SD1^2 + SD2^2)	
Mean SD	33.52760057	
Effect size Formula	(Mean 1-Mean 2)/Mean SD	
Effect size	-0.190887504	

rs1050450: plasma Se - Combs et al., 2011	Genotype 1: TT	Genotype 2: CC
Mean Value (ng/mL)	135.7	145.9
n	28	119
Standard Error (SE) Formula	SD/ sqrt(n)	
SE		
Standard Deviation (SD) Formula	SE x sqrt(n)	
SD	19	24.3
Mean SD Formula	Sqrt(SD1^2 + SD2^2)	
Mean SD	30.84623154	
Effect size Formula	(Mean 1-Mean 2)/Mean SD	
Effect size	-0.330672484	

rs6564851: B-carotene - Yabuta et al., 2016	GT+TT	GG
Mean Value (µg/dL)	31.2	63.9
n	-	-
Standard Error (SE) Formula	SD/ sqrt(n)	
SE		
Standard Deviation (SD) Formula	SE x sqrt(n)	
SD	23.4	52.6
Mean SD Formula	Sqrt(SD1^2 + SD2^2)	
Mean SD	57.57013114	
Effect size Formula	(Mean 1-Mean 2)/Mean SD	
Effect size	-0.568002875	
